# Simple synthesis of pyrrolo[3,2-*e*]indole-1-carbonitriles

**DOI:** 10.3762/bjoc.9.107

**Published:** 2013-05-15

**Authors:** Adam Trawczyński, Robert Bujok, Zbigniew Wróbel, Krzysztof Wojciechowski

**Affiliations:** 1 Institute of Organic Chemistry, Polish Academy of Sciences, ul. Kasprzaka 44/52, POBox 58, 01-224 Warszawa, Poland, Fax: +48 (22) 632 66 81; 2 Department of Chemistry, Warsaw University of Technology, ul. Noakowskiego 3, 00-664 Warszawa, Poland

**Keywords:** alkylation, ketones, nitriles, pyrroloindole, reduction, trimethylchlorosilane

## Abstract

Alkylation of 5-nitroindol-4-ylacetonitriles with ethyl chloroacetate, α-halomethyl ketones, and chloroacetonitrile followed by a treatment of the products with chlorotrimethylsilane in the presence of DBU gives 1-cyanopyrrolo[3,2-*e*]indoles substituted in position 2 with electron-withdrawing groups.

## Introduction

**Scheme 1:**
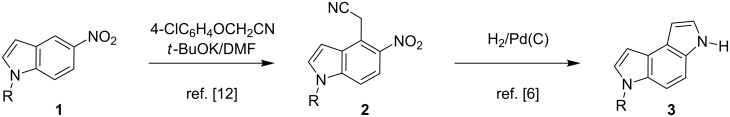
Synthesis of pyrrolo[3,2-*e*]indoles via VNS in 5-nitroindoles [6,12].

Indole and its analogues bearing condensed arene and heteroarene rings are privileged structures amongst biologically active compounds. The 1,2-dihydropyrrolo[3,2-*e*]indole fragment is present in anticancer agents, such as CC-1065 [1], duocarmycin [1], and yatakemycin [2]. Some pyrrolo[3,2-*e*]indole derivatives show antimicrobial activity [3]. One method of synthesis of the 1,2-dihydropyrrolo[2,3-*e*]indoles is reduction of pyrrolo[3,2-*e*]indoles with sodium cyanoborohydride [4]. On the other hand there are many methods of synthesis of pyrrolo[3,2-*e*]indoles such as the copper-catalyzed transformation of tetrahydroquinoline derivatives [4], photochemical cyclization of 1,2-bis(2-pyrrolo)ethylenes [5], the Fischer indole synthesis from indol-5-ylhydrazones [3], or a palladium-catalyzed hydrogenation of 5-nitroindol-4-ylacetonitriles **2** [6]. In the latter synthesis of pyrrolo[3,2-*e*]indole **3** the starting nitrile **2** was obtained by the vicarious nucleophilic substitution (VNS) [7–11] of hydrogen in 1-alkyl-5-nitroindole **1** with 4-chlorophenoxyacetonitrile [12] (Scheme 1).

In our previous papers [13–16] we have shown that *o*-nitroarylacetonitriles alkylated and alkenylated at the α-position to the cyano group can be converted into indoles under basic conditions in the presence of a silylating agent.

## Results and Discussion

**Scheme 2:**
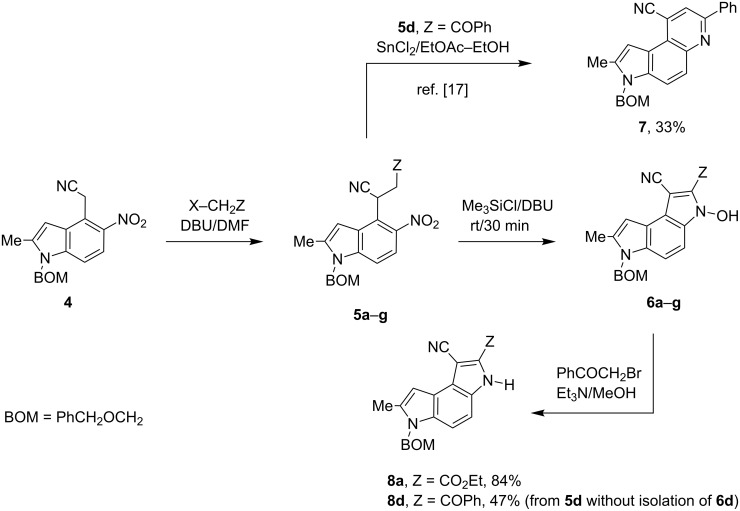
Synthesis of pyrrolo[3,2-*e*]indoles **6**.

Here we report a simple two-step procedure for the transformation of 5-nitroindol-4-ylacetonitriles into pyrrolo[3,2-*e*]indole-1-carbonitriles **6** bearing an additional electron-withdrawing substituent at position 2. In our approach the starting material was 1-benzyloxymethyl-4-cyanomethyl-2-methyl-5-nitroindole (**4**) obtained via the VNS of hydrogen in 1-benzyloxymethyl-2-methyl-5-nitroindole with 4-chlorophenoxyacetonitrile according to our earlier elaborated method [12]. Alkylation of the nitrile **4** with ethyl bromoacetate in the presence of K_2_CO_3_ led to the expected cyanoester **5a** in 68% yield, but the product contained some contaminants difficult to separate by crystallization or column chromatography. Searching for more convenient reaction conditions, we have found that this reaction proceeds satisfactorily in almost quantitative yield when diazabicycloundecene (DBU) was used as the base. Analogous alkylation with α-halomethyl ketones, chloroacetonitrile, chloroacetamide and cinnamyl bromide provided the expected alkylation products **5b**–**g** in good yields (Scheme 2 and Table 1).

**Table 1:** Alkylation products **5** and synthesized 1-cyano-3-hydroxy-pyrrolo[3,2-*e*]indoles **6**.

Entry	X–CH_2_–Z	Indole **5**	Yield (%)	Pyrrolo[3,2-*e*]indole **6**	Yield (%)

1	Br–CH_2_CO_2_Et	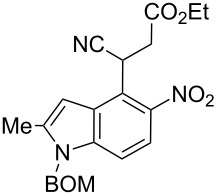 **5a**	99	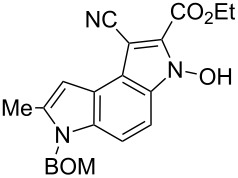 **6a**	90
2	Cl–CH_2_COMe	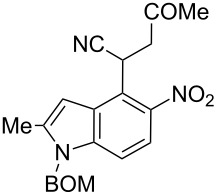 **5b**	88	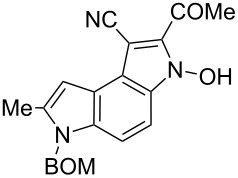 **6d**	61
3	Cl–CH_2_COCMe_3_	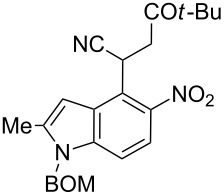 **5c**	82	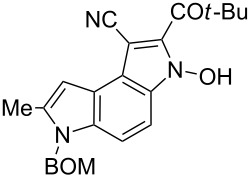 **6c**	55
4	Br–CH_2_COPh	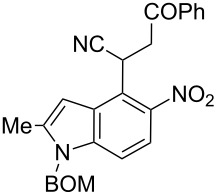 **5d**	98	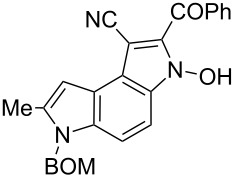 **6d**	30
5	Cl–CH_2_CN	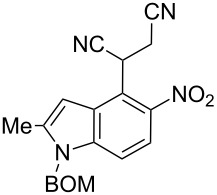 **5e**	86	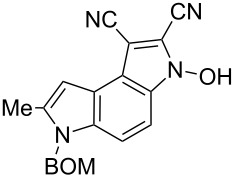 **6e**	30
6	Cl–CH_2_CONMe_2_	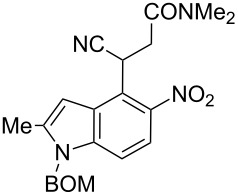 **5f**	95	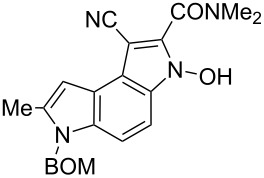 **6f**	44
7	Br–CH_2_CH=CH_2_Ph	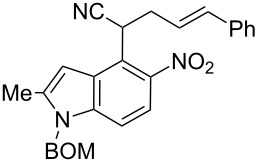 **5g**	50	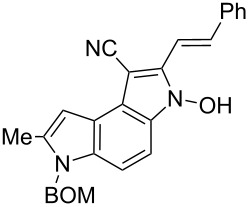 **6g**	25

To find optimal conditions for cyclization of the model compound **5a** we screened various combinations of base and a reagent promoting the cyclization. With chlorotrimethylsilane–triethylamine the reaction proceeded slowly, and the starting material was completely consumed after 24 h, but the product **6a** was isolated in moderate 30% yield. However, when we replaced triethylamine with a stronger base, such as DBU, the reaction was completed in 30 min, and the product was isolated in 90% yield. Similarly, with *N*,*O*-bis(trimethylsilyl)acetamide (BSA) the reaction was completed in 30 min giving **6a** in 67% yield. With tributylchlorostannane combined with DBU the reaction proceeded slowly to form after 24 h product **6a** in 72% yield. Methanesulfonyl and pivaloyl chlorides, in combination with DBU proved ineffective in this reaction giving a very low rate of conversion after 24 h. Thus, transformations of other nitriles **5b**–**g** into pyrrolo[3,2-*e*]indoles **6** were performed in the DBU–chlorotrimethylsilane system, and the results are presented in Table 1. It is worth mentioning that the ketone **5d** upon reduction with SnCl_2_ cyclized to pyrrolo[3,2-*f*]quinoline-9-carbonitrile **7** [17].

The removal of the benzyloxymethyl group from 1-(benzyloxymethyl)pyrrolo[3,2-*e*]indoles by catalytic hydrogenation has been described by Macor [6]. The hydroxy group from the *N*-hydroxypyrrole fragment can be removed by a procedure elaborated by us [18] employing α-bromoacetophenone in the presence of triethylamine as exemplified for pyrroloindoles **6a** and **6d** that were transformed under these conditions into the corresponding derivatives **8a** and **8d** (Scheme 2). The crude 3-hydroxy-pyrrolo[3,2-*e*]indole **6d** without isolation and purification was subjected to dehydroxylation giving compound **8d** in 47% yield.

**Scheme 3:**
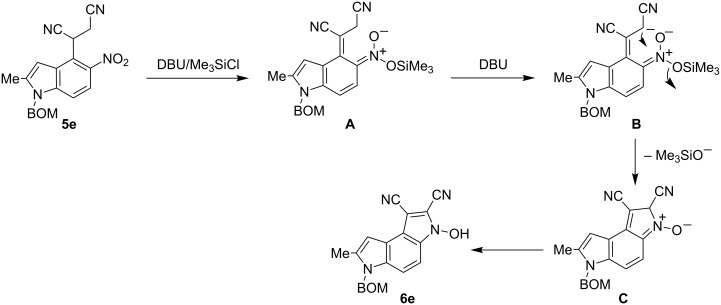
Plausible route for transformation of indoles **5** into pyrrolo[3,2-*e*]indoles **6**.

A plausible route to the formation of 3-hydroxy-1-cyanopyrrolo[3,2-*e*]indoles is exemplified by the synthesis of 1,2-dicyano derivative **6e** from the dinitrile **5e** (Scheme 3). In the first step the *o*-nitrobenzylic carbanion is silylated with trimethylchlorosilane to form trimethylsilyl nitronate **A**. Then a consecutive deprotonation forms another carbanion **B** at the β-position to the ring. The attack of this carbanion on the trimethylsilylnitronate results in the substitution of trimethoxysiloxyl and formation of **C** in that the five-membered ring finally isomerizes to the *N*-hydroxypyrrole fragment of **6e**.

**Scheme 4:**
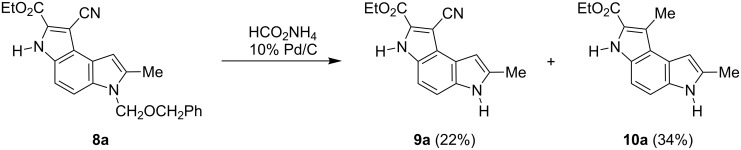
Removal of the benzyloxymethyl group from the compound **8a**.

To remove the benzyloxymethyl group from the compound **8a** we adopted the procedure proposed by Macor [6]. Heating **8a** with ammonium formate and 10% palladium on carbon as a catalyst in isopropanol in a sealed tube (95 °C) led to a mixture of the expected product **9a** and the product **10a** in that the cyano group was reduced to a methyl substituent (Scheme 4). There is a literature precedence [19] for similar transformations of cyanoarenes into corresponding methyl derivatives upon transfer hydrogenation with ammonium formate in the presence of palladium on a carbon catalyst.

## Conclusion

In conclusion, the approach presented herein can be useful for the synthesis of variously substituted pyrrolo[3,2-*e*]indoles. The method does not require reductive conditions for the formation of the pyrrole ring and, thus, can be applicable for derivatives bearing sensitive substituents.

## Experimental

Melting points (mp) are uncorrected. ^1^H and ^13^C NMR spectra were recorded on a Bruker Avance 500 or Varian vnmr s500 (both 500 MHz for ^1^H and 125 MHz for ^13^C spectra) instruments at 298 K. Chemical shifts δ are expressed in parts per million referenced to TMS; coupling constants *J* in hertz. IR spectra were recorded in KBr on a Perkin Elmer PE Spectrum 2000 spectrometer. Electron impact mass spectra (EI, 70 eV) were obtained on AMD-604 and AutoSpec Premier spectrometer. Electrospray mass spectra (ESI) were obtained on 4000 Q-TRAP and SYNAPT G2-S HDMS. Silica gel (Merck 60, 230–400 mesh) was used for column chromatography (CC). All reagents and solvents were of reagent grade or purified according to standard methods before use. 1-Benzyloxymethyl-4-(cyanomethyl)-2-methyl-5-nitroindole (**4**) was obtained by VNS of hydrogen in 1-benzyloxymethyl-2-methyl-5-nitroindole with 4-chlorophenoxyacetonitrile following our previously elaborated method [12].

### 

#### Alkylation of indolylacetonitrile **4** with ethyl bromoacetate. Synthesis of 3-(1-benzyloxymethyl-2-methyl-5-nitro-1*H*-indol-4-yl)-3-cyanopropionic acid ethyl ester (**5a**) – Typical procedure

A solution of (1-benzyloxymethyl-2-methyl-5-nitro-1*H*-indol-4-yl)acetonitrile (**4**, 0.335 g, 1 mmol) and ethyl bromoacetate (0.25 g, 1.5 mmol) were stirred in DMF (5 mL) and DBU (0.30 g, 2 mmol) at 60 °C until the starting material **4** disappeared (usually 24 h, TLC control). Then the reaction mixture was poured into diluted HCl and the product was extracted with EtOAc (3 × 30 mL) and dried with Na_2_SO_4_. After evaporation of the solvent the residue was purified by column chromatography (SiO_2_, hexane–EtOAc, 2:1). Yellow crystals; mp 76–78 °C; ^1^H NMR (500 MHz, CDCl_3_) δ 1.28 (t, *J* = 7.3 Hz, 3H), 2.52 (s, 3H), 3.06 (dd, *J* = 16.9, 6.1 Hz, 1H), 3.28 (dd, *J* = 16.9, 9.0 Hz, 1H), 4.21 (q, *J* = 7.3 Hz, 2H), 4.47 (s, 2H), 5.42 (dd, *J* = 9.0, 6.1 Hz, 1H), 5.55 (s, 2H), 6.80 (br s, 1H), 7.23–7.27 (m, 2H), 7.31–7.37 (m, 4H), 7.84 (d, *J* = 9.0 Hz, 1H); ^13^C NMR (125 MHz, CDCl_3_) δ 12.82, 14.10, 27.65, 37.67, 61.66, 70.12, 71.96, 102.16, 109.93, 118.79, 119.02, 120.50, 126.92, 127.66, 128.29, 128.63, 136.43, 139.48, 141.32, 142.22, 168.98; IR (KBr, cm^−1^) ν: 2979, 2243, 1728, 1603, 1561, 1510, 1461, 1450, 1409, 1373, 1339, 1272, 1244, 1205, 1188, 1130, 1096, 959, 813, 800, 769, 759, 741; EIMS (70 eV) *m*/*z* (% relative intensity): 421 (14) [M] ^+∙^, 404 (5), 92 (8), 91 (100); HRMS–EI (70 eV, *m*/*z*): [M]^+^ calcd for C_23_H_23_N_3_O_5_, 421.1638; found, 421.1630.

**2-(1-Benzyloxymethyl-2-methyl-5-nitro-1*H*-indol-4-yl)-4-oxopentanenitrile (5b)**. Yellow crystals; mp 102–104 °C; ^1^H NMR (500 MHz, CDCl_3_) δ 2.21 (s, 3H), 2.52 (d, *J* = 1.0 Hz, 3H), 3.12 (dd, *J* = 18.3, 5.2 Hz, 1H), 3.50 (dd, *J* = 18.3, 8.7 Hz, 1H), 4.47 (s, 2H), 5.37 (dd, *J* = 8.7, 5.2 Hz, 1H), 5.54 (s, 2H), 6.77 (br s, 1H), 7.24–7.27 (m, 2H), 7.31–7.37 (m, 4H), 7.83 (d, *J* = 8.9 Hz, 1H); ^13^C NMR (125 MHz, CDCl_3_) δ 12.80, 26.20, 29.42, 46.23, 70.06, 71.92, 102.12, 109.73, 118.82, 119.21, 121.08, 127.03, 127.64, 128.25, 128.62, 136.39, 139.40, 141.21, 142.19, 202.77; IR (KBr, cm^−1^) ν: 2915, 2250, 2240, 1714, 1607, 1559, 1517, 1504, 1451, 1400, 1330, 1257, 1238, 1170, 1081, 1060, 1006, 948, 817, 733; EIMS (70 eV) *m*/*z* (% relative intensity): 391 (3) [M]^+∙^, 357 (11), 92 (8), 91 (100); HRMS–EI (70 eV, *m*/*z*): [M]^+^ calcd for C_22_H_21_N_3_O_4_, 391.1532; found, 391.1540.

**2-(1-Benzyloxymethyl-2-methyl-5-nitro-1*H*-indol-4-yl)-5,5-dimethyl-4-oxohexanenitrile (5c)**. Yellow crystals; mp 92–94 °C; ^1^H NMR (500 MHz, CDCl_3_) δ 1.15 (s, 9H), 2.53 (d, *J* = 0.8 Hz, 3H), 3.14 (dd, *J* = 17.9, 4.9 Hz, 1H), 3.57 (dd, *J* = 17.9, 9.1 Hz, 1H), 4.47 (s, 2H), 5.32 (dd, *J* = 9.1, 4.9 Hz, 1H), 5.55 (s, 2H), 6.78 (br s, 1H), 7.24–7.37 (m, 6H), 7.83 (d, *J* = 9.0 Hz, 1H); ^13^C NMR (125 MHz, CDCl_3_) δ 12.84, 26.11, 26.91, 40.21, 44.06, 70.05, 71.92, 102.10, 109.66, 118.83, 119.42, 121.47, 127.10, 127.65, 128.26, 128.62, 136.40, 139.37, 141.18, 142.21, 210.44; IR (KBr, cm^−1^) ν: 2969, 2244, 1707, 1606, 1652, 1519, 1477, 1340, 1071, 1029, 818, 757, 740; EIMS (70 eV) *m*/*z* (% relative intensity): 433 (3) [M]^+∙^, 399 (6), 92 (8), 91 (100); HRMS–EI (70 eV, *m*/*z*): [M]^+^ calcd for C_25_H_27_N_3_O_4_, 433.2002; found, 433.2004.

**2-(1-Benzyloxymethyl-2-methyl-5-nitro-1*H*-indol-4-yl)-4-oxo-4-phenylbutyronitrile (5d)**. Yellow crystals; mp 123–125 °C; ^1^H NMR (500 MHz, CDCl_3_) δ 2.52 (d, *J* = 1.0 Hz, 3H), 3.68 (dd, *J* = 18.0, 5.1 Hz, 1H), 4.05 (dd, *J* = 18.0, 8.7 Hz, 1H), 4.47 (s, 2H), 5.55 (s, 2H), 5.58 (dd, *J* = 8.7, 5.1 Hz, 1H), 6.83 (m, 1H), 7.25 (s, 1H), 7.26 (s, 1H), 7.30–7.37 (m, 4H), 7.44–7.48 (m, 2H), 7.56–7.60 (m, 1H), 7.85 (d, *J* = 9.0 Hz, 1H), 7.93–7.96 (m, 2 H); ^13^C NMR (125 MHz, CDCl_3_) δ 12.86, 26.64, 42.01, 70.09, 71.96, 102.24, 109.78, 118.88, 119.46, 121.36, 127.15, 127.67, 128.17, 128.27, 128.64, 128.78, 133.85, 135.57, 136.44, 139.45, 141.27, 142.37, 194.46; IR (KBr, cm^−1^) ν: 2921, 2246, 1690, 1559, 1518, 1447, 1343, 1213, 1086, 1071, 803, 751, 689; EIMS (70 eV) *m*/*z* (% relative intensity): 453 (2) [M]^+∙^, 419 (9), 105 (35), 92 (8), 91 (100), 77 (13); HRMS–EI (70 eV, *m*/*z*): [M]^+^ calcd for C_27_H_23_N_3_O_4_, 453.1689; found, 453.1671.

**2-(1-Benzyloxymethyl-2-methyl-5-nitro-1*H*-indol-4-yl)-succinonitrile (5e)**. Brown oil; ^1^H NMR (500 MHz, CDCl_3_) δ 2.54 (d, *J* = 1.0 Hz, 3H), 3.25, 3.31, 5.34 (ABX, *J* = 17.0, 8.0, 6.8 Hz, 3H), 4.48 (s, 2H), 5.56 (s, 2H), 6.87 (br s, 1H), 7.24–7.27 (m, 2H), 7.31–7.37 (m, 3H), 7.41 (d, *J* = 8.9 Hz, 1H), 7.93 (d, *J* = 8.9 Hz, 1H); ^13^C NMR (125 MHz, CDCl_3_) δ 12.88, 22.04, 28.75, 70.20, 71.98, 102.00, 110.78, 115.41, 117.06, 118.19, 119.09, 127.02, 127.65, 128.31, 128.64, 136.25, 139.80, 141.77, 142.14; IR (KBr, cm^−1^) ν: 2949, 2249, 2225, 1607, 1562, 1519, 1446, 1399, 1337, 1071, 821, 738, 700; EIMS (70 eV) *m*/*z* (% relative intensity): 374 (11) [M]^+∙^, 340 (8), 92 (14), 91 (100), 65 (8); HRMS–EI (70 eV, *m*/*z*): [M]^+^ calcd for C_21_H_18_N_4_O_3_, 374.1379; found, 374.1385.

**3-(1-Benzyloxymethyl-2-methyl-5-nitro-1*H*-indol-4-yl)-3-cyano-*N*,*N*-dimethylpropionamide (5f)**. Orange oil; ^1^H NMR (500 MHz, CDCl_3_) δ 2.52 (s, 3H), 2.97 (br s, 1H), 2.96 (s, 3H), 2.98 (s, 3H), 3.40 (dd, *J* = 16.4, 8.8, Hz, 1H), 4.47 (s, 2H), 5.44 (dd, *J* = 8.8, 5.4 Hz, 1H), 5.54 (s, 2H), 6.81 (m, 1H), 7.25–7.30 (m, 2H), 7.31–7.37 (m, 4H), 7.83 (d, *J* = 9.0 Hz, 1H); ^13^C NMR (100 MHz, CDCl_3_) δ 12.83, 28.10, 35.69, 36.93, 36.99, 70.07, 71.94, 102.26, 109.66, 118.88, 119.58, 121.67, 127.32, 127.66, 128.26, 128.64, 136.45, 139.39, 141.10, 142.39, 167.99; IR (KBr, cm^−1^) ν: 2932, 2244, 1651, 1561, 1519, 1400, 1340, 1267, 1241, 1150, 1070, 822, 737, 700; EIMS (70 eV) *m*/*z* (% relative intensity): 420 (2) [M]^+∙^, 375 (6), 374 (16), 195 (8), 108 (8), 107 (6), 92 (12), 91 (49); HRMS–EI (70 eV, *m*/*z*): [M]^+^ calcd for C_23_H_24_N_4_O_4_, 420.1798; found, 420.1796.

**2-(1-Benzyloxymethyl-2-methyl-5-nitro-1*H*-indol-4-yl)-5-phenylpent-4-enenitrile (5g)**. Yellow crystals; mp 130–132 °C; ^1^H NMR (500 MHz, CDCl_3_) δ 2.52 (s, 3H), 2.98 (ddd, *J* = 13.8, 9.1, 7.5 Hz, 1H), 3.13 (ddd, *J* = 13.8, 7.5, 6.0 Hz, 1H), 5.09 (dd, *J* = 9.1, 6.0 Hz, 1H), 6.31 (ddd, 15.4, 7.5, 7.5 Hz, 1H), 6.58 (d, *J* = 15.4 Hz, 1H), 6.90 (s, 1H), 7.21–7.40 (m, 11H), 7.85 (d, *J* = 8.8 Hz, 1H); ^13^C NMR (100 MHz, CDCl_3_) δ 12.82, 32.17, 37.05, 70.06, 71.94, 102.88, 109.67, 118.86, 119.73, 121.77, 124.03, 126.43, 126.92, 127.70, 127.73, 128.29, 128.55, 129.45, 134.43, 136.44, 136.64, 139.54, 140.92, 142.10; IR (KBr, cm^−1^) ν: 3026, 2239, 2206, 1605, 1556, 1512, 1437, 1400, 1335, 1080, 1070, 967, 817, 764, 742, 734, 693; EIMS (70 eV) *m*/*z* (% relative intensity): 451 (9) [M]^+^, 434 (4), 405 (4), 334 (15), 117 (22), 115 (13), 105 (6), 91 (100); HRMS–EI (70 eV, *m*/*z*): [M]^+^ calcd for C_28_H_25_N_3_O_3_, 451.1896; found, 451.1890.

#### Cyclization of 5-nitroindol-4-ylacetonitriles to pyrrolo[3,2-*e*]indoles – Typical procedure

To a solution of indole derivative **5** (1 mmol) and DBU (0.75 g, 5 mmol) in DMF (5 mL) Me_3_SiCl (0.54 g, 5 mmol) was added at room temperature. The reaction mixture was stirred for 20–30 min (TLC control), quenched with diluted HCl, extracted with EtOAc (3 × 30 mL) and dried with Na_2_SO_4_. After evaporation of the solvent the residue was purified by column chromatography (SiO_2_, hexane–EtOAc, 2:1).

**6-Benzyloxymethyl-1-cyano-3-hydroxy-7-methyl-3,6-dihydropyrrolo[3,2-*e*]indole-2-carboxylic acid ethyl ester (6a)**. Yellow crystals; mp 119–121 °C; ^1^H NMR (500 MHz, DMSO-*d*_6_) δ 1.37 (t, *J* = 7.1 Hz, 3H), 2.58 (s, 3H), 4.41 (q, *J* = 7.1 Hz, 2H), 4.48 (s, 2H), 5.77 (s, 2H), 6.84 (m, 1H), 7.23–7.36 (m, 5H), 7.73 (d, *J* = 9.1 Hz, 1H), 11.22 (br s, 1H); ^13^C NMR (125 MHz, CD_3_COCD_3_) δ 12.64, 14.44, 62.30, 70.32, 73.18, 85.95, 100.46, 104.26, 112.46, 116.13, 116.59, 119.80, 126.68, 128.37, 128.41, 128.45, 129.12, 131.11, 134.06, 138.27, 138.73,159.36; IR (KBr, cm^−1^) ν: 2987, 2212, 1753, 1682, 1618, 1597, 1499, 1453, 1430, 1368, 1333, 1321, 1265, 1136, 1062, 1028, 775; EIMS (70 eV) *m*/*z* (% relative intensity): 403 (38) [M]^+∙^, 387 (13), 311 (11), 297 (14), 283 (13), 281 (6), 192 (5), 92 (8), 91 (100); HRMS–EI (70 eV, *m*/*z*): [M]^+^ calcd for C_23_H_21_N_3_O_4_, 403.1532; found, 403.1519.

**2-Acetyl-6-benzyloxymethyl-3-hydroxy-7-methyl-3,6-dihydropyrrolo[3,2-*e*]indole-1-carbonitrile (6b)**. Yellow crystals; mp 194–196 °C; ^1^H NMR (500 MHz, DMSO-*d*_6_) δ 2.53 (d, *J* = 0.7 Hz, 3H), 2.74 (s, 3H), 4.85 (s, 2H), 5.74 (s, 2H), 6.75 (br s, 1H), 7.24–7.29 (m, 3H), 7.30–7.34 (m, 2H), 7.35 (d, *J* = 9.1 Hz, 1H), 7.76 (d, *J* = 9.1 Hz, 1H), 12.49 (br s, 1H); ^13^C NMR (125 MHz, DMSO-*d*_6_) δ 12.23, 29.97, 69.15, 72.20, 82.41, 99.30, 103.81, 112.04, 115.60, 116.54, 118.09, 127.44, 127.59, 128.27, 129.90, 132.66, 134.36, 137.32, 137.53, 187.55; IR (KBr, cm^−1^) ν: 2917, 2216, 1645, 1594, 1545, 1510, 1483, 1444, 1426, 1366, 1314, 1236, 1121, 1088, 1020, 990; EIMS (70 eV) *m*/*z* (% relative intensity): 373 (9) [M]^+∙^, 358 (9), 357 (39), 327 (15), 326 (5), 252 (6), 251 (23), 250 (8), 237 (6), 236 (7), 208 (8), 194 (7), 108 (5), 106 (5), 92 (8), 91 (100); HRMS–EI (70 eV, *m*/*z*): [M]^+^ calcd for C_22_H_19_N_3_O_3_, 373.1426; found, 373.1421.

**6-Benzyloxymethyl-2-(2,2-dimethylpropionyl)-3-hydroxy-7-methyl-3,6-dihydropyrrolo[3,2-*e*]indole-1-carbonitrile (6c)**. Brown solid; mp 155–157 °C; ^1^H NMR (500 MHz, DMSO-*d*_6_) δ 1.32 (s, 9H), 2.52 (d, *J* = 0.8 Hz, 3H), 4.47 (s, 2H), 5.73 (s, 2H), 6.68 (br s, 1H), 7.24–7.35 (m, 6H), 7.67 (d, *J* = 9.0 Hz, 1H), 12.50 (br s, 1H); ^13^C NMR (125 MHz, DMSO-*d*_6_) δ 12.27, 26.00, 44.68, 69.08, 72.21, 78.41, 98.67, 103.64, 109.66, 114.83, 116.34, 118.20, 127.43, 127.59, 128.00, 128.28, 132.87, 137.25, 137.30, 137.58, 202.17; IR (KBr, cm^−1^) ν: 2969, 2244, 1707, 1562, 1519, 1477, 1398, 1340, 1071, 1029; EIMS (70 eV) *m*/*z* (% relative intensity): 415 (9) [M]^+∙^, 400 (15), 399 (59), 369 (6), 342 (7), 313 (5), 312 (17), 294 (5), 293 (19), 292 (8), 285 (7), 284 (8), 236 (8), 208 (5), 194 (6), 193 (5), 108 (6), 92 (8), 91 (100); HRMS–EI (70 eV, *m*/*z*): [M]^+^ calcd for C_25_H_25_N_3_O_3_, 415.1896; found, 415.1878.

**2-Benzoyl-6-benzyloxymethyl-7-methyl-3-hydroxy-3,6-dihydropyrrolo[3,2-*e*]indole-1-carbonitrile (6d)**. Red crystals; mp 130–132 °C; ^1^H NMR (500 MHz, CDCl_3_) δ 2.51 (s, 3H), 4.44 (s, 2H), 5.60 (s, 2H), 6.93 (s, 1H), 7.24–7.28 (m, 2H), 7.29–7.39 (m, 4H), 7.53 (d, *J* = 9.1 Hz, 1H), 7.58–7.63 (m, 2H), 7.69–7.75 (m, 1H), 7.97–8.02 (m, 2H), 12.56 (br s, 1H); ^13^C NMR (125 MHz, CDCl_3_) δ 12.67, 69.59, 72.04, 85.65, 101.26, 103.34, 113.27, 115.64, 117.69, 119.00, 126.39, 127.74, 128.08, 128.56, 128.69, 128.87, 129.95, 132.94, 134.15, 135.87, 136.85, 137.15, 189.1; IR (KBr, cm^−1^) ν: 2921, 2215, 1639, 1599, 1569, 1496, 1479, 1424, 1367, 1329, 1314, 1260, 1085, 1072, 778, 732, 693; ESIMS (MeOH) *m*/*z*: 436 [M + H]^+^, 458 [M + Na]^+^; HRMS–EI (70 eV, *m*/*z*): [M + Na]^+^ calcd for C_27_H_21_N_3_O_3_Na, 458.1475; found, 458.1495.

**6-Benzyloxymethyl-3-hydroxy-7-methyl-3,6-dihydropyrrolo[3,2-*e*]indole-1,2-dicarbonitrile (6e)**. Brownish crystals; mp 150–152 °C; ^1^H NMR (500 MHz, DMSO-*d*_6_) δ 2.53 (s, 3H), 4.47 (s, 2H), 5.75 (s, 2H), 6.70 (br s, 1H), 7.22–7.34 (m, 5H), 7.36 (d, *J* = 9.1 Hz, 1H), 7.83 (d, *J* = 9.1 Hz, 1H), 13.29 (br s, 1H); ^13^C NMR (125 MHz, DMSO-*d*_6_) δ 12.26, 69.22, 72.30, 99.16, 103.44, 108.90, 110.30, 112.65, 114.14, 115.21, 117.70, 127.47, 127.64, 128.31, 128.33, 129.46, 132.86, 137.52, 138.01; IR (KBr, cm^−1^) ν: 2922, 2224, 1544, 1484, 1393, 1351, 1319, 1209, 1145, 1064, 1024, 774, 753, 699; ESIMS (MeOH/CH_2_Cl_2_) *m*/*z*: 357 [M + H]^+^, 379 [M + Na]^+^, 735 [2M + Na]^+^.

**6-Benzyloxymethyl-1-cyano-3-hydroxy-7-methyl-3,6-dihydropyrrolo[3,2-*e*]indole-2-carboxylic acid dimethylamide (6f)**. Brown semisolid; ^1^H NMR (500 MHz, DMSO-*d*_6_) δ 2.49 (s, 3H), 3.02 (s, 3H), 3.30 (br s, 3H), 4.44 (s, 2H), 5.68 (s, 2H), 6.61 (br s, 1H), 7.22–7.33 (m, 6H), 7.52 (d, *J* = 9.0 Hz, 1H), 13.24 (br s, 1H); ^13^C NMR (125 MHz, DMSO-*d*_6_) δ 12.21, 34.38, 37.24, 68.92, 72.06, 98.73, 104.84, 107.15, 115.29, 117.24, 117.99, 127.35, 127.49, 127.93, 128.22, 132.58, 135.89, 137.64 (3 peaks not visible); IR (KBr, cm^−1^) ν: 2930, 2208, 1615, 1525, 1445, 1390, 1363, 1319, 1258, 1209, 1121, 1066, 778, 738, 698; EIMS (70 eV) *m*/*z* (% relative intensity): 402 (10) [M]^+∙^, 387 (7), 386 (27), 312 (6), 311 (17), 280 (5), 265 (6), 221 (5), 220 (6), 213 (6), 193 (6), 192 (5), 165 (14), 135 (7), 108 (39), 107 (10), 105 (7), 92 (10), 91 (100); HRMS–EI (70 eV, *m*/*z*): [M]^+^ calcd for C_23_H_22_N_4_O_3_, 402.1692; found, 402.1684.

**6-Benzyloxymethyl-3-hydroxy-7-methyl-2-((*E*)-styryl)-3,6-dihydropyrrolo[3,2-*e*]indole-1-carbonitrile (6g)**. Dark green solid; mp 130–132 °C; ^1^H NMR (500 MHz, DMSO-*d*_6_) δ 2.52 (d, *J* = 0.8 Hz, 3H), 4.48 (s, 2H), 5.71 (s, 2H), 6.69 (br s, 1H), 7.21–7.40 (m, 8H), 7.44–7.48 (m, 2H), 7.56 (d, *J* = 9.0 Hz, 1H), 7.69 (m, 2H), 7.77 (d, *J* = 16.5 Hz, 1H), 12.10 (s, 1H); ^13^C NMR (125 MHz, DMSO-*d*_6_) δ 12.26, 69.07, 72.18, 98.74, 103.21, 108.35, 113.73, 115.56, 117.83, 117.85, 126.83, 127.42, 127.56, 127.66, 128.27, 128.60, 128.91, 129.05, 132.84, 133.04, 135.95, 136.87, 136.99, 137.63; IR (KBr, cm^−1^) ν: 2923, 2200, 1739, 1547, 1467, 1389, 1361, 1339, 1317, 1203, 1142, 1066, 955, 746; EIMS (70 eV) *m*/*z* (% relative intensity): 433 (2) [M]^+∙^, 431 (16), 325 (10), 297 (11), 165 (28), 108 (92), 107 (68), 106 (35), 105 (46), 92 (8), 91 (82); HRMS–ESI (*m*/*z*): [M + Na]^+^ calcd for C_28_H_23_N_3_O_2_Na, 456.1688; found, 456.1685.

**6-Benzyloxymethyl-1-cyano-7-methyl-3,6-dihydropyrrolo[3,2-*e*]indole-2-carboxylic acid ethyl ester (8a)**. Pale creamy crystals; mp 215–217 °C; ^1^H NMR (500 MHz, DMSO-*d*_6_) δ 1.40 (t, *J* = 7.1 Hz, 3H), 2.52 (s, 3H), 4.44 (q, *J* = 7.1 Hz, 2H), 4.47 (s, 2H), 5.71 (s, 2H), 6.74 (br s, 1H), 7.24–7.35 (m, 6H), 12.99 (br s, 1H); ^13^C NMR (125 MHz, DMSO-*d*_6_) δ 12.23, 14.07, 61.35, 69.09, 72.18, 87.59, 99.08, 106.70, 111.12, 115.93, 118.34, 119.90, 127.42, 127.57, 128.27, 129.34, 131.58, 132.28, 136.77, 137.57, 159.10; IR (KBr, cm^−1^) ν: 3264, 2219, 1689, 1527, 1455, 1434, 1362, 1315, 1261, 1064, 1055, 1025, 743; EIMS (70 eV) *m*/*z* (% relative intensity): 387 (50) [M]^+∙^, 312 (8), 311 (37), 281 (14), 280 (5), 266 (5), 235 (6), 220 (12), 206 (5), 192 (6), 165 (5), 92 (8), 91 (100); HRMS–EI (*m*/*z*): [M]^+^ calcd for C_23_H_21_N_3_O_3_, 387.1583; found, 387.1587.

**2-Benzoyl-6-benzyloxymethyl-7-methyl-3,6-dihydropyrrolo[3,2-*e*]indole-1-carbonitrile (8d)**. Yellow crystals; mp 180–182 ºC; ^1^H NMR (500 MHz, DMSO-*d*_6_) δ 2.53 (s, 3H), 4.47 (s, 2H), 5.74 (s, 2H), 6.75 (s, 1H), 7.23–7.38 (m, 6H), 7.60–7.67 (m, 2H), 7.69–7.78 (m, 2H), 7.89–7.94 (m, 2H), 12.93 (br s, 1H); ^13^C NMR (125 MHz, DMSO-*d*_6_) δ 12.24, 69.07, 72.17, 88.59, 99.28, 106.82, 111.83, 116.11, 118.42, 120.51, 127.44, 127.59, 128.28, 128.67, 129.38, 132.20, 132.38, 133.11, 136.36, 136.86, 136.97, 137.57, 185.27; IR (KBr, cm^−1^) ν: 3265, 2217, 1710, 1624, 1540, 1510, 1408, 1329, 1254, 1062, 942, 739, 696; ESIMS (MeOH) *m*/*z*: 420 [M + H]^+^, 442 [M + Na]^+^, 861 [2M + Na]^+^; HRMS–ESI (*m*/*z*): [M + Na]^+^ calcd for C_27_H_21_N_3_O_2_Na, 442.1526; found, 442.1544.

#### Debenzyloxymethylation of compound **8a**

Compound **8** (100 mg, 0.26 mmol), ammonium formate (0.16 g, 10 mmol) and 10% palladium on charcoal (90 mg) were suspended in isopropanol (5 mL), flushed with argon for 5 min and then heated in a sealed tube at 95 °C overnight. Then the reaction mixture was passed through Celite, washed with dichloromethane–methanol, 1:1 (15 mL). After evaporation the residue was purified by column chromatography on silica gel with hexane–ethyl acetate (gradient 4:1 to 1:1). The following compounds were obtained:

**1-Cyano-7-methyl-3,6-dihydropyrrolo[3,2-*e*]indole-2-carboxylic acid ethyl ester (9a)**. Yield 22%; mp > 280 °C; ^1^H NMR (500 MHz, DMSO-*d*_6_) δ 1.39 (t, *J* = 7.1 Hz, 3H), 2.45 (s, 3H), 4.42 (q, *J* = 7.1 Hz, 2H), 6.56 (s, 1H), 7.19 (d, *J* = 8.8 Hz, 1H), 7.40 (d, *J* = 8.8 Hz, 1H), 11.33 (s, 1H), 12.85 (s, 1H); ^13^C NMR (125 MHz, DMSO-*d*_6_) δ 13.27, 14.07, 61.19, 87.56, 97.30, 105.78, 112.34, 116.06, 118.75, 120.01, 128.49, 131.03, 131.09, 135.00, 159.14; IR (film, cm^−1^) ν: 3265, 2256, 1690, 1549, 1526, 1439, 1363, 1338, 1254, 1115, 1073,1016; EIMS (70 eV) *m*/*z* (% relative intensity): 267 (75) [M]^+∙^, 222 (26), 221 (100), 194 (25), 193 (55), 167 (17).

**1,7-Dimethyl-3,6-dihydropyrrolo[3,2-*e*]indole-2-carboxylic acid ethyl ester (10a)**. Yield 34%; mp 225–227 °C dec; ^1^H NMR (500 MHz, DMSO-*d*_6_) δ 1.35 (t, *J* = 7.0 Hz, 3H), 2.42 (s, 3H), 2.72 (s, 3H), 4.32 (q, *J* = 7.0 Hz, 2H), 6.46 (s, 1H), 7.04 (d, *J* = 8.8 Hz, 1H), 7.23 (d, *J* = 8.8 Hz, 1H), 10.97 (s, 1H), 11.21 (s, 1H); ^13^C NMR (125 MHz, DMSO-*d*_6_) δ 11.57, 13.35, 14.40, 59.47, 98.24, 105.38, 110.99, 118.48, 119.62, 120.46, 120.54, 129.65, 131.77, 133.23, 162.01; IR (KBr, cm^−1^) ν: 3318, 1675, 1548, 1534, 1437, 1362, 1334, 1291, 1243, 1209, 1190, 1117, 1092, 1017, 772.

## Supporting Information

File 1 ^1^H and ^13^C NMR, IR and mass spectra for compounds **4**, **5a**–**g**, **6a**–**g**, **8a**, **8d, 9a** and **10a**.
