# Supplementary material for: Simple synthesis of pyrrolo[3,2-e]indole-1-carbonitriles
Source: Beilstein J Org Chem. 2013 May 15;9:934–41. doi: 10.3762/bjoc.9.107 (PMC3678543; doi:10.3762/bjoc.9.107)
Supplement: File 1 — 1H and 13C NMR, IR and mass spectra for compounds 4, 5a–g, 6a–g, 8a, 8d, 9a and 10a. [file Beilstein_J_Org_Chem-09-934-s001.pdf]

Supporting Information

for

Simple synthesis of pyrrolo[3,2-*e*]indole-1-carbonitriles

Adam Trawczyński[1,2], Robert Bujok[1], Zbigniew Wróbel[1], Krzysztof Wojciechowski[1]

[1]Institute of Organic Chemistry, Polish Academy of Sciences, ul. Kasprzaka 44/52, POBox 58, 01-224 Warszawa, Poland, Fax: +48 (22) 632 66 81 and [2]Department of Chemistry, Warsaw University of Technology, ul. Noakowskiego 3, 00-664 Warszawa, Poland

E-mail: Krzysztof Wojciechowski - krzysztof.wojciechowski@icho.edu.pl

*Corresponding author

^{1}H and ^{13}C NMR, IR and mass spectra for compounds 4, 5a–g, 6a–g, 8a, 8d, 9a and 10a

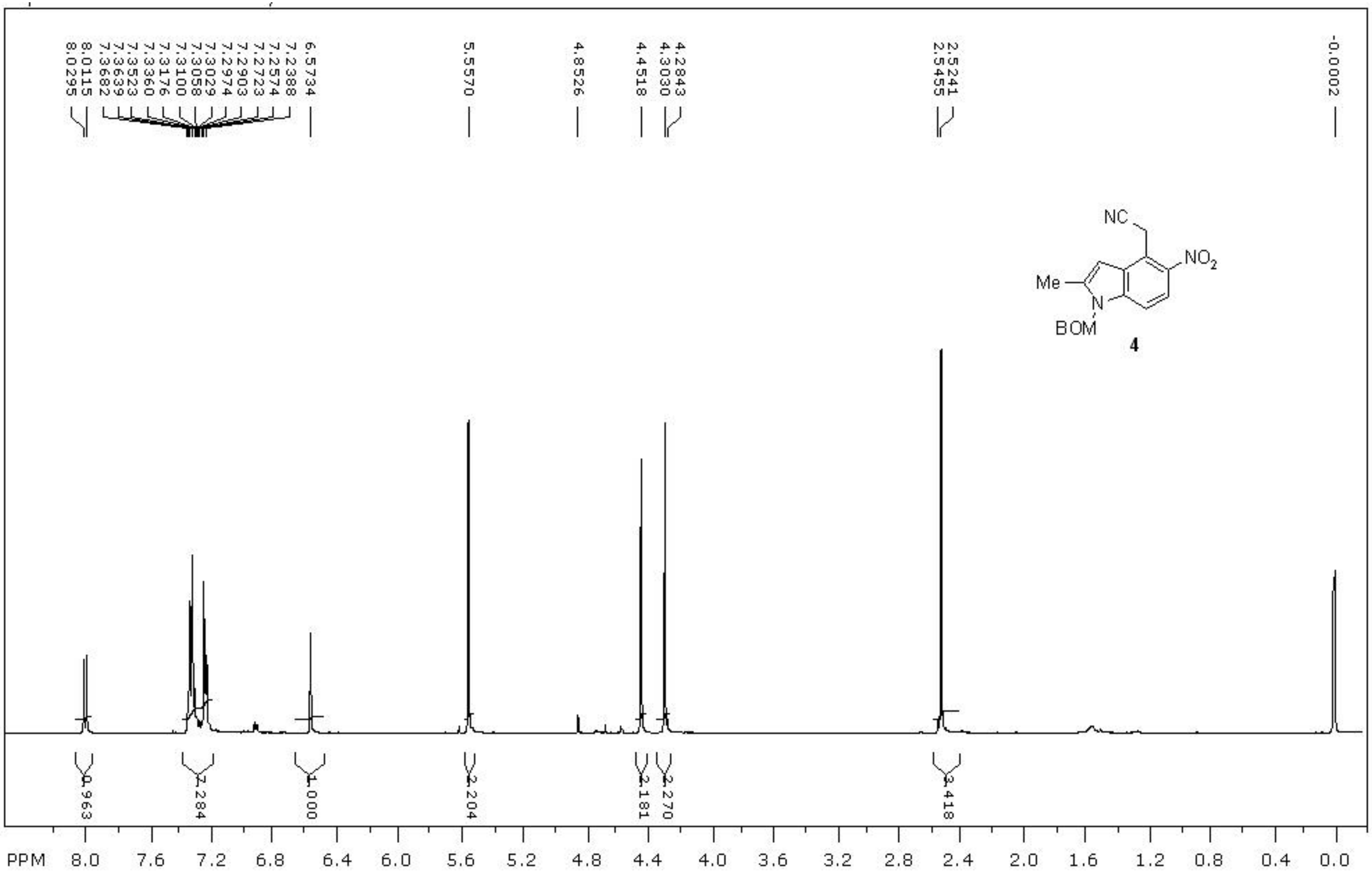

file: ...LEVAR500\ATIN-9\ATIN-9-H1.fid\fid block # 1 expt: "s2pul"
transmitter freq.: 499.833542 MHz
time domain size: 32768 points
width: 5506.61 Hz = 11.0169 ppm = 0.168048 Hz/pt
number of scans: 32

freq. of 0 ppm: 499.831293 MHz
processed size: 32768 complex points
LB: 1.061 GF: 0.0000
Hz/cm: 174.554 ppm/cm: 0.34922

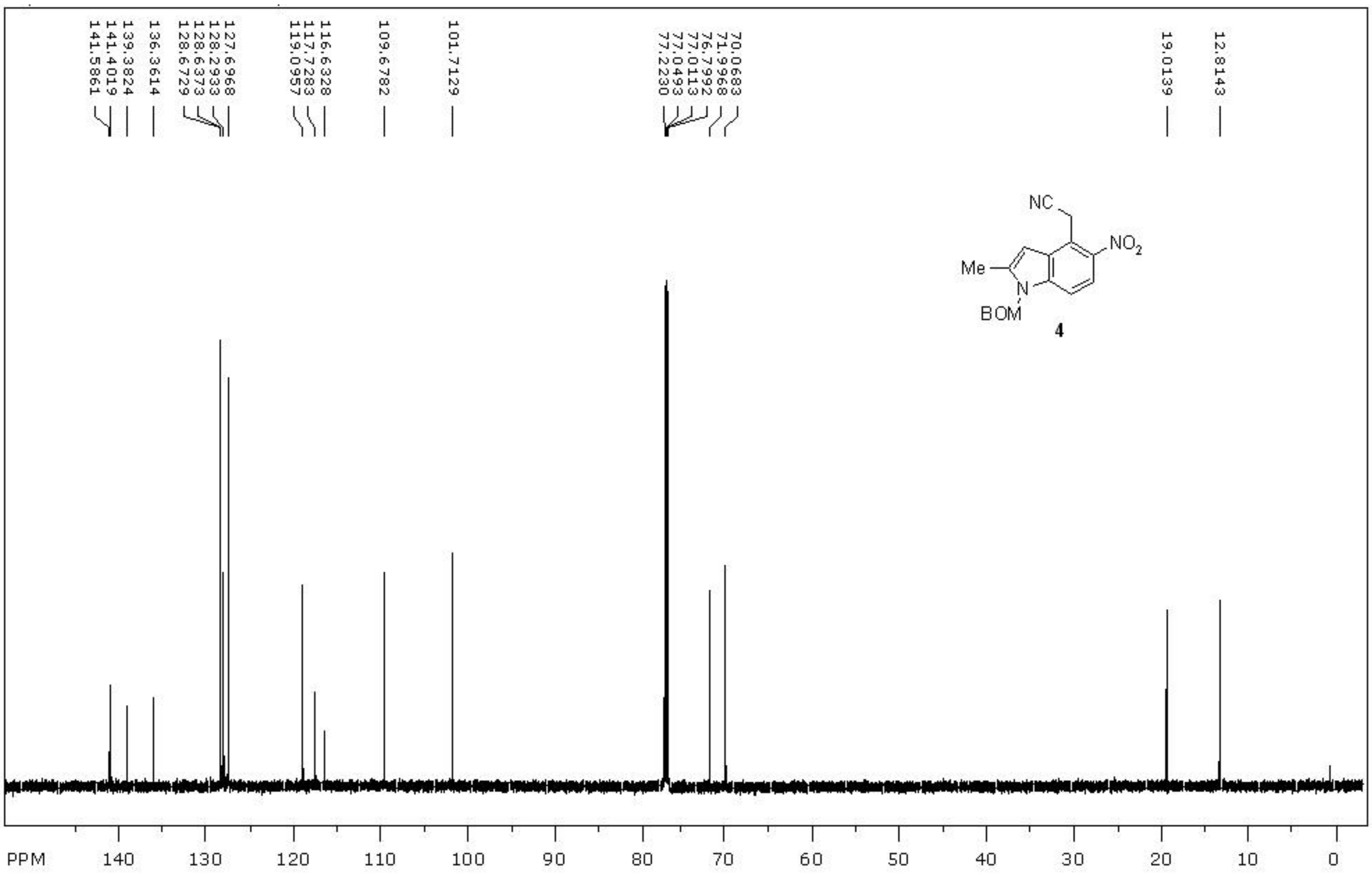

file: ...LOINDOLE\ATIN-5\ATIN-5-C13.fid\fid block # 1 expt: "s2pul"
transmitter freq.: 150.845241 MHz
time domain size: 90910 points
width: 37878.79 Hz = 251.1103 ppm = 0.416663 Hz/pt
number of scans: 1088

freq. of 0 ppm: 150.828649 MHz
processed size: 131072 complex points
LB: 0.500 GF: 0.0000
Hz/cm: 950.009 ppm/cm: 6.29791

File Name : E:\Inne\PW_Warszawa\af1066.ms2
Creation Date/Time : 11-04-07 at 13:17:22
File Type : Lo-Res Data - Ctd (Magnet)
File Source : Acquired on MASPEC II system [II32/99D9]
File Title : ATIN-5 (EI 70 eV 33-1000)
Operator : Marian Olejnik
Instrument : AMD 604
Notes : A. Trawczyński

SCAN GRAPH. Flagging=Nominal M/z. Highlighting=Base Peak.
Scan 31#3:54. Entries=663. Base M/z=91.1. 100% Int.=73.344. Temp =231.

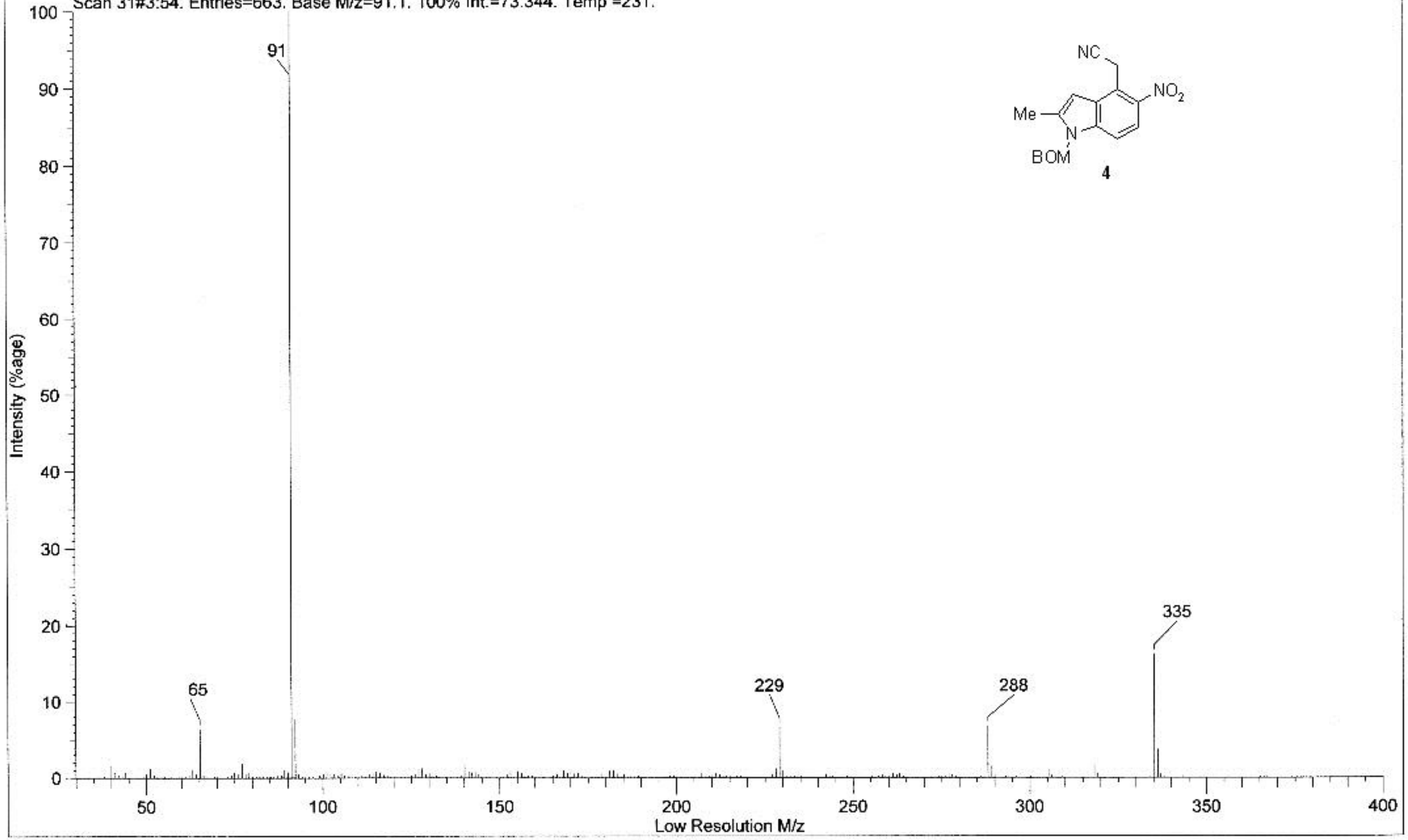

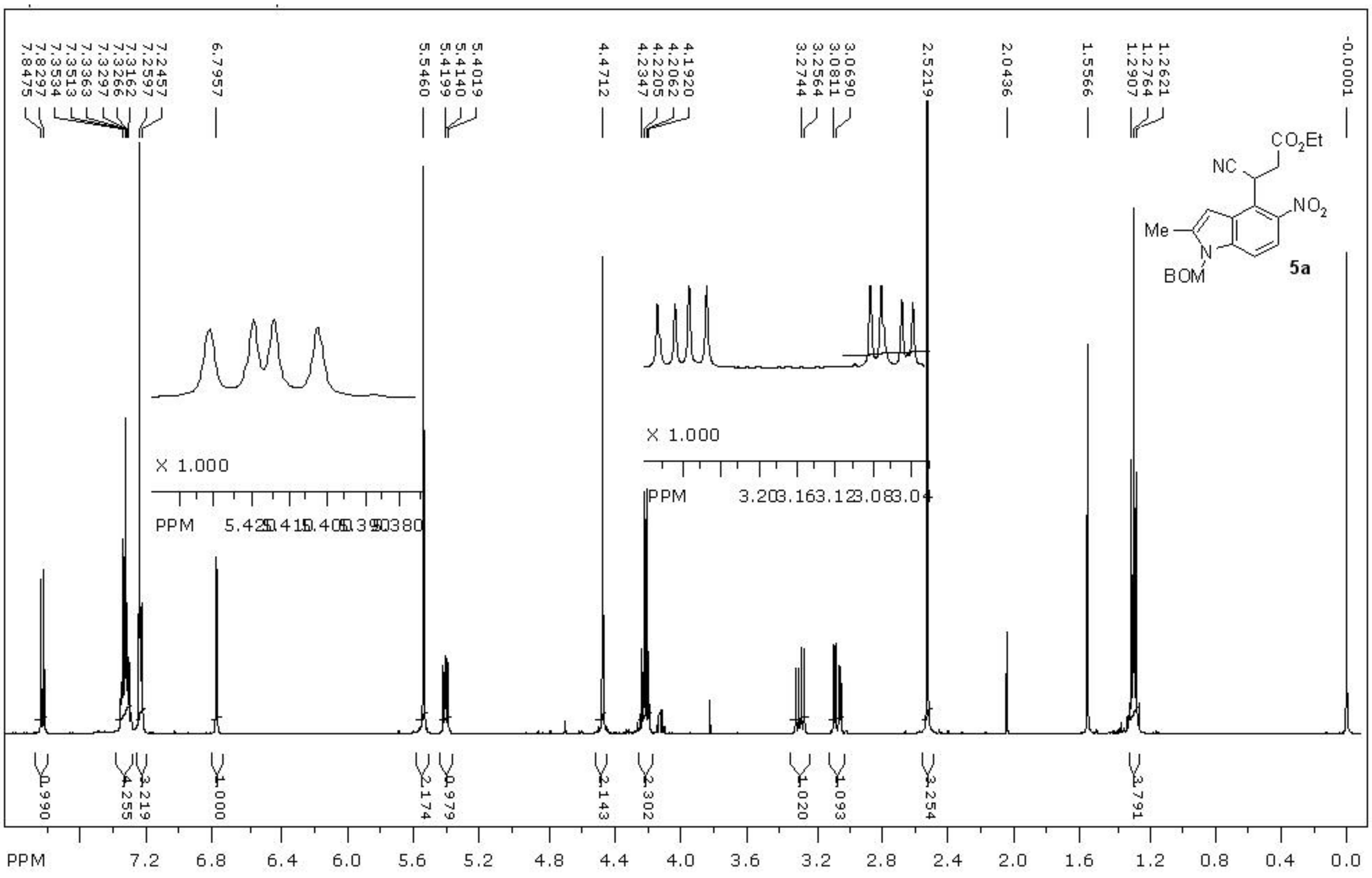

file: ...OLE\ATIN-29_K\ATIN-29_K-H1.fid\fid block# 1 expt: "s2pul"
transmitter freq.: 499.835542 MHz
time domain size: 65536 points
width: 9541.98 Hz = 19.0902 ppm = 0.145599 Hz/pt
number of scans: 32

freq. of 0 ppm: 499.831293 MHz
processed size: 65536 complex points
LB: 0.500 GF: 0.0000
Hz/cm: 163.386 ppm/cm: 0.32688

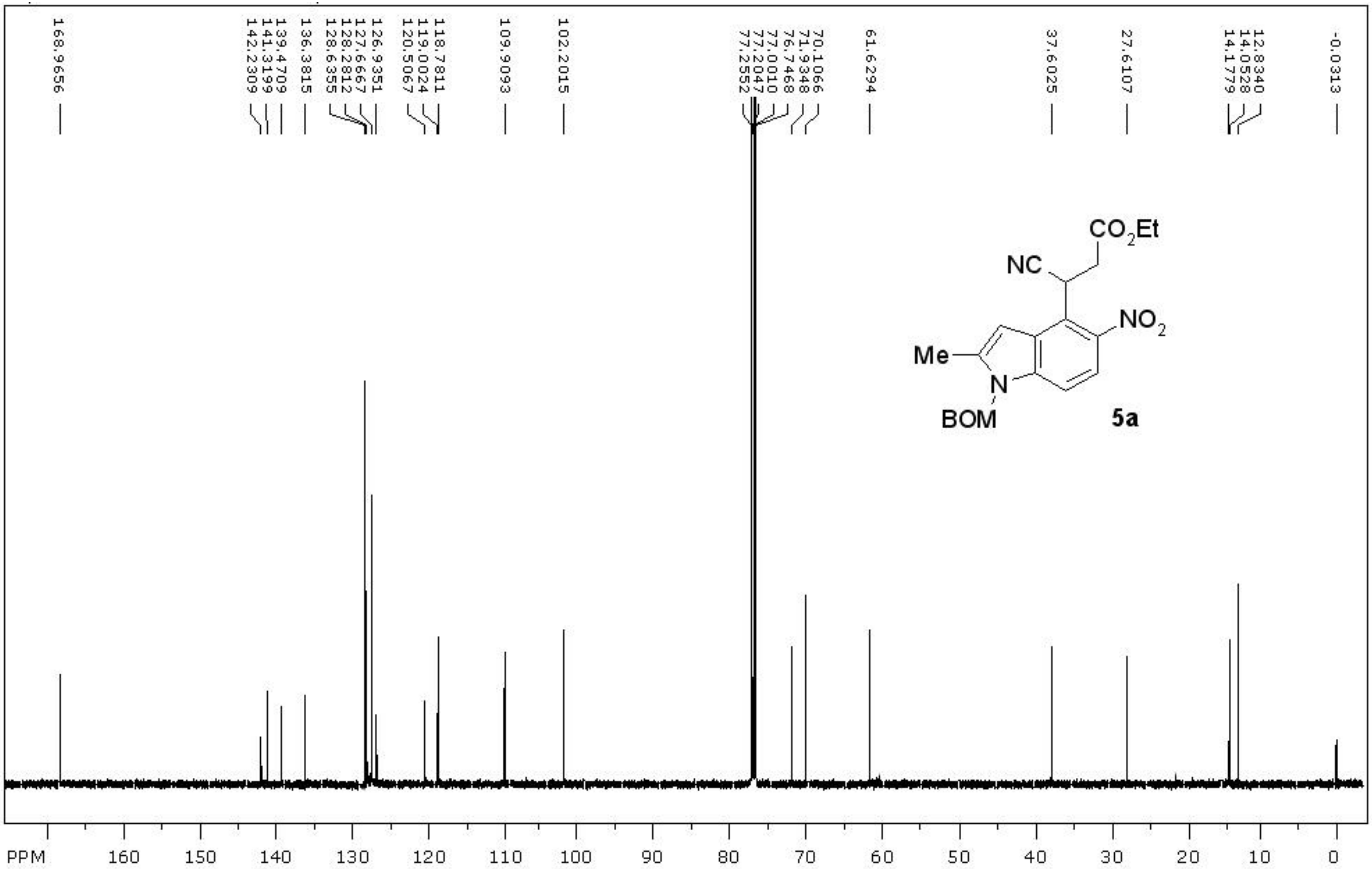

file: ...DOLE\ATIN-10K\ATIN-10K-C13.fid\fid block# 1 expt: "s2pul"
transmitter freq. : 125.696504 MHz
time domain size : 80128 points
width: 32051.28 Hz = 254.9894 ppm = 0.400001 Hz/pt
number of scans : 2128

freq. of 0 ppm: 125.682680 MHz
processed size: 131072 complex points
LB: 0.500 GF: 0.0000
Hz/cm: 904.602 ppm/cm: 7.19671

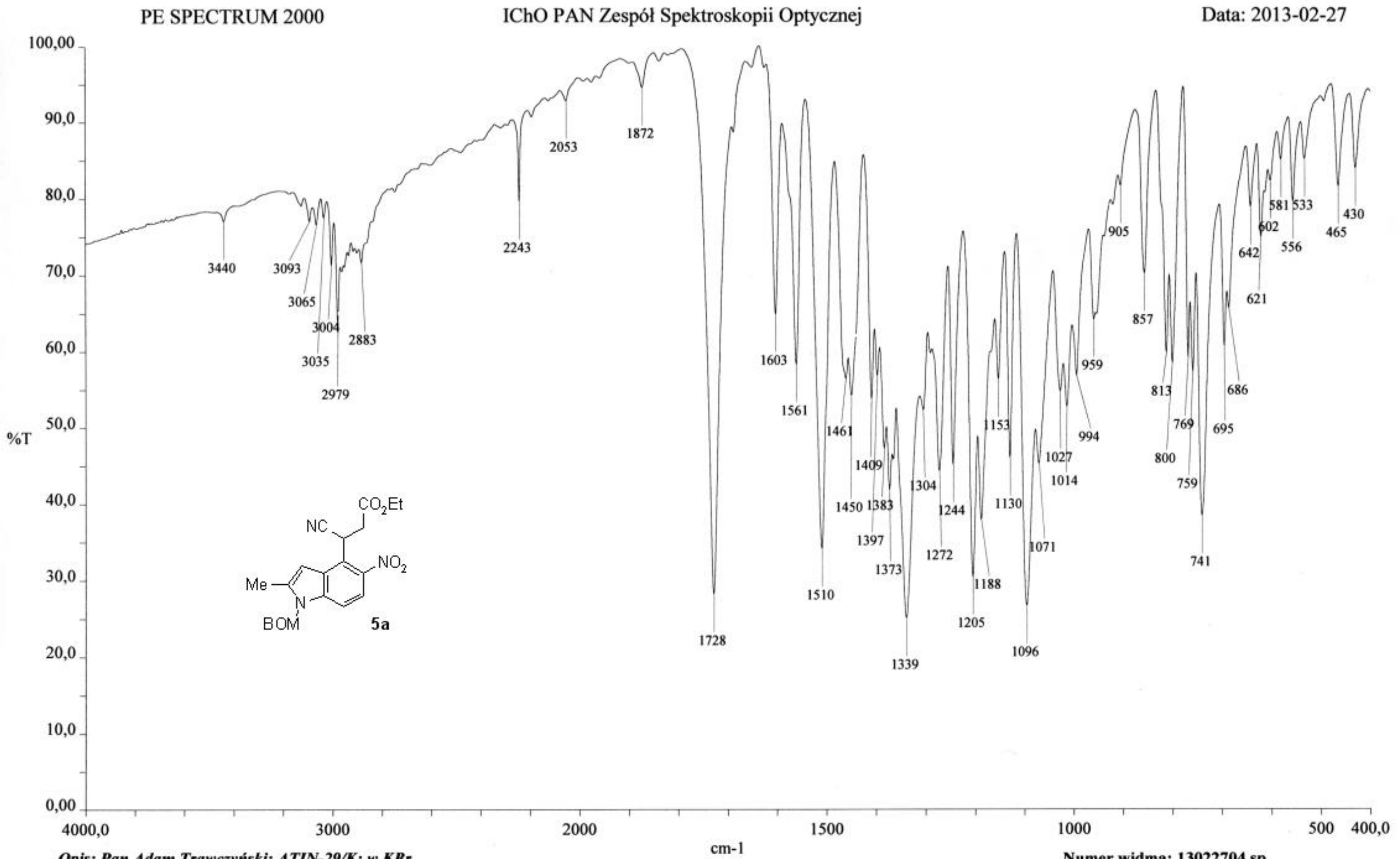

Opis: Pan Adam Trawczyński; ATIN-29/K; w KBr

Uwagi:

Numer widma: 13022704.sp

Operator: Alicja Dziedzic

File Name	:	E:\IChO\Z07_EG\at1755.ms2
Creation Date/Time	:	11-06-13 at 14:48:00
File Type	:	Lo-Res Data - Ctd (Magnet)
File Source	:	Acquired on MASPEC II system [II32/99D9]
File Title	:	ATIN-10/K (EI 70 eV 33-800)
Operator	:	Marian Olejnik
Instrument	:	AMD 604
Notes	:	A. Trawczyński

SCAN GRAPH. Flagging=Nominal M/z. Highlighting=Base Peak.
Scan 77#9:16. Entries=816. Base M/z=91.1. 100% Int.=98.56. Temp =241.

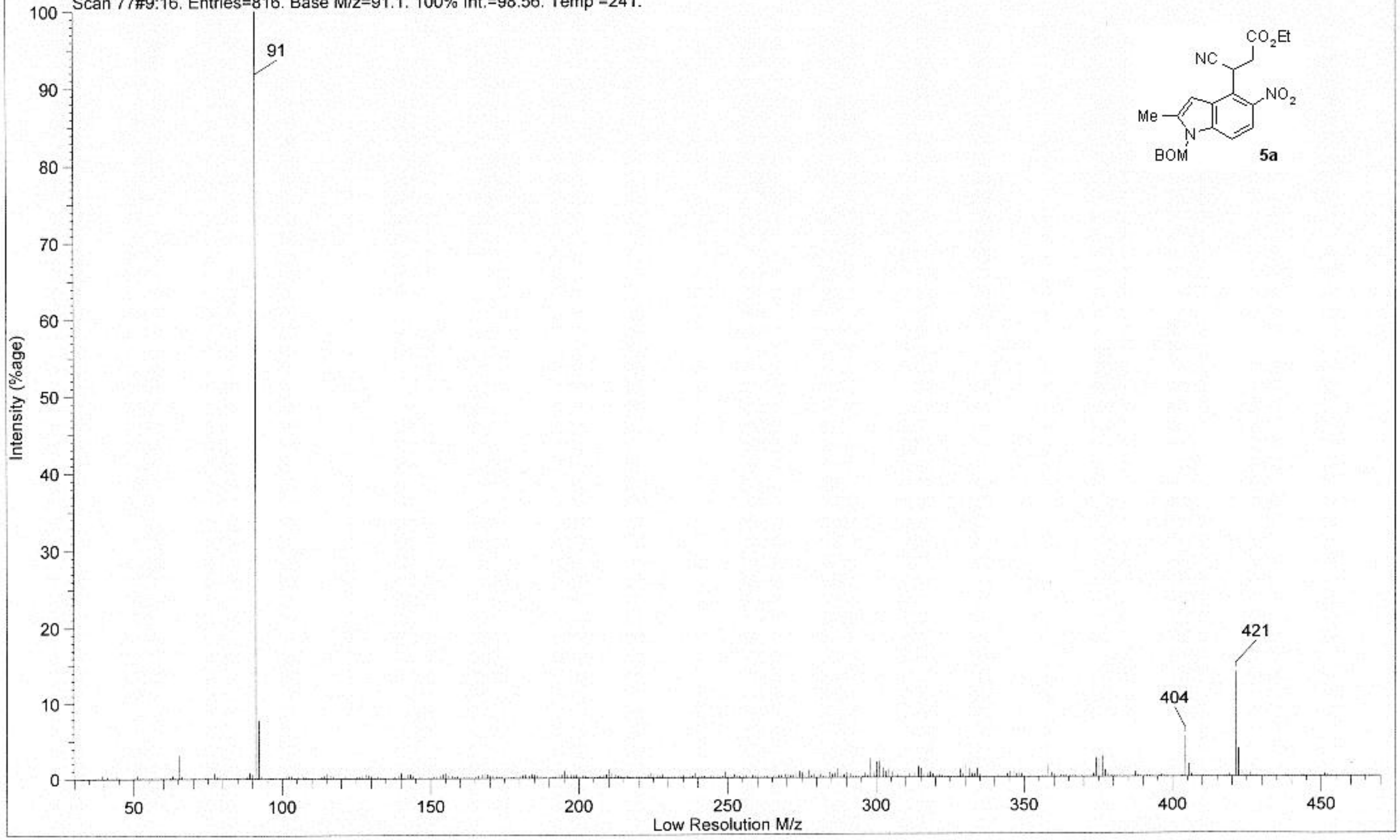

5a

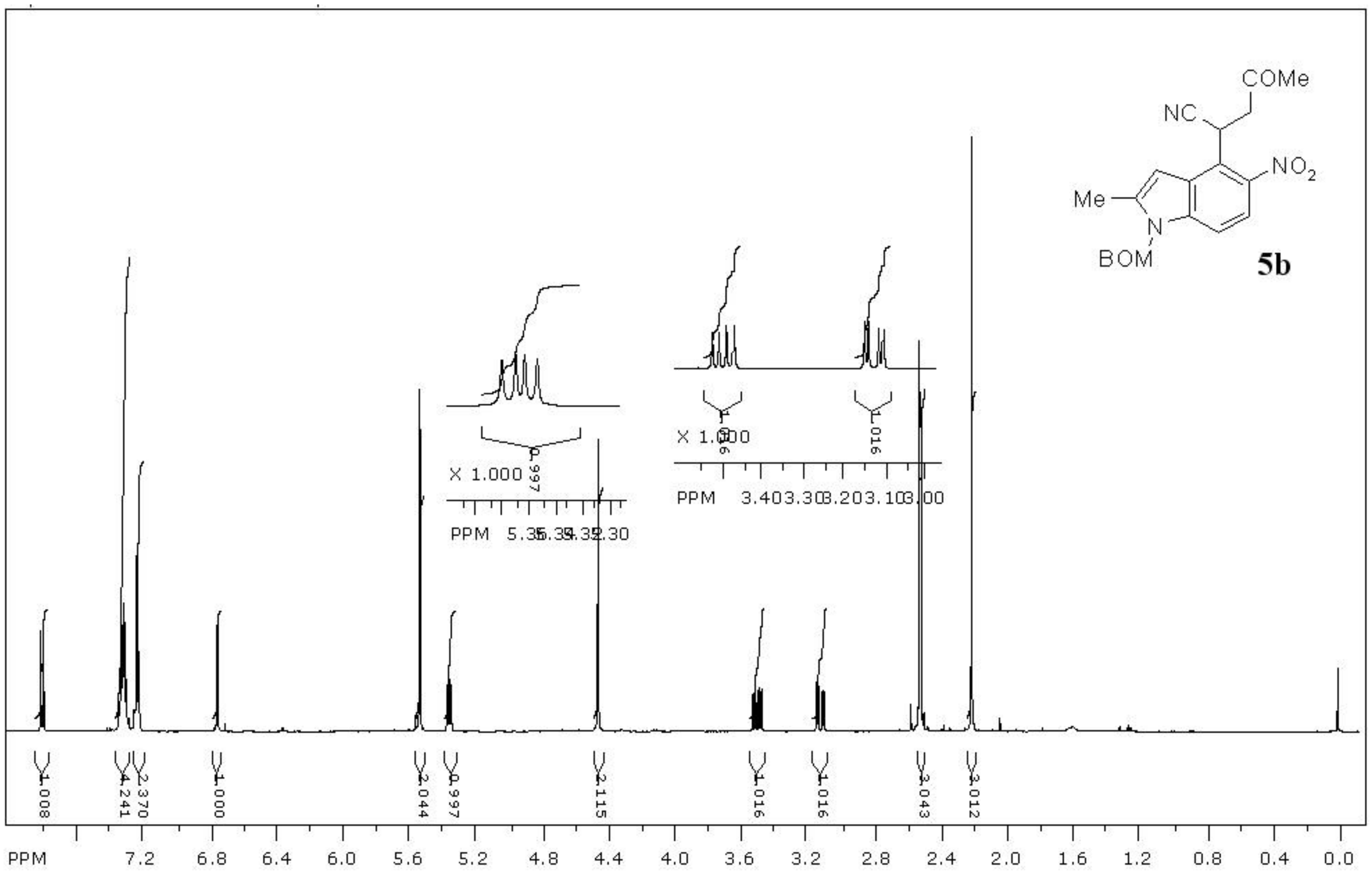

file: ... OINDOLE\ATIN-32\ATIN-32-H1.fid\fid block# 1 expt: "s2pul"
transmitter freq. : 499.834042 MHz
time domain size: 127118 points
width: 10593.22 Hz = 21.1935 ppm = 0.083334 Hz/pt
number of scans: 64

freq. of 0 ppm: 499.831293 MHz
processed size: 131072 complex points
LB: 0.500 GF: 0.0000
Hz/cm: 163.911 ppm/cm: 0.32793

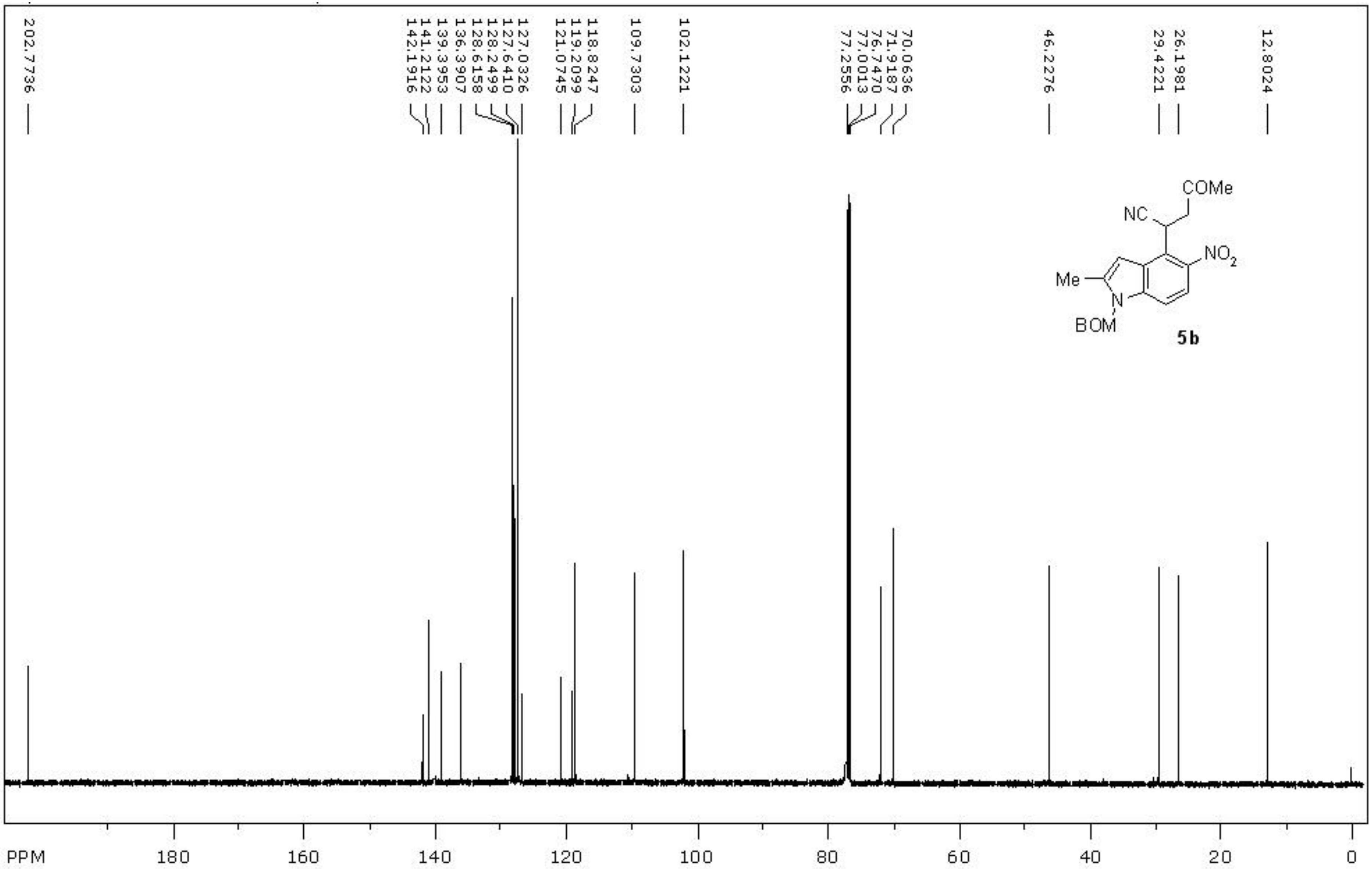

file: ...INDOLE\ATIN-32\ATIN-32-C13.fid\fid block #1 expt: "s2pul"
transmitter freq.: 125.696504 MHz
time domain size: 80128 points
width: 32051.28 Hz = 254.9894 ppm = 0.400001 Hz/pt
number of scans: 2320

freq. of 0 ppm: 125.682683 MHz
processed size: 131072 complex points
LB: 0.500 GF: 0.0000
Hz/cm: 1047.919 ppm/cm: 8.33690

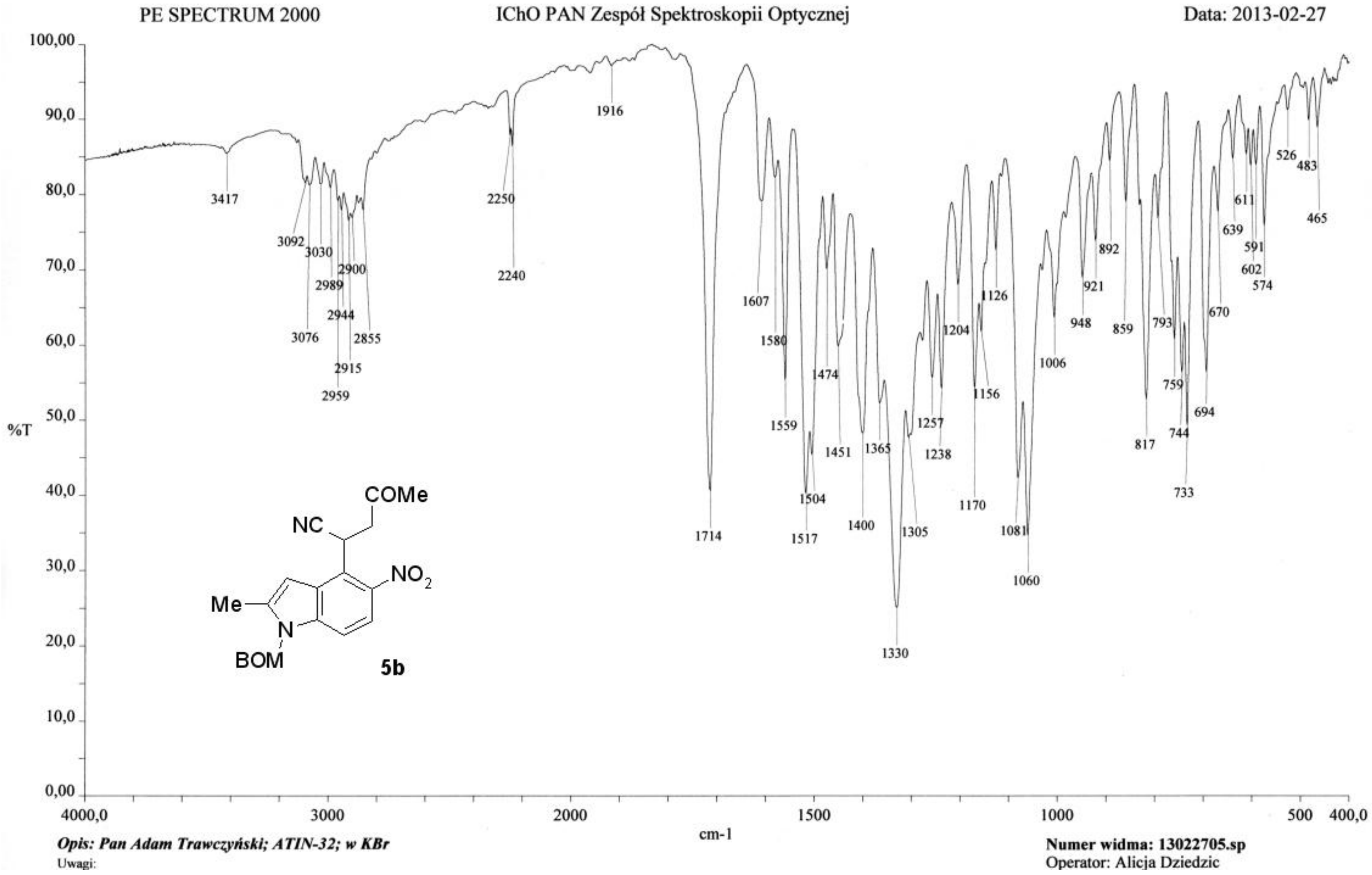
PE SPECTRUM 2000
IChO PAN Zespół Spektroskopii Optycznej
Data: 2013-02-27
%T
cm-1
3417 3092 3076 3030 2989 2959 2944 2915 2900 2855 2250 2240 1916 1714 1607 1580 1559 1517 1504 1474 1451 1400 1365 1330 1305 1257 1238 1204 1170 1156 1126 1081 1060 1006 948 921 892 859 817 793 759 744 733 694 670 639 611 602 591 574 526 483 465
COMe
NC
NO2
Me
N
BOM
5b
Opis: Pan Adam Trawczyński; ATIN-32; w KBr
Uwagi:
Numer widma: 13022705.sp
Operator: Alicja Dziedzic

File Name : E:\IChO\Z07_EG\at2985.ms2
Creation Date/Time : 11-10-10 at 14:16:27
File Type : Lo-Res Data - Ctd (Magnet)
File Source : Acquired on MASPEC II system [II32/99D9]
File Title : ATIN-32 (EI 70 eV 33-800)
Operator : Marian Olejnik
Instrument : AMD 604
Notes : A. Trawczyński

SCAN GRAPH. Flagging=Nominal M/z. Highlighting=Base Peak.
Scan 37#5:28. Entries=646. Base M/z=91.2. 100% Int.=99.584. Temp =251.

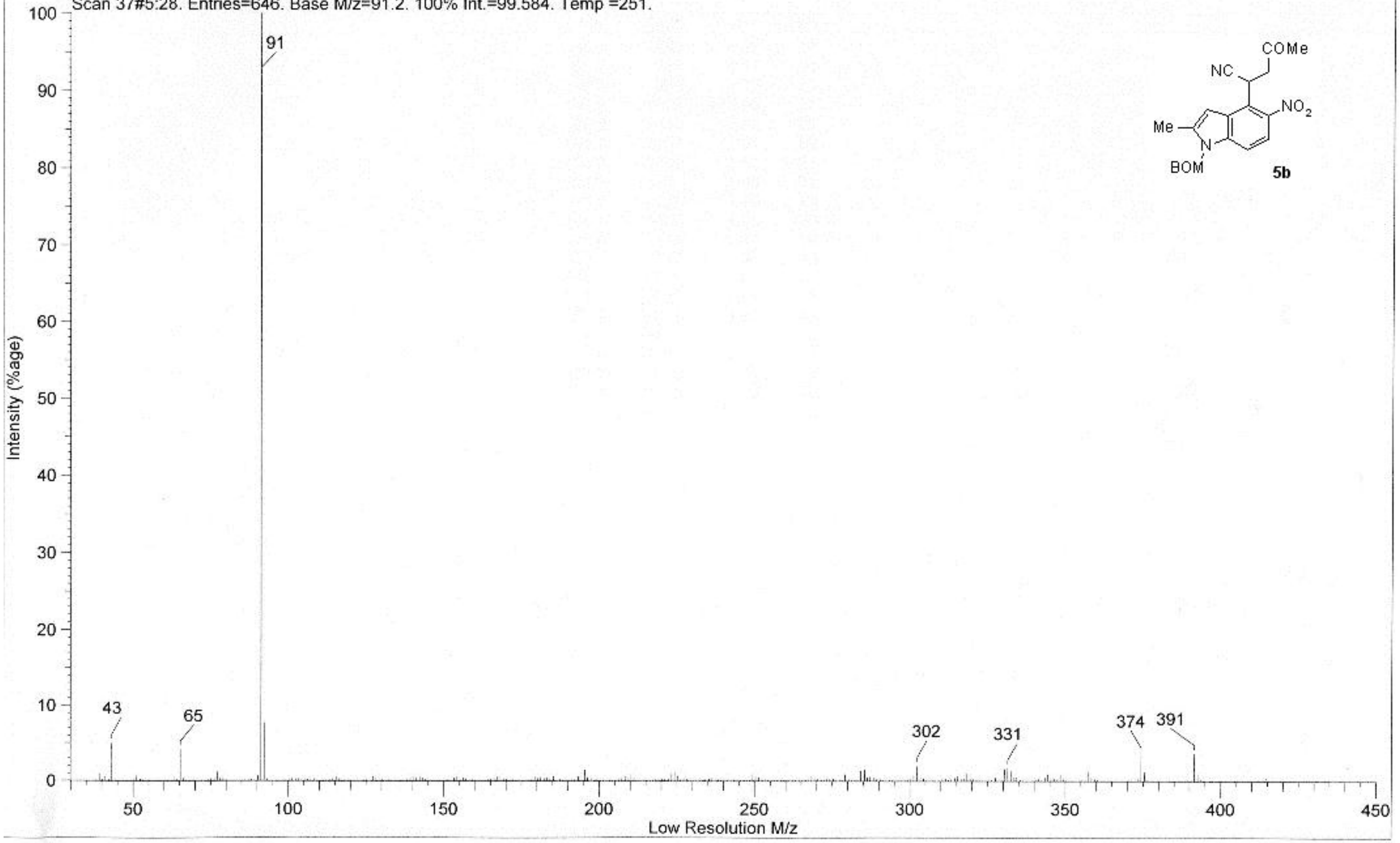

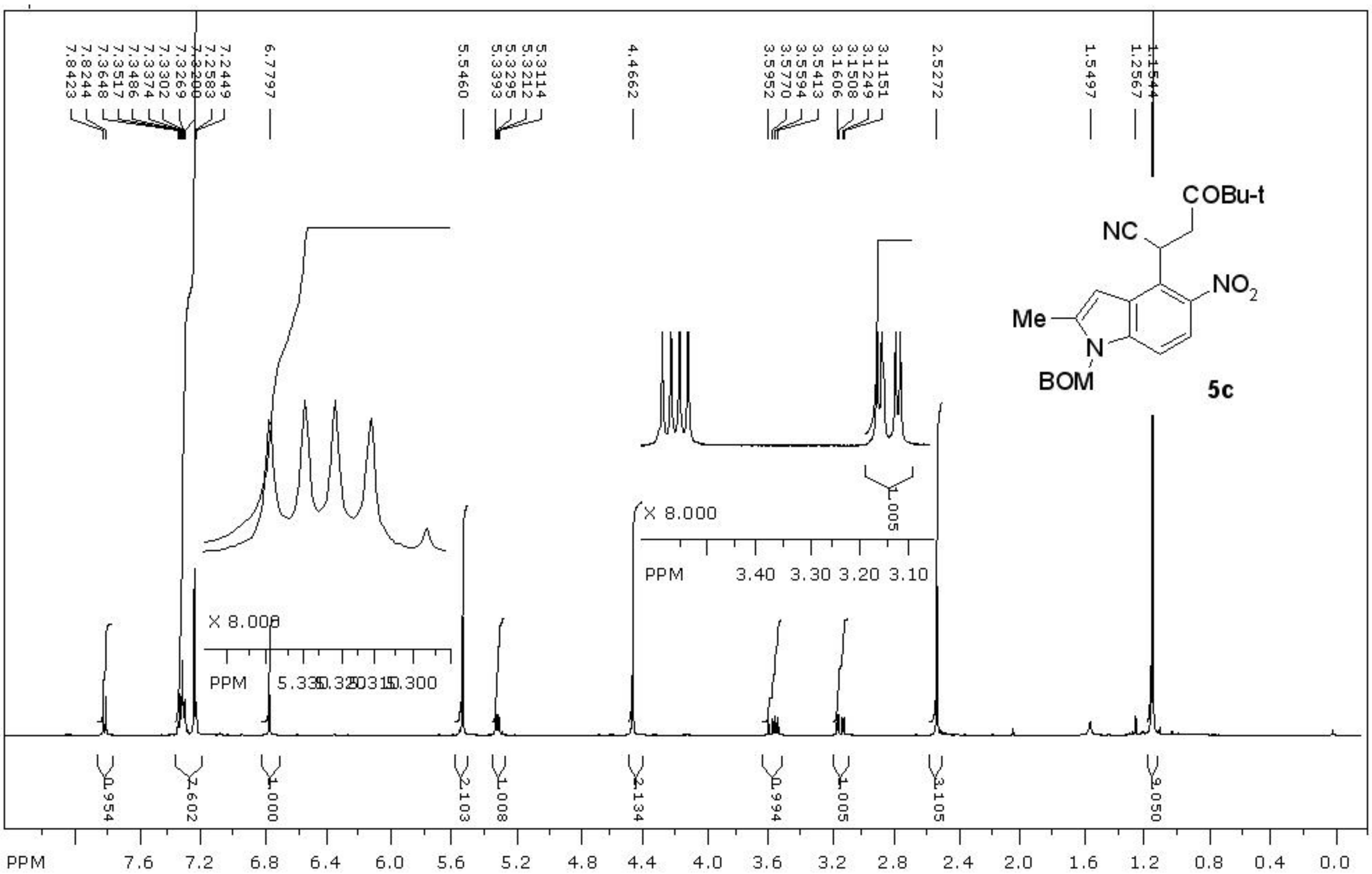

file: ...\NMR\PIROLOINDOLE\ATIN-26-PP\1\fid expt: <zg>
transmitter freq.: 500.133089 MHz
time domain size: 65536 points
width: 10330.58 Hz = 20.6557 ppm = 0.157632 Hz/pt
number of scans: 32

freq. of 0 ppm: 500.130025 MHz
processed size: 32768 complex points
LB: 0.000 GF: 0.0000
Hz/cm: 173.339 ppm/cm: 0.34659

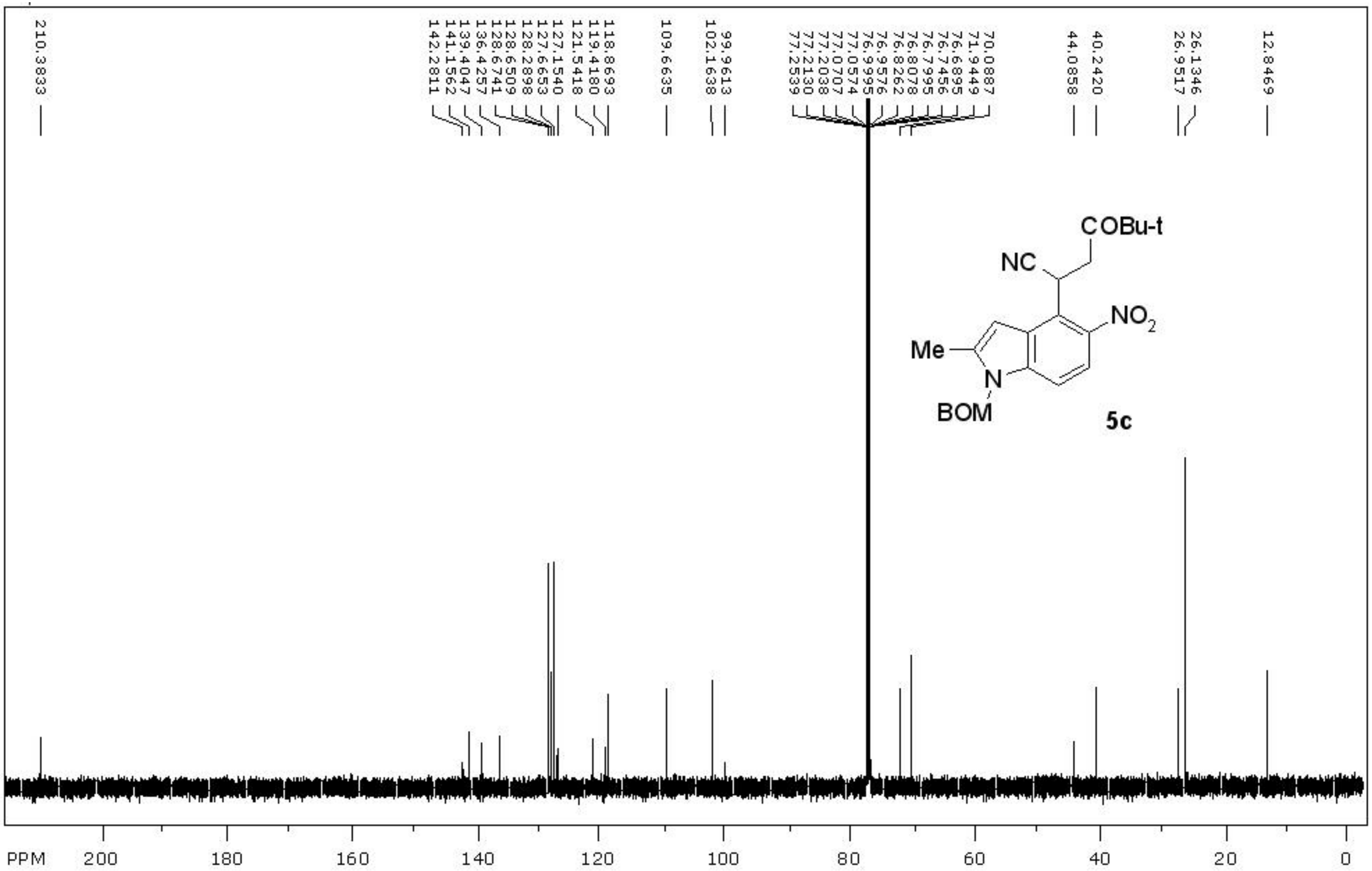

file: ...\NMR\PIROLOINDOLE\ATIN-26-PP\2\fid expt: <zgpg>
transmitter freq.: 125.770364 MHz
time domain size: 65536 points
width: 32679.74 Hz = 259.8366 ppm = 0.498653 Hz/pt
number of scans: 1271

freq. of 0 ppm: 125.757794 MHz
processed size: 262144 complex points
LB: 0.500 GF: 0.0000
Hz/cm: 1101.020 ppm/cm: 8.75421

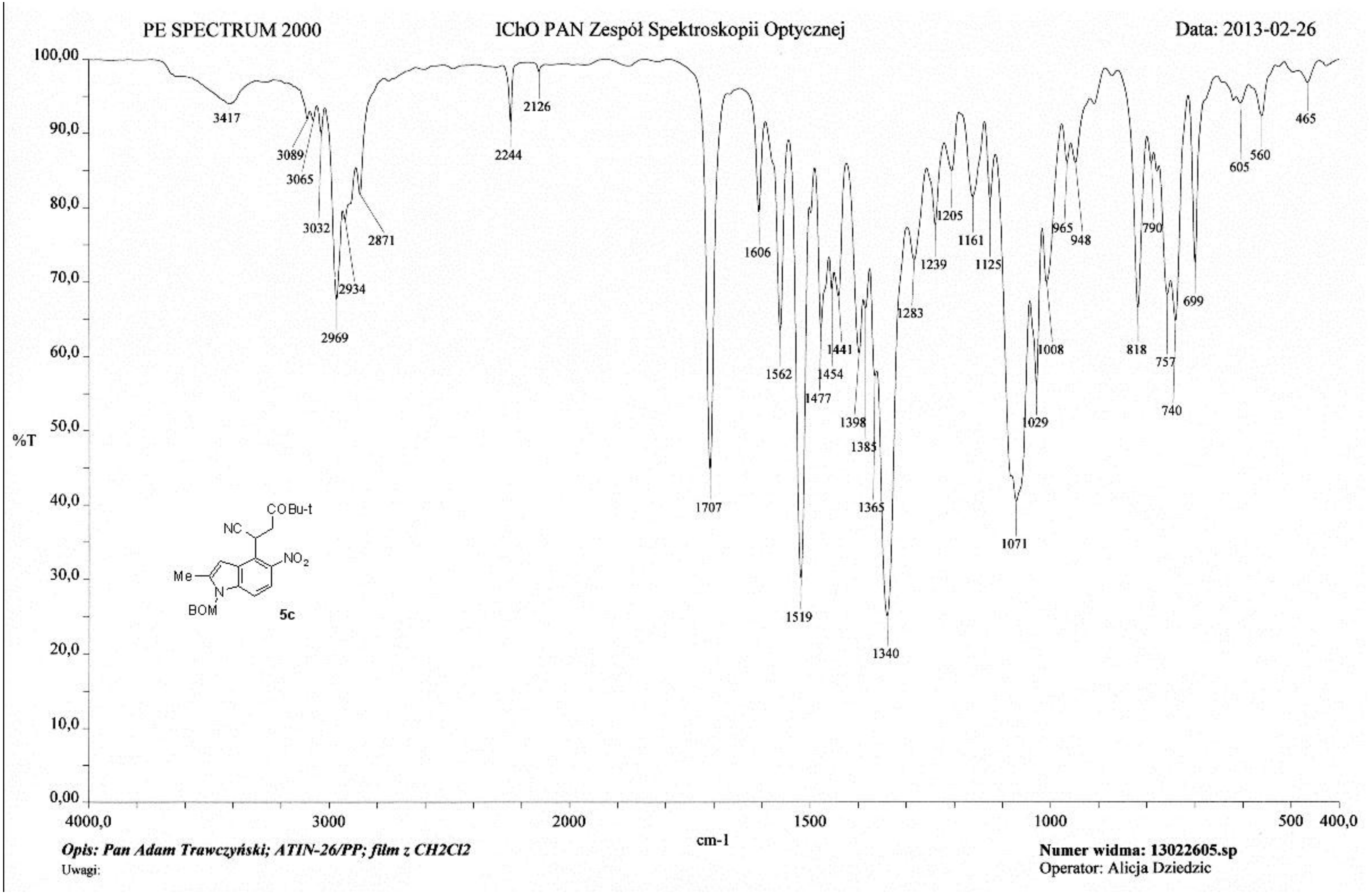
PE SPECTRUM 2000
IChO PAN Zespół Spektroskopii Optycznej
Data: 2013-02-26
%T
cm-1
3417
3089
3065
3032
2969
2934
2871
2244
2126
1707
1606
1562
1519
1477
1454
1441
1398
1385
1365
1340
1283
1239
1205
1161
1125
1071
1029
1008
965
948
818
790
757
740
699
605
560
465
COBu-t
NC
NO2
Me
BOM
5c
Opis: Pan Adam Trawczyński; ATIN-26/PP; film z CH2Cl2
Uwagi:
Numer widma: 13022605.sp
Operator: Alicja Dziedzic

File Name : E:\IChO\Z07_EG\at2505.ms2
Creation Date/Time : 11-09-09 at 14:36:54
File Type : Lo-Res Data - Ctd (Magnet)
File Source : Acquired on MASPEC II system [II32/99D9]
File Title : ATIN-26 (EI 70 eV 33-800)
Operator : Małgorzata Grela
Instrument : AMD 604
Notes : A. Trawczyński

SCAN GRAPH. Flagging=Nominal M/z. Highlighting=Base Peak.
Scan 22#3:14. Entries=640. Base M/z=91.2. 100% Int.=80.2048. Temp =324.

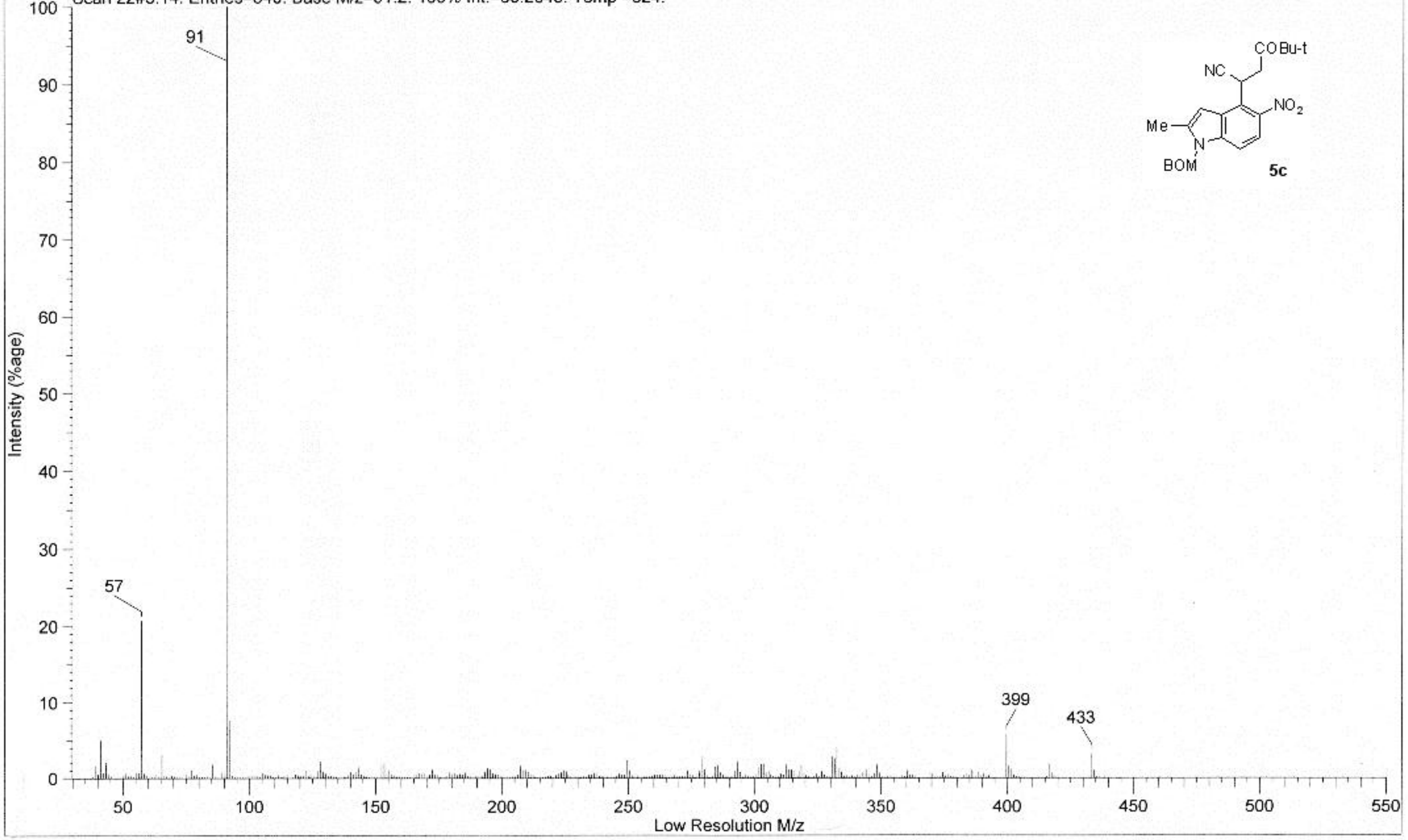

5c

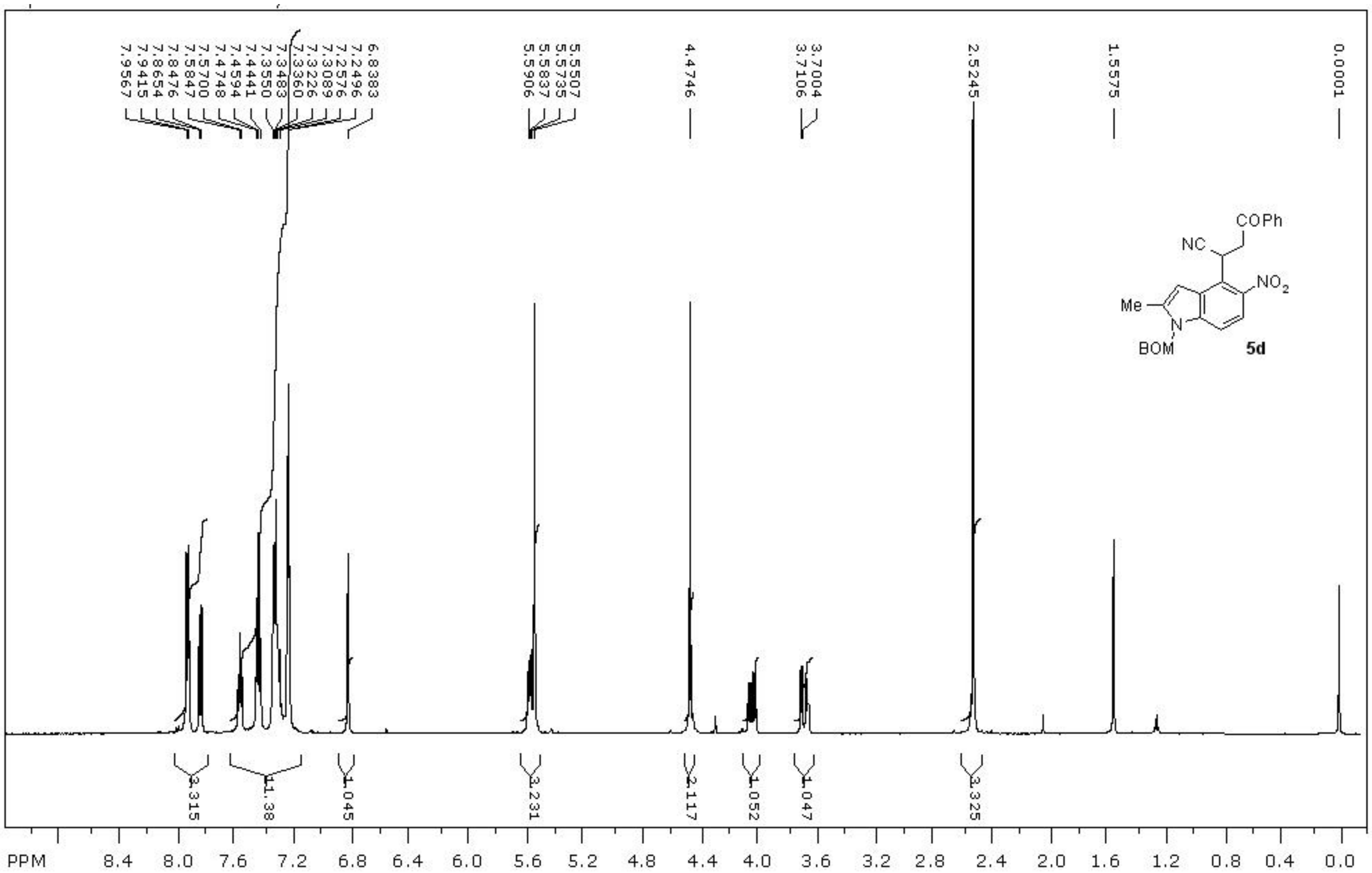

file: ... OINDOLE\ATIN-67\ATIN-67-H1.fid\fid block# 1 expt: "s2pul"
transmitter freq.: 499.834317 MHz
time domain size: 121528 points
width: 12135.92 Hz = 24.2799 ppm = 0.099861 Hz/pt
number of scans: 16

freq. of 0 ppm: 499.831294 MHz
processed size: 262144 complex points
LB: 1.500 GF: 0.0000
Hz/cm: 187.512 ppm/cm: 0.37515

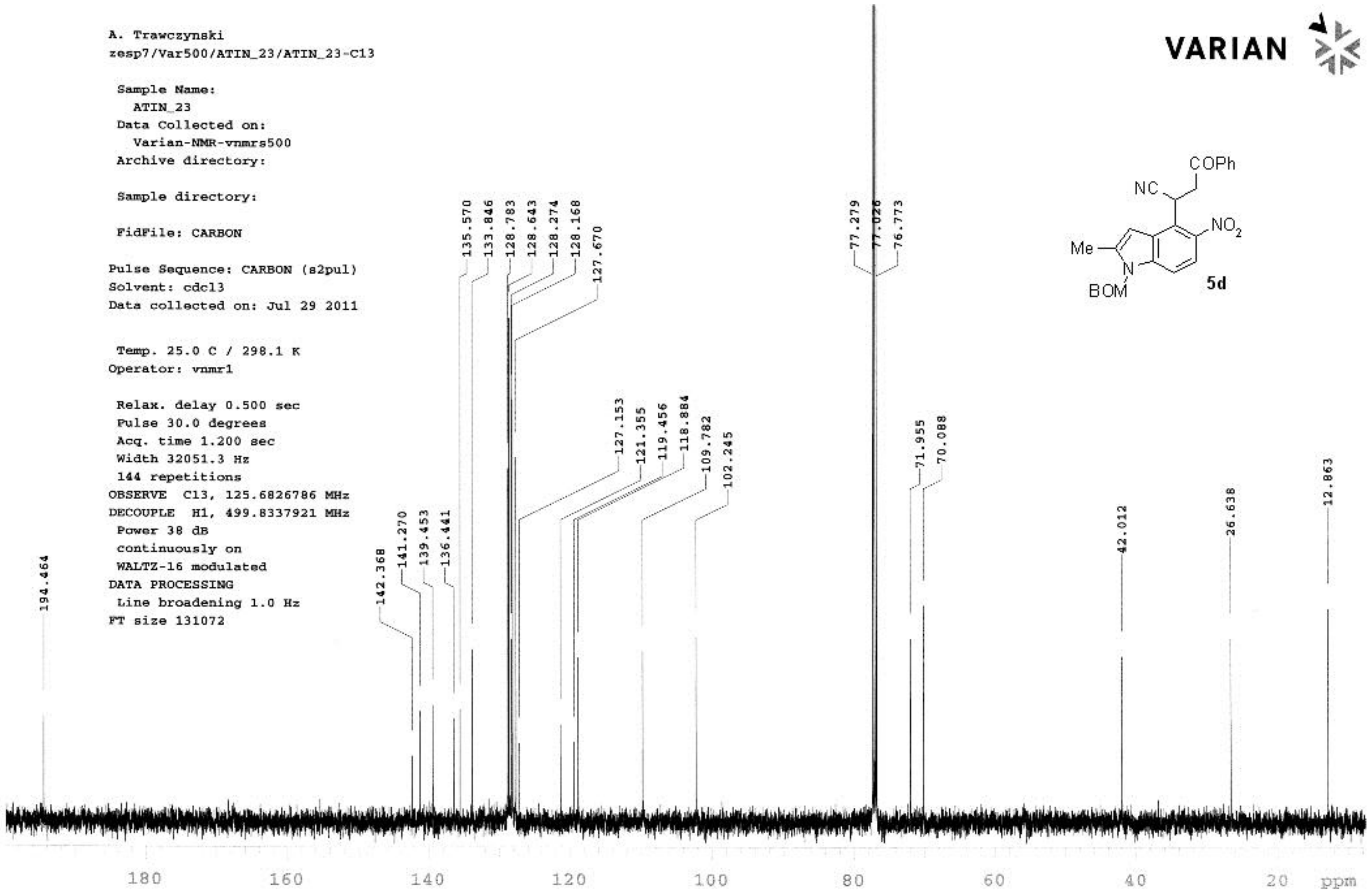
A. Trawczynski
zesp7/Var500/ATIN_23/ATIN_23-C13
Sample Name:
ATIN_23
Data Collected on:
Varian-NMR-vnmrs500
Archive directory:
Sample directory:
FidFile: CARBON
Pulse Sequence: CARBON (s2pul)
Solvent: cdcl3
Data collected on: Jul 29 2011
Temp. 25.0 C / 298.1 K
Operator: vnmr1
Relax. delay 0.500 sec
Pulse 30.0 degrees
Acq. time 1.200 sec
Width 32051.3 Hz
144 repetitions
OBSERVE C13, 125.6826786 MHz
DECOUPLE H1, 499.8337921 MHz
Power 38 dB
continuously on
WALTZ-16 modulated
DATA PROCESSING
Line broadening 1.0 Hz
FT size 131072
VARIAN
COPh
NC
NO2
Me
N
BOM
5d
194.464
142.368
141.270
139.453
136.441
135.570
133.846
128.783
128.643
128.274
128.168
127.670
127.153
121.355
119.456
118.884
109.782
102.245
77.279
77.026
76.773
71.955
70.088
42.012
26.638
12.863
180
160
140
120
100
80
60
40
20
ppm

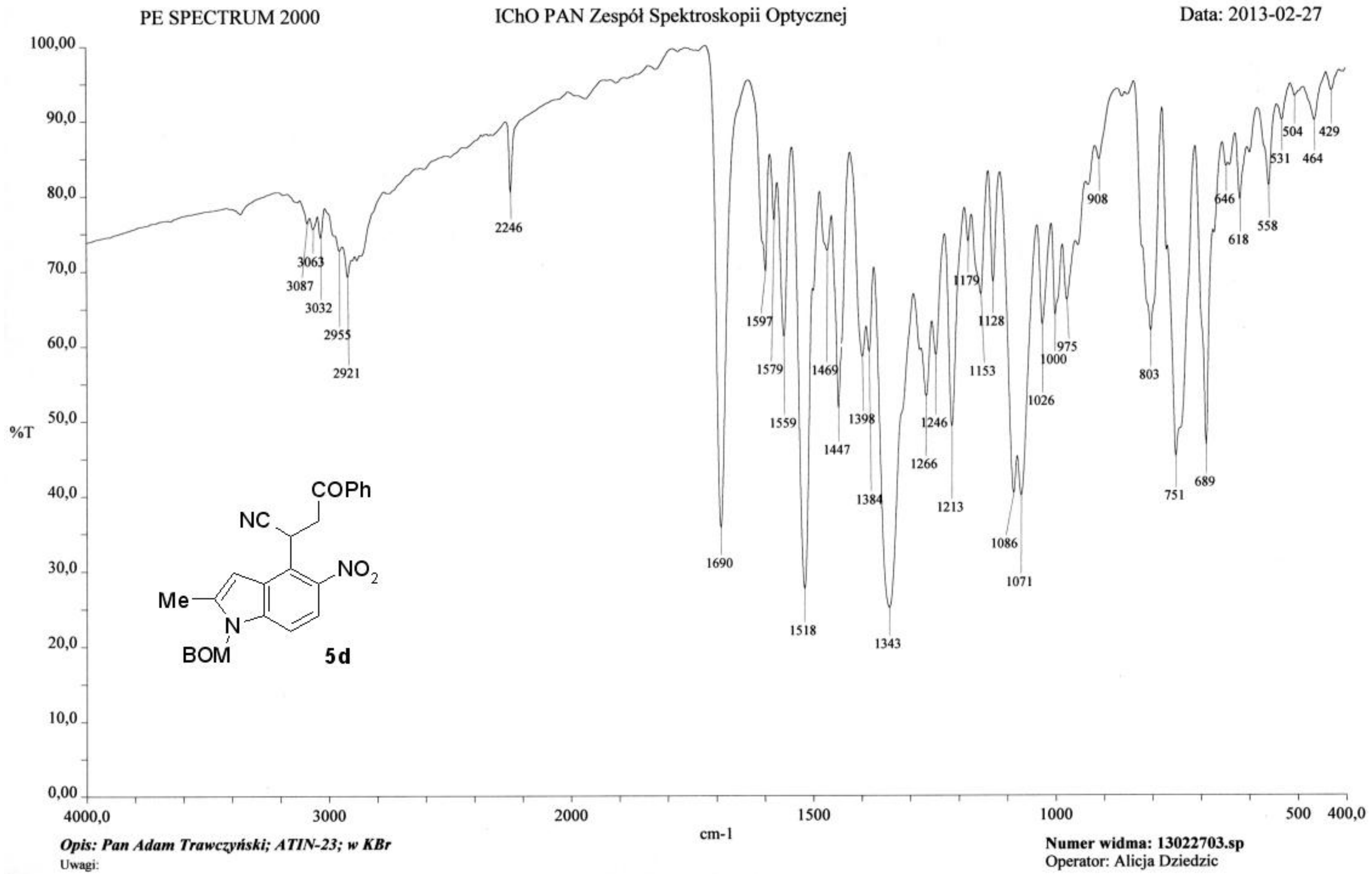
PE SPECTRUM 2000
IChO PAN Zespół Spektroskopii Optycznej
Data: 2013-02-27
%T
100,00
90,0
80,0
70,0
60,0
50,0
40,0
30,0
20,0
10,0
0,00
4000,0
3000
2000
1500
1000
500
400,0
cm-1
3087
3063
3032
2955
2921
2246
1690
1597
1579
1559
1518
1469
1447
1398
1384
1343
1266
1246
1213
1179
1153
1128
1086
1071
1026
1000
975
908
803
751
689
646
618
558
531
504
464
429
COPh
NC
NO_2
Me
N
BOM
5d
Opis: Pan Adam Trawczyński; ATIN-23; w KBr
Uwagi:
Numer widma: 13022703.sp
Operator: Alicja Dziedzic

File Name	:	E:\IChO\Z07_EG\at2209.ms2
Creation Date/Time	:	11-08-01 at 16:50:08
File Type	:	Lo-Res Data - Ctd (Magnet)
File Source	:	Acquired on MASPEC II system [II32/99D9]
File Title	:	ATIN-23 (EI 70 eV 33-800)
Operator	:	Marian Olejnik
Instrument	:	AMD 604
Notes	:	A. Trawczyński

SCAN GRAPH. Flagging=Nominal M/z. Highlighting=Base Peak.
Scan 26#3:06. Entries=569. Base M/z=91.1. 100% Int.=91.8528. Temp =350.

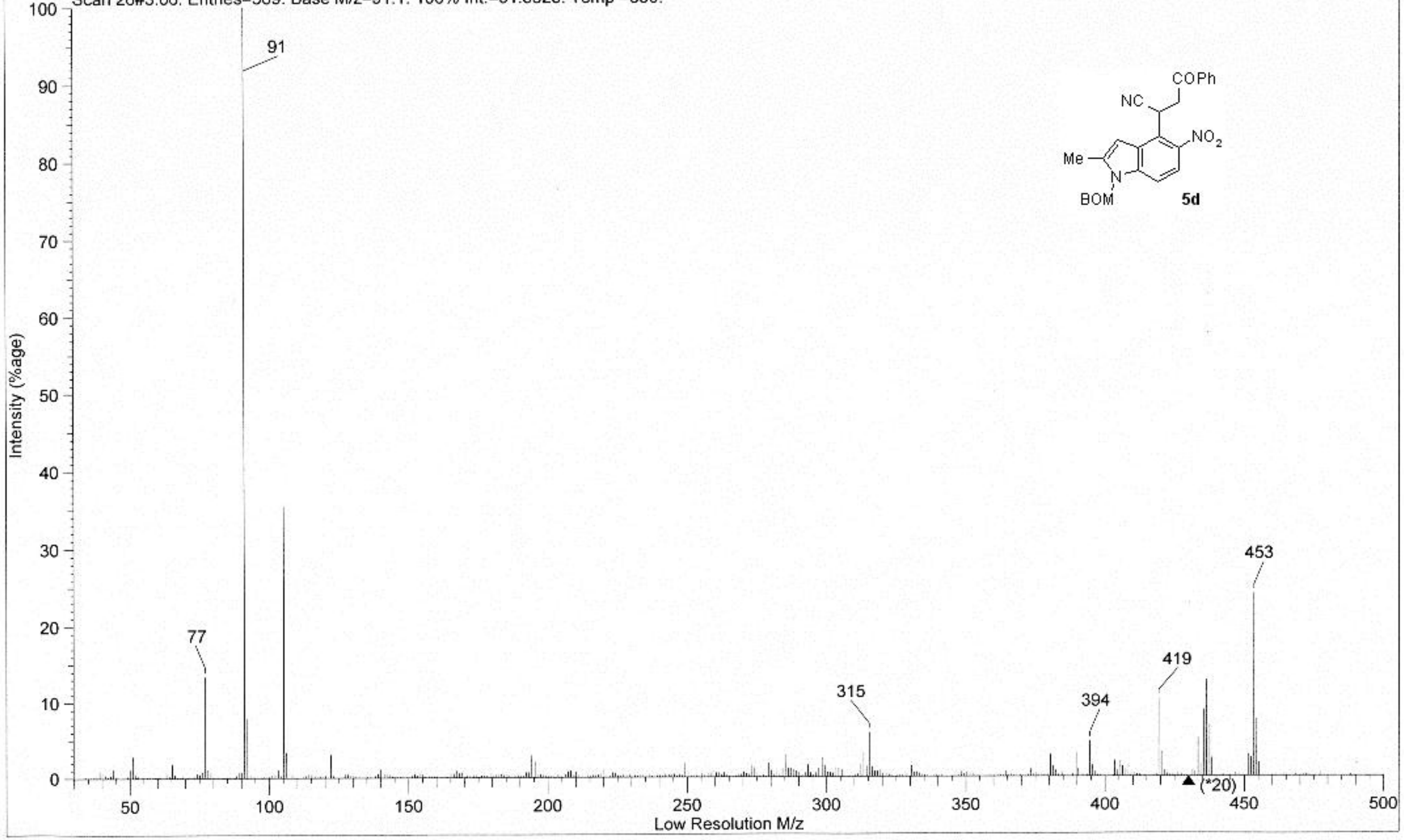

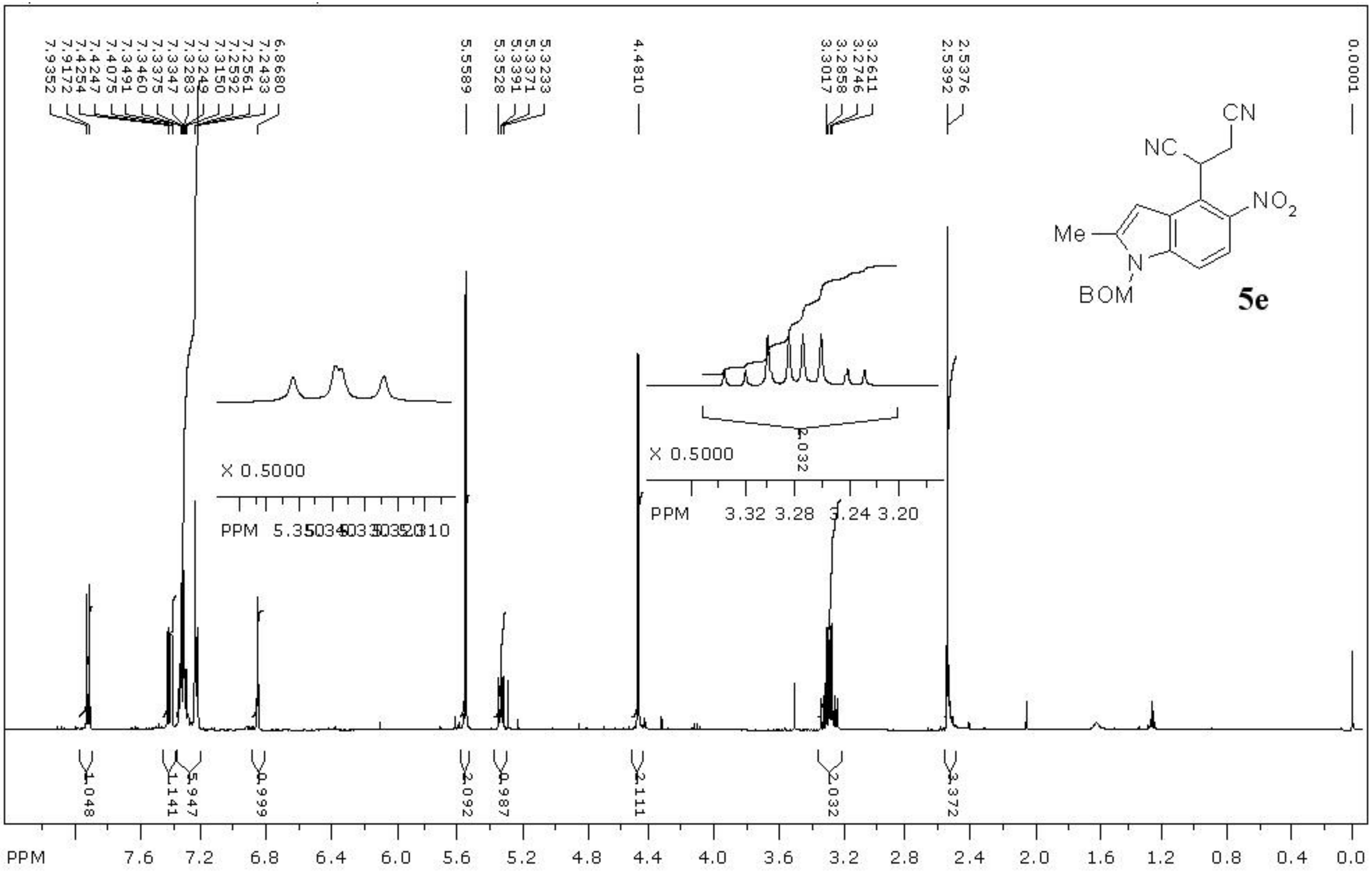

file: ...OINDOLE\ATIN-39\ATIN-39-H1.fid\fid block# 1 expt: "s2pul"
transmitter freq.: 499.834042 MHz
time domain size: 127118 points
width: 10593.22 Hz = 21.1935 ppm = 0.083334 Hz/pt
number of scans: 64

freq. of 0 ppm: 499.831294 MHz
processed size: 131072 complex points
LB: 0.500 GF: 0.0000
Hz/cm: 170.476 ppm/cm: 0.34107

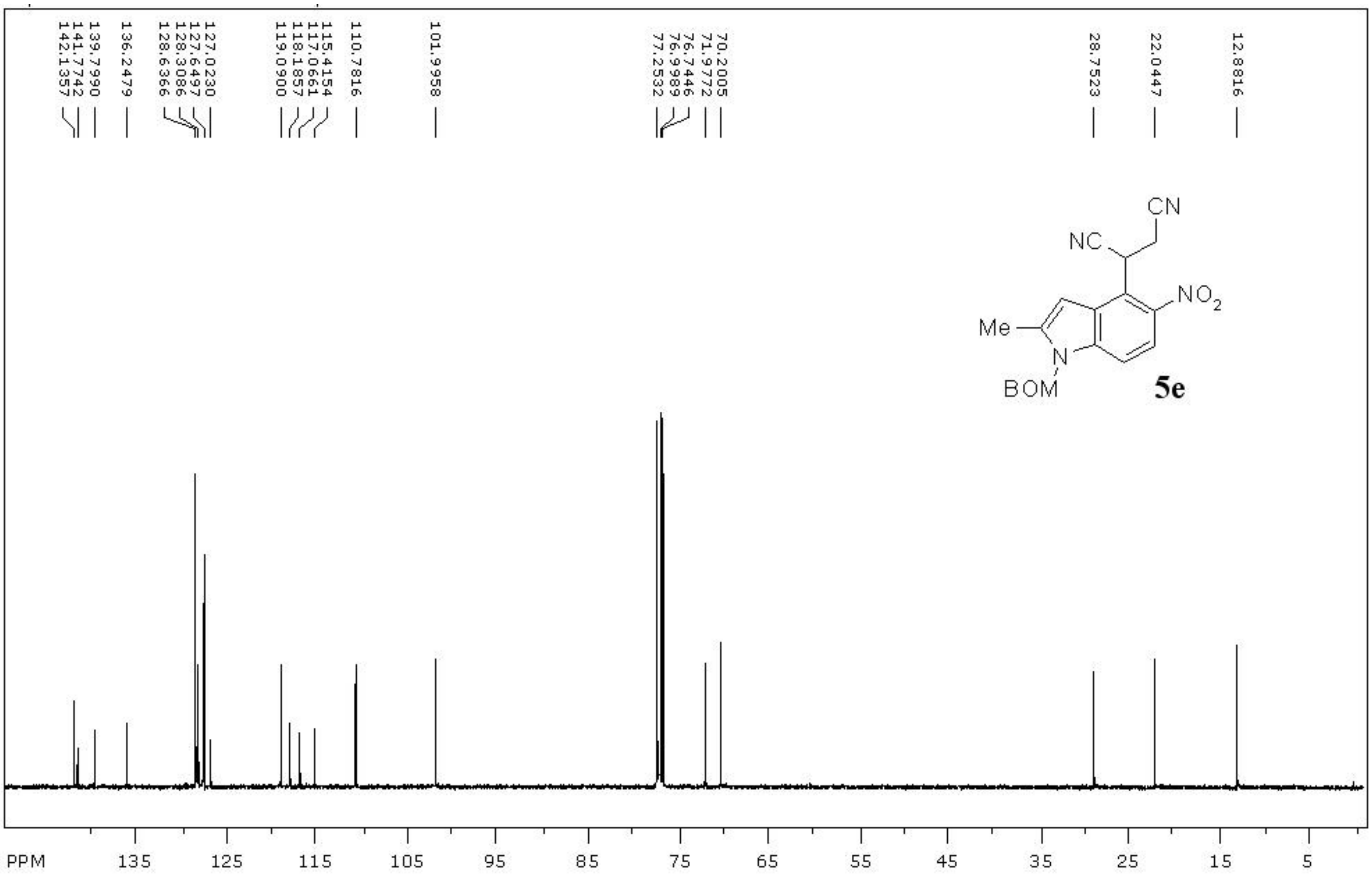

file: ...INDOLE\ATIN-39\ATIN-39-C13.fid\fid block # 1 expt: "s2pul"
transmitter freq.: 125.696504 MHz
time domain size: 80128 points
width: 32051.28 Hz = 254.9894 ppm = 0.400001 Hz/pt
number of scans: 1344

freq. of 0 ppm: 125.682684 MHz
processed size: 131072 complex points
LB: 1.000 GF: 0.0000
Hz/cm: 759.865 ppm/cm: 6.04524

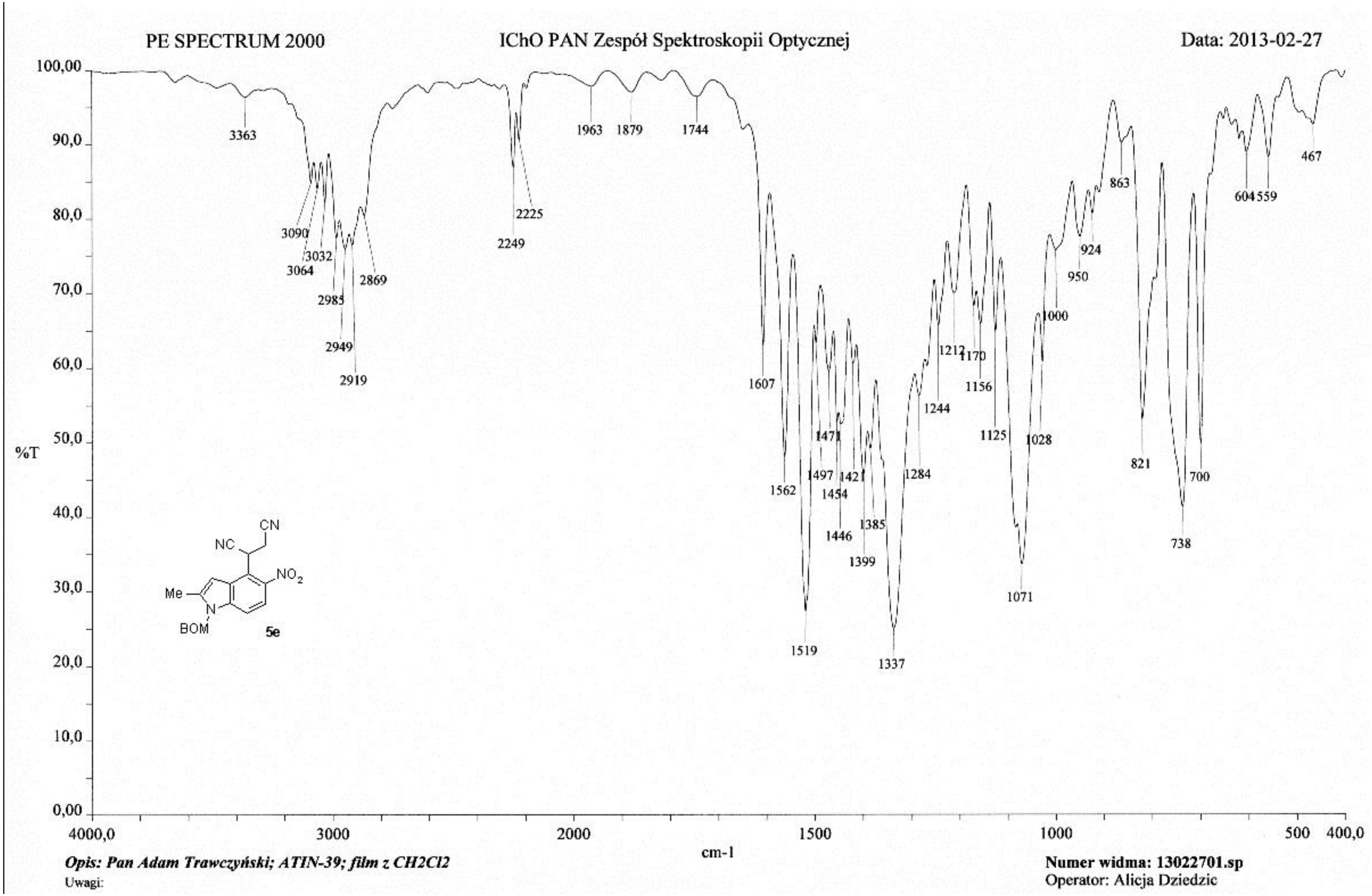
PE SPECTRUM 2000
IChO PAN Zespół Spektroskopii Optycznej
Data: 2013-02-27
%T
cm-1
3363
3090
3064
3032
2985
2949
2919
2869
2249
2225
1963
1879
1744
1607
1562
1519
1497
1471
1454
1446
1421
1399
1385
1337
1284
1244
1212
1170
1156
1125
1071
1028
1000
950
924
863
821
738
700
604
559
467
NC
CN
NO2
Me
N
BOM
5e
Opis: Pan Adam Trawczyński; ATIN-39; film z CH2Cl2
Uwagi:
Numer widma: 13022701.sp
Operator: Alicja Dziedzic

File Name : E:\IChO\Z07_EG\at2986.ms2
Creation Date/Time : 11-10-10 at 13:10:43
File Type : Lo-Res Data - Ctd (Magnet)
File Source : Acquired on MASPEC II system [II32/99D9]
File Title : ATIN-39 (EI 70 eV 33-800)
Operator : Marian Olejnik
Instrument : AMD 604
Notes : A. Trawczyński

SCAN GRAPH. Flagging=Nominal M/z. Highlighting=Base Peak.
Scan 178#21:32. Entries=670. Base M/z=91.2. 100% Int.=51.8912. Temp =302.

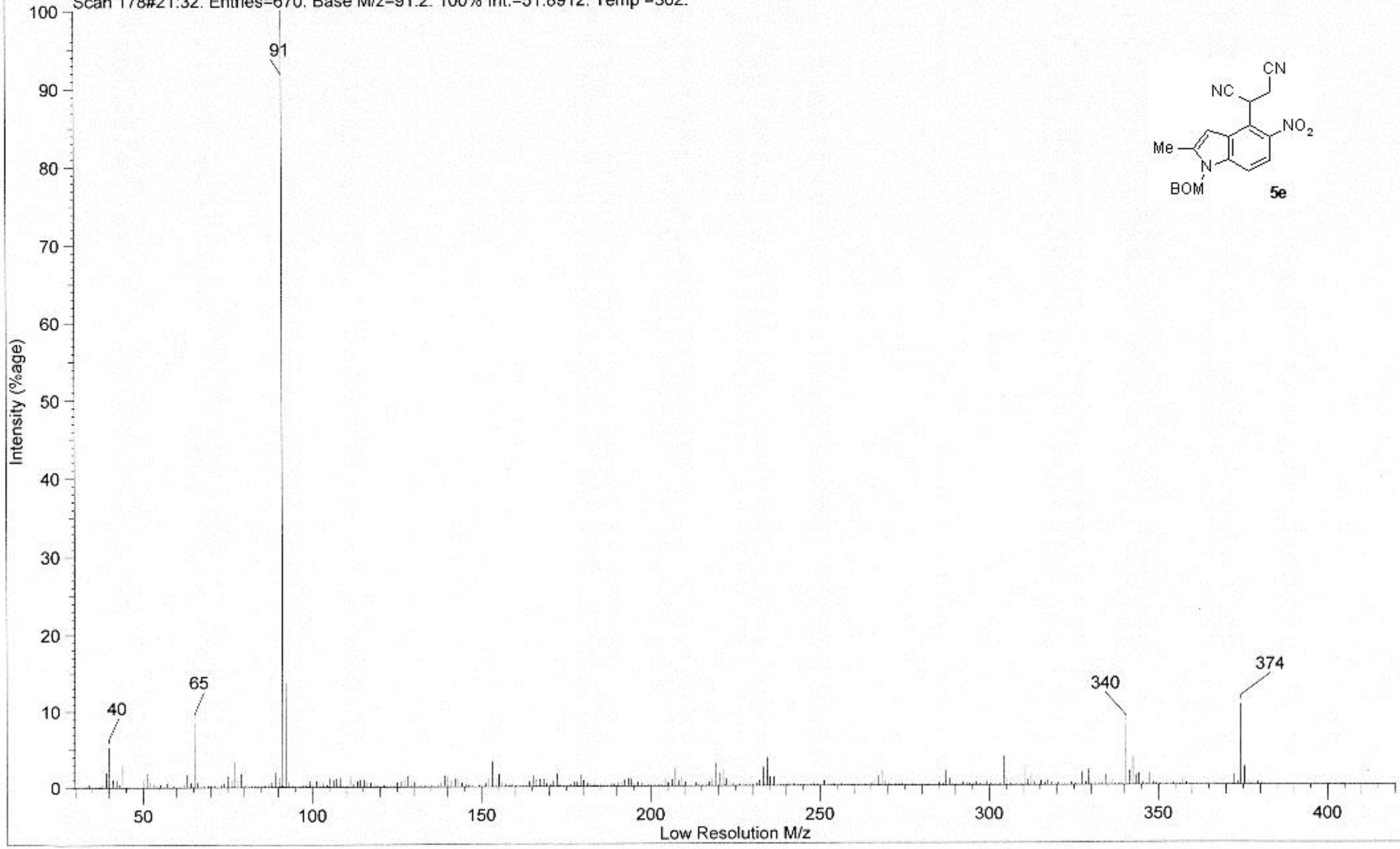

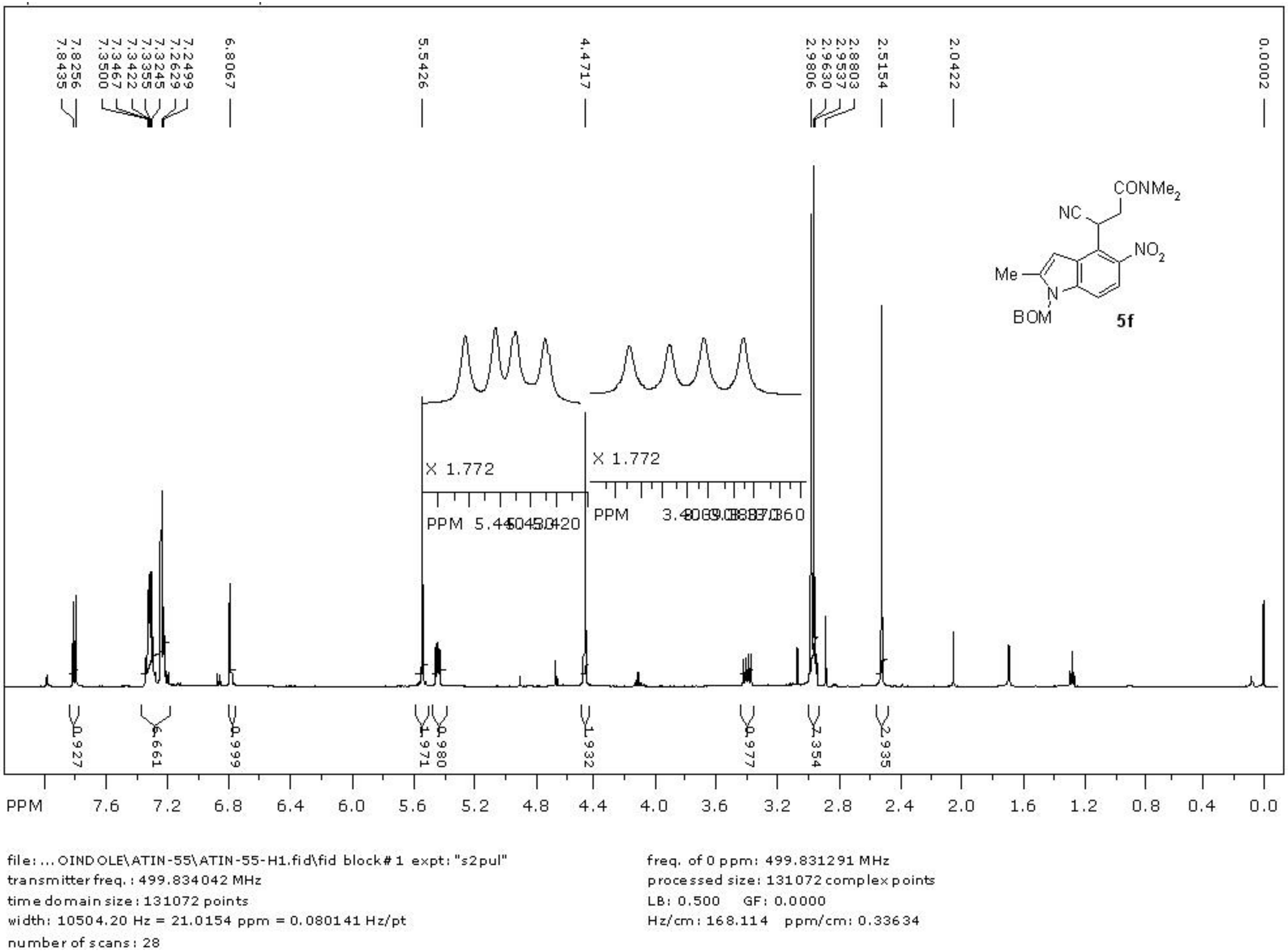
CONMe2
NC
NO2
Me
N
BOM
5f
7.8435
7.8256
7.3500
7.3467
7.3422
7.3355
7.3245
7.2629
7.2499
6.8067
5.5426
4.4717
2.9806
2.9630
2.9537
2.8803
2.5154
2.0422
0.0002
X 1.772
X 1.772
PPM
0.927
6.661
0.999
1.971
0.980
1.932
0.977
7.354
2.935
PPM 7.6 7.2 6.8 6.4 6.0 5.6 5.2 4.8 4.4 4.0 3.6 3.2 2.8 2.4 2.0 1.6 1.2 0.8 0.4 0.0
file: ... OINDOLE\ATIN-55\ATIN-55-H1.fid\fid block# 1 expt: "s2pul"
transmitter freq.: 499.834042 MHz
time domain size: 131072 points
width: 10504.20 Hz = 21.0154 ppm = 0.080141 Hz/pt
number of scans: 28
freq. of 0 ppm: 499.831291 MHz
processed size: 131072 complex points
LB: 0.500 GF: 0.0000
Hz/cm: 168.114 ppm/cm: 0.33634

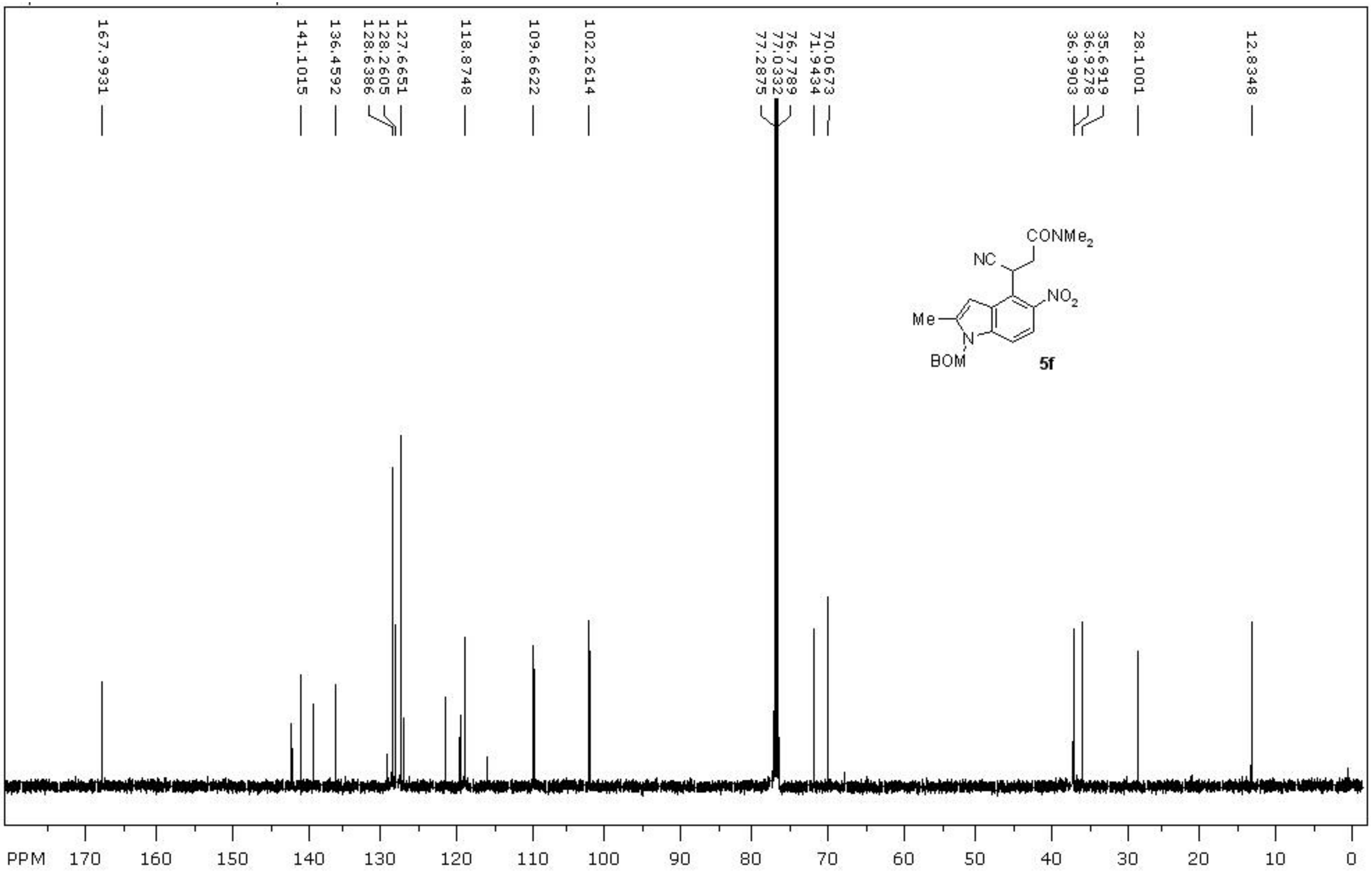

file: ...INDOLE\ATIN-55\ATIN-55-C13.fid\fid block # 1 expt: "s2pul"
transmitter freq.: 125.696504 MHz
time domain size: 90910 points
width: 37878.79 Hz = 301.3512 ppm = 0.416663 Hz/pt
number of scans: 368

freq. of 0 ppm: 125.682679 MHz
processed size: 131072 complex points
LB: 1.000 GF: 0.0000
Hz/cm: 921.501 ppm/cm: 7.33116

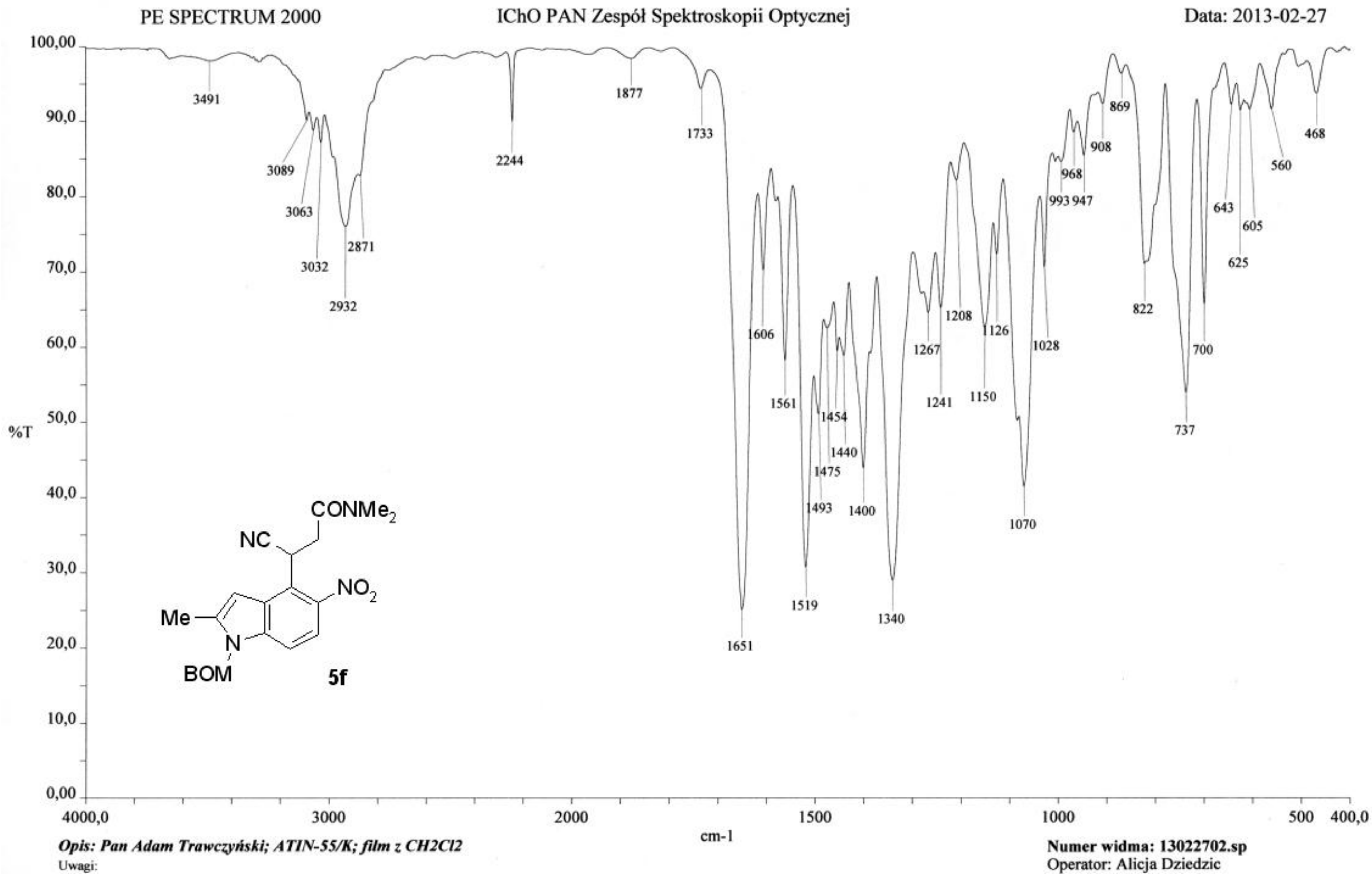
PE SPECTRUM 2000
IChO PAN Zespół Spektroskopii Optycznej
Data: 2013-02-27
%T
cm-1
3491
3089
3063
3032
2932
2871
2244
1877
1733
1651
1606
1561
1519
1493
1475
1454
1440
1400
1340
1267
1241
1208
1150
1126
1070
1028
993
968
947
908
869
822
737
700
643
625
605
560
468
CONMe2
NC
NO2
Me
N
BOM
5f
Opis: Pan Adam Trawczyński; ATIN-55/K; film z CH2Cl2
Uwagi:
Numer widma: 13022702.sp
Operator: Alicja Dziedzic

File Name	:	E:\IChO\Z07_EG\at3540.ms2
Creation Date/Time	:	11-12-07 at 10:13:45
File Type	:	Lo-Res Data - Ctd (Magnet)
File Source	:	Acquired on MASPEC II system [II32/99D9]
File Title	:	ATIN-55 (EI 70 eV 33-800)
Operator	:	Marian Olejnik
Instrument	:	AMD 604
Notes	:	A. Trawczyński

SCAN GRAPH. Flagging=Nominal M/z. Highlighting=Base Peak.
Scan 57#6:40. Entries=613. Base M/z=72.2. 100% Int.=68.4032. Temp =256.

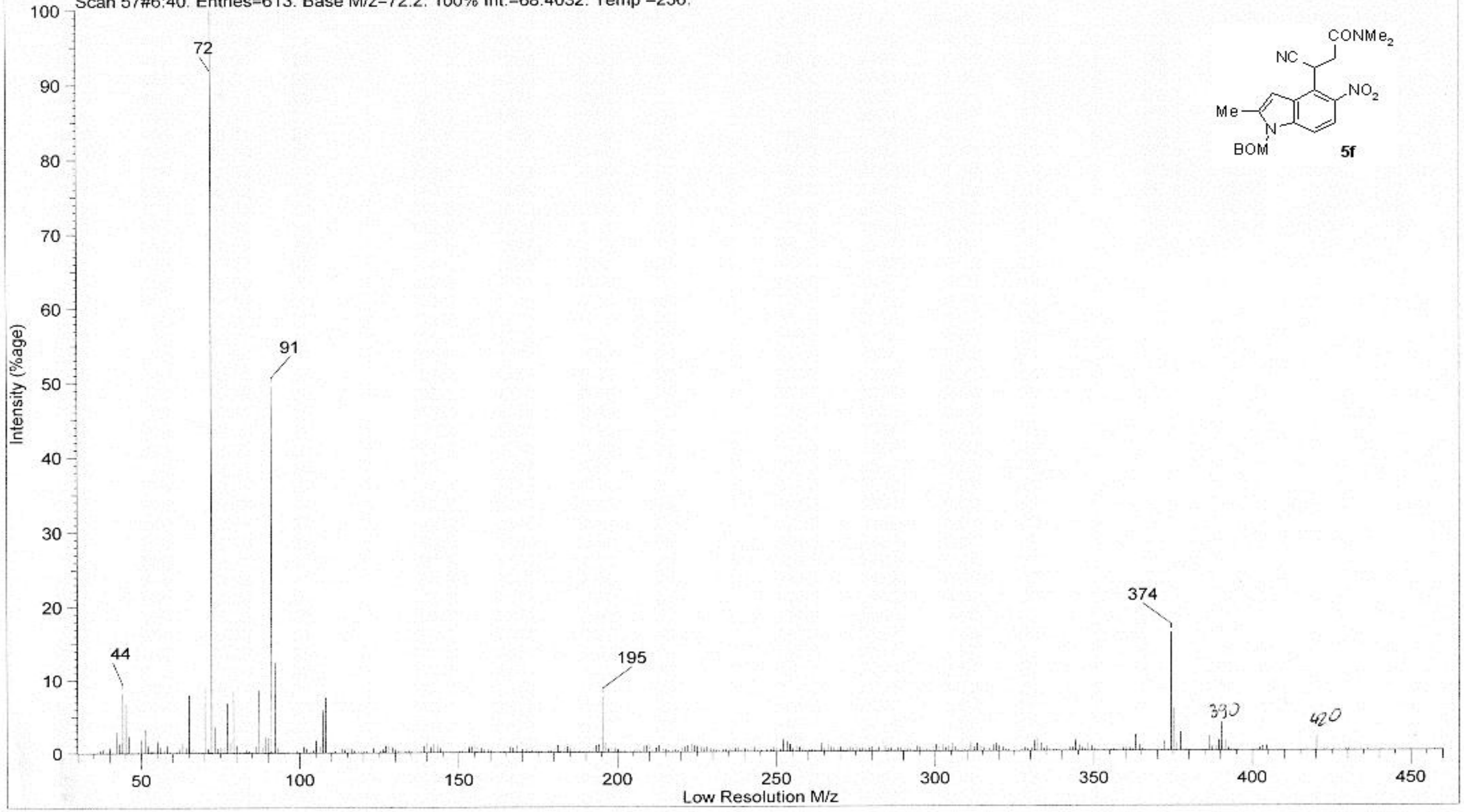

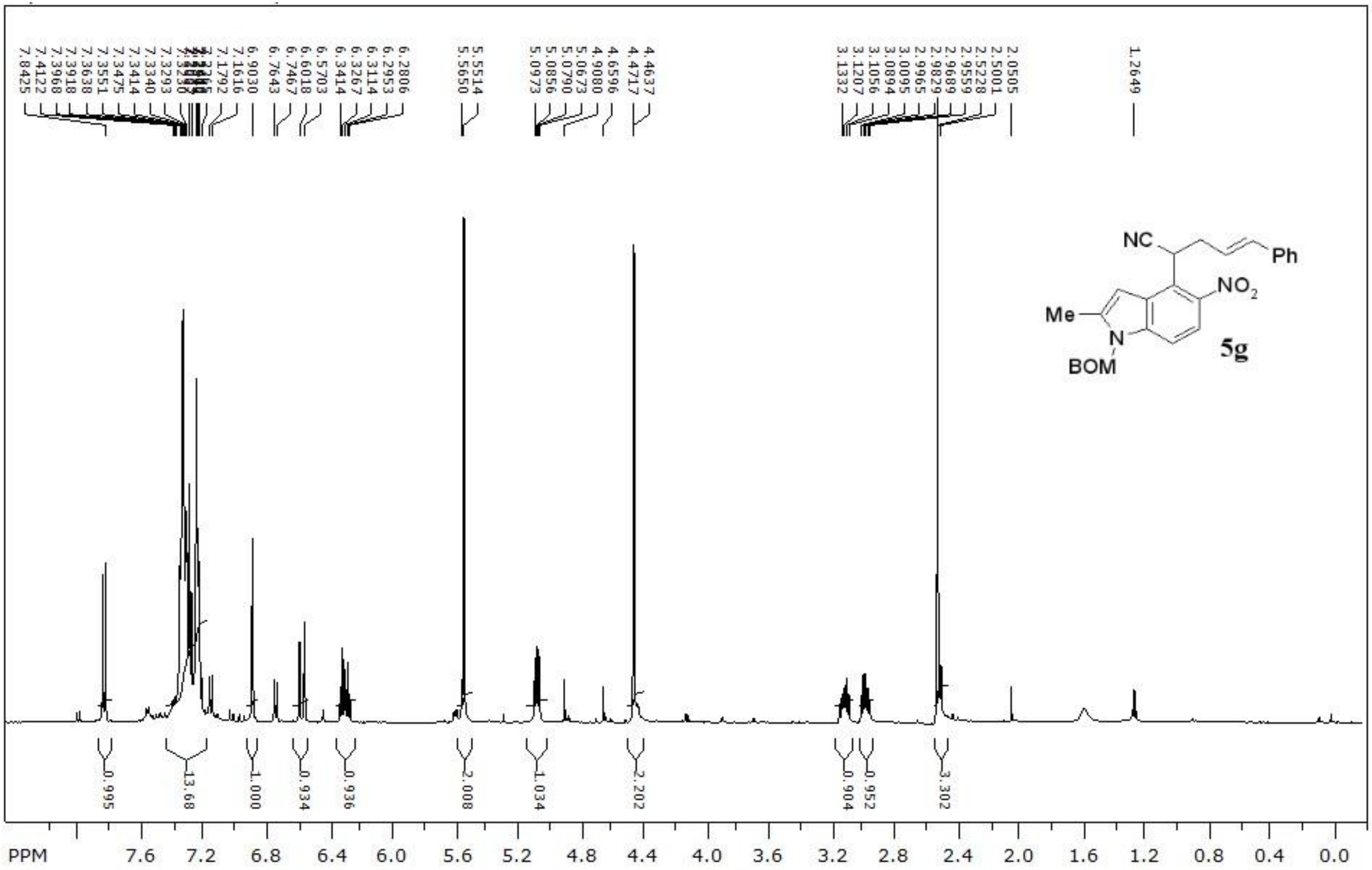

file: ...OINDOLE\ATIN-56\ATIN-56-H1.fid\fid block# 1 expt: "s2pul"
transmitter freq.: 499.834042 MHz
time domain size: 131072 points
width: 10504.20 Hz = 21.0154 ppm = 0.080141 Hz/pt
number of scans: 64

freq. of 0 ppm: 499.831293 MHz
processed size: 131072 complex points
LB: 0.500 GF: 0.0000
Hz/cm: 173.462 ppm/cm: 0.34704

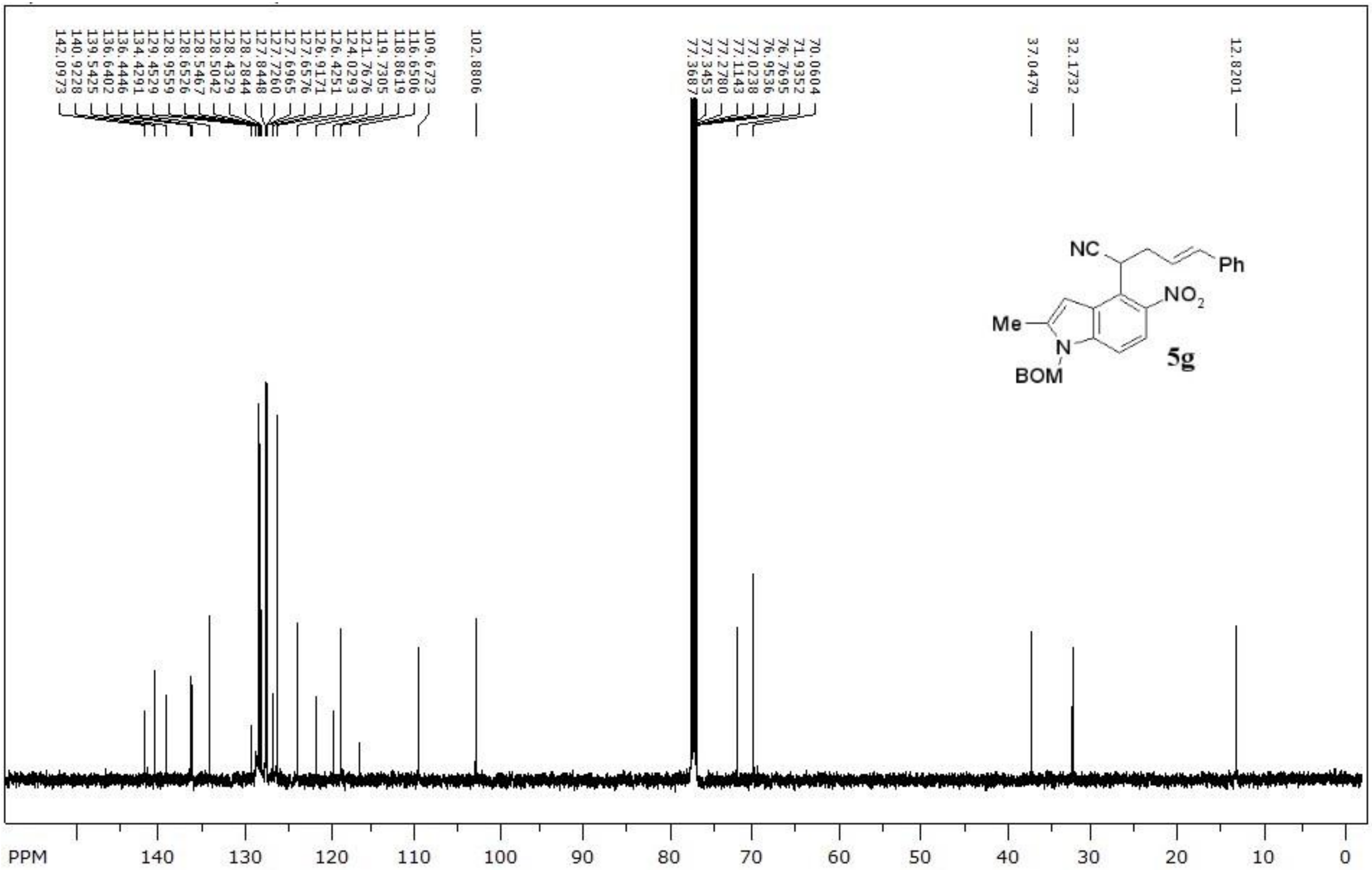

file: ...INDOLE\ATIN-56\ATIN-56-C13.fid\fid block# 1 expt: "s2pul"
transmitter freq.: 125.696504 MHz
time domain size: 90910 points
width: 37878.79 Hz = 301.3512 ppm = 0.416663 Hz/pt
number of scans: 448

freq. of 0 ppm: 125.682679 MHz
processed size: 131072 complex points
LB: 1.000 GF: 0.0000
Hz/cm: 808.304 ppm/cm: 6.43060

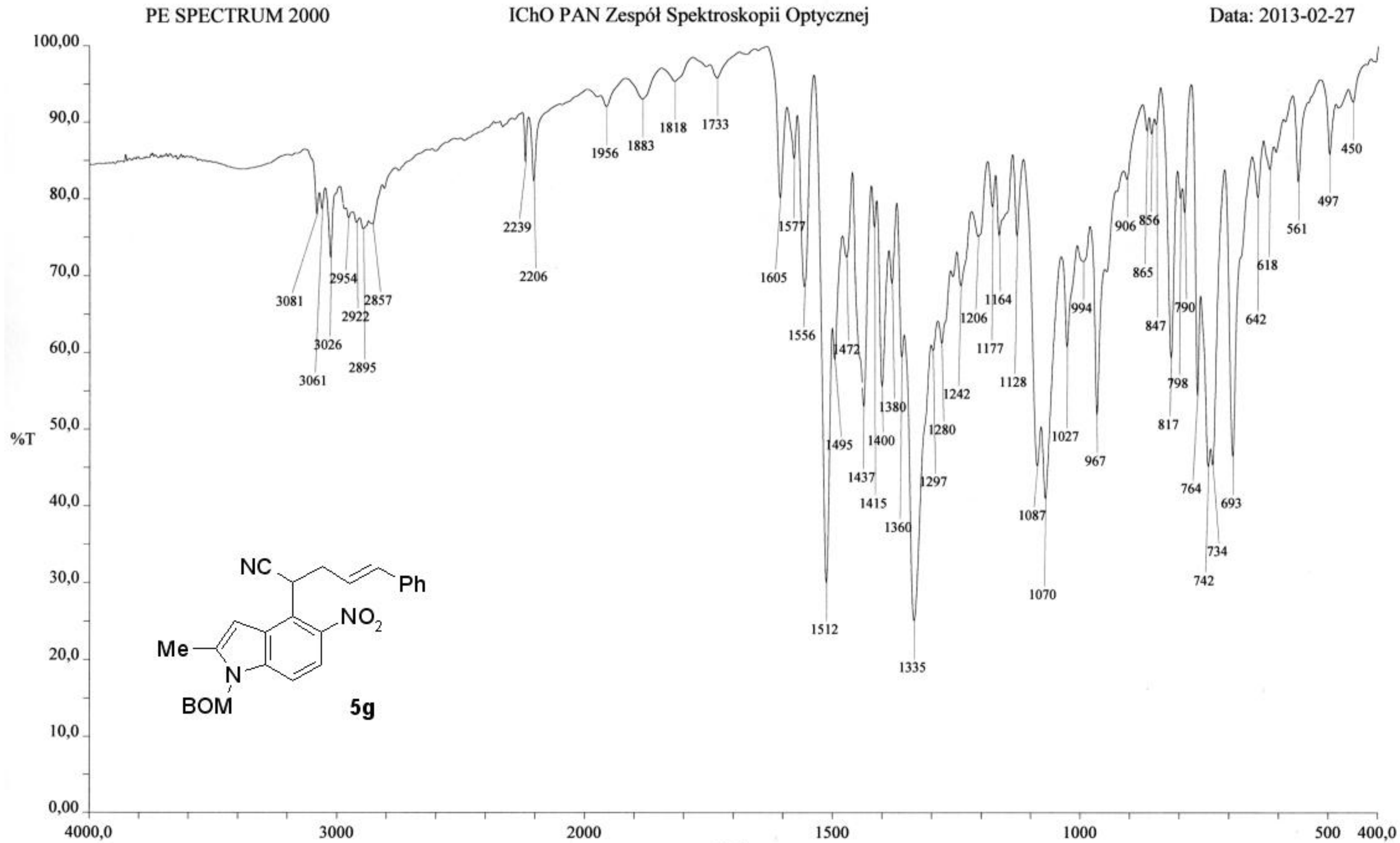

Opis: Pan Adam Trawczyński; ATIN-56; w KBr

Uwagi:

Numer widma: 13022707.sp

Operator: Alicja Dziedzic

File Name	:	E:\IChO\Z07_EG\at3622.ms2
Creation Date/Time	:	11-12-07 at 12:35:27
File Type	:	Lo-Res Data - Ctd (Magnet)
File Source	:	Acquired on MASPEC II system [II32/99D9]
File Title	:	ATIN-56 (EI 70 eV 33-800)
Operator	:	Marian Olejnik
Instrument	:	AMD 604
Notes	:	A. Trawczyński

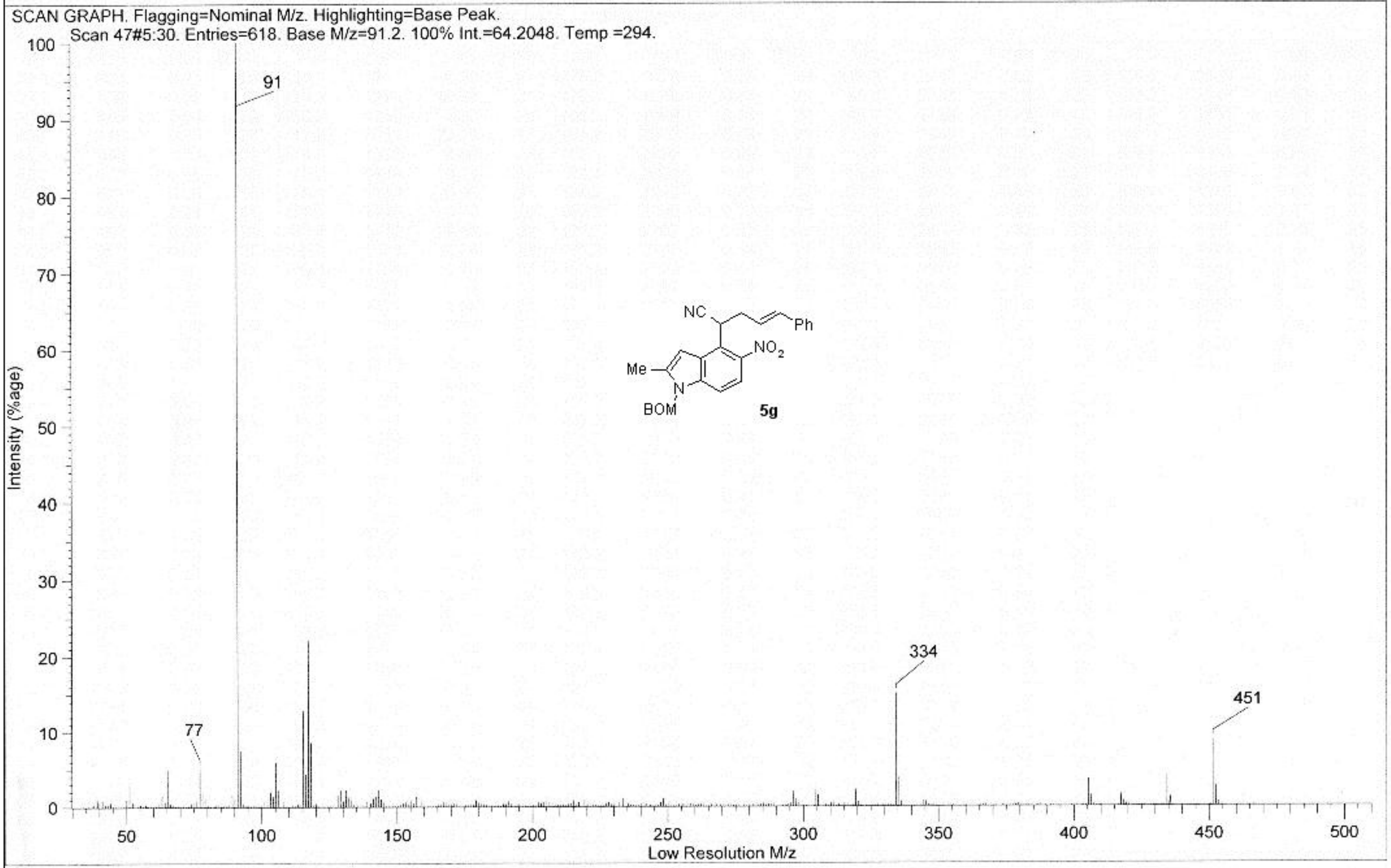

SpinWorks 3: A. Trawczynski

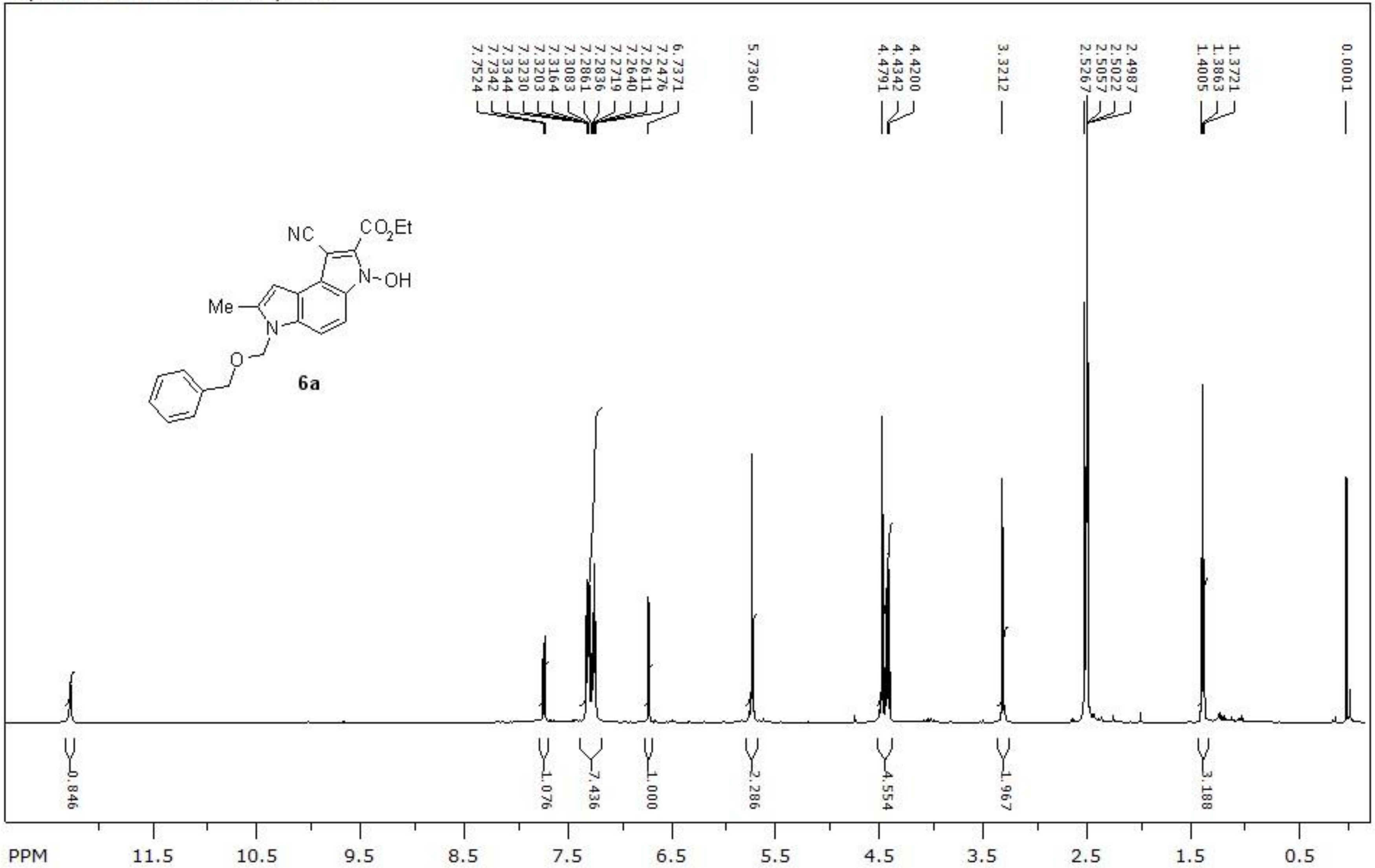

file: ...NDOLE\ATIN-8-3\ATIN-8-3-H1.fid\fid block# 1 expt: "s2pul"
transmitter freq.: 499.836166 MHz
time domain size: 131072 points
width: 10245.90 Hz = 20.4985 ppm = 0.078170 Hz/pt
number of scans: 36

freq. of 0 ppm: 499.833658 MHz
processed size: 131072 complex points
LB: 0.500 GF: 0.0000
Hz/cm: 262.413 ppm/cm: 0.52500

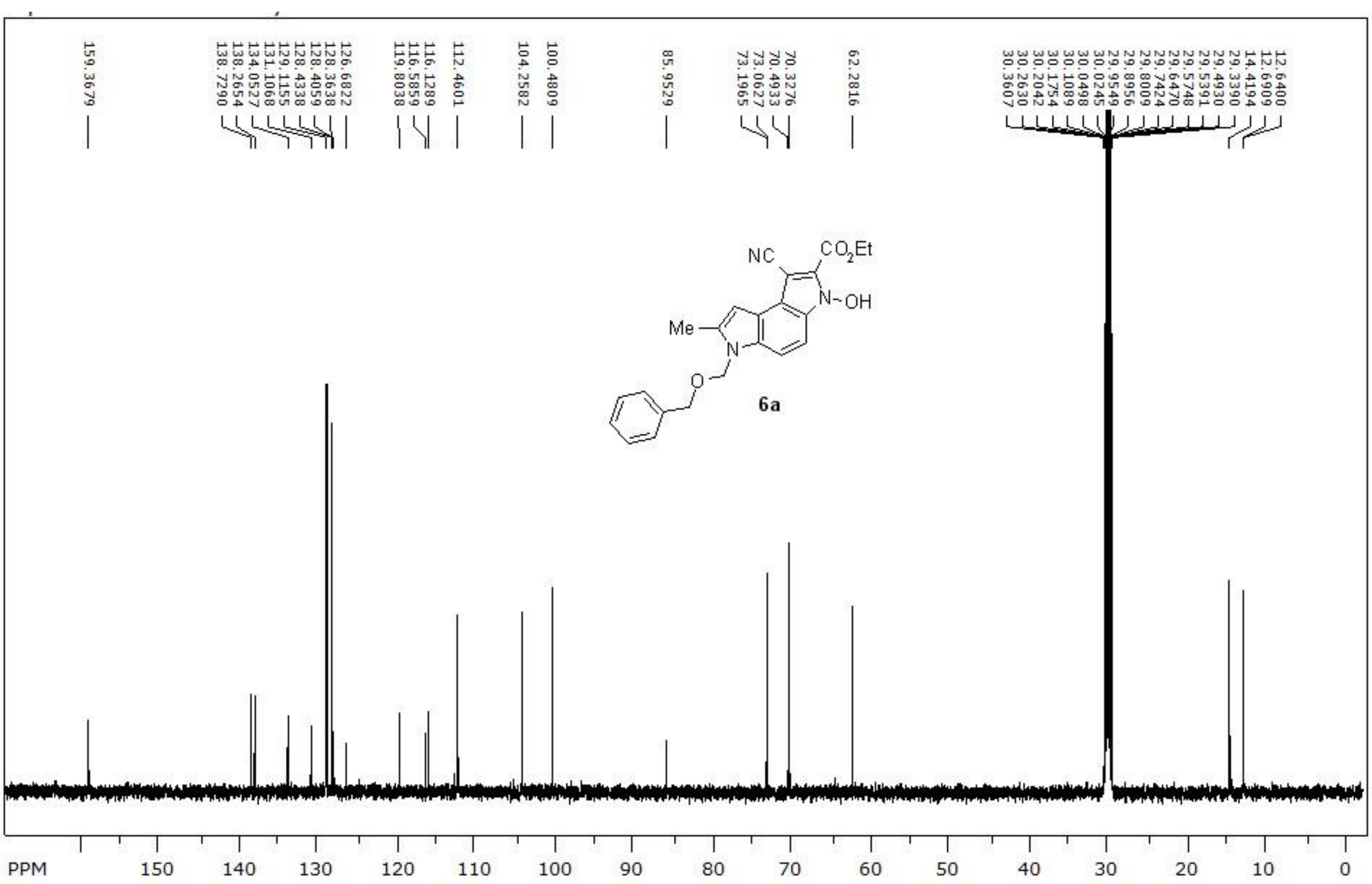

file: ...INDOLE\ATIN-31\ATIN-31-C13.fid\fid block# 1 expt: "s2pul"
transmitter freq.: 125.697156 MHz
time domain size: 90910 points
width: 37878.79 Hz = 301.3496 ppm = 0.416663 Hz/pt
number of scans: 400

freq. of 0 ppm: 125.683223 MHz
processed size: 131072 complex points
LB: 1.000 GF: 0.0000
Hz/cm: 866.999 ppm/cm: 6.89752

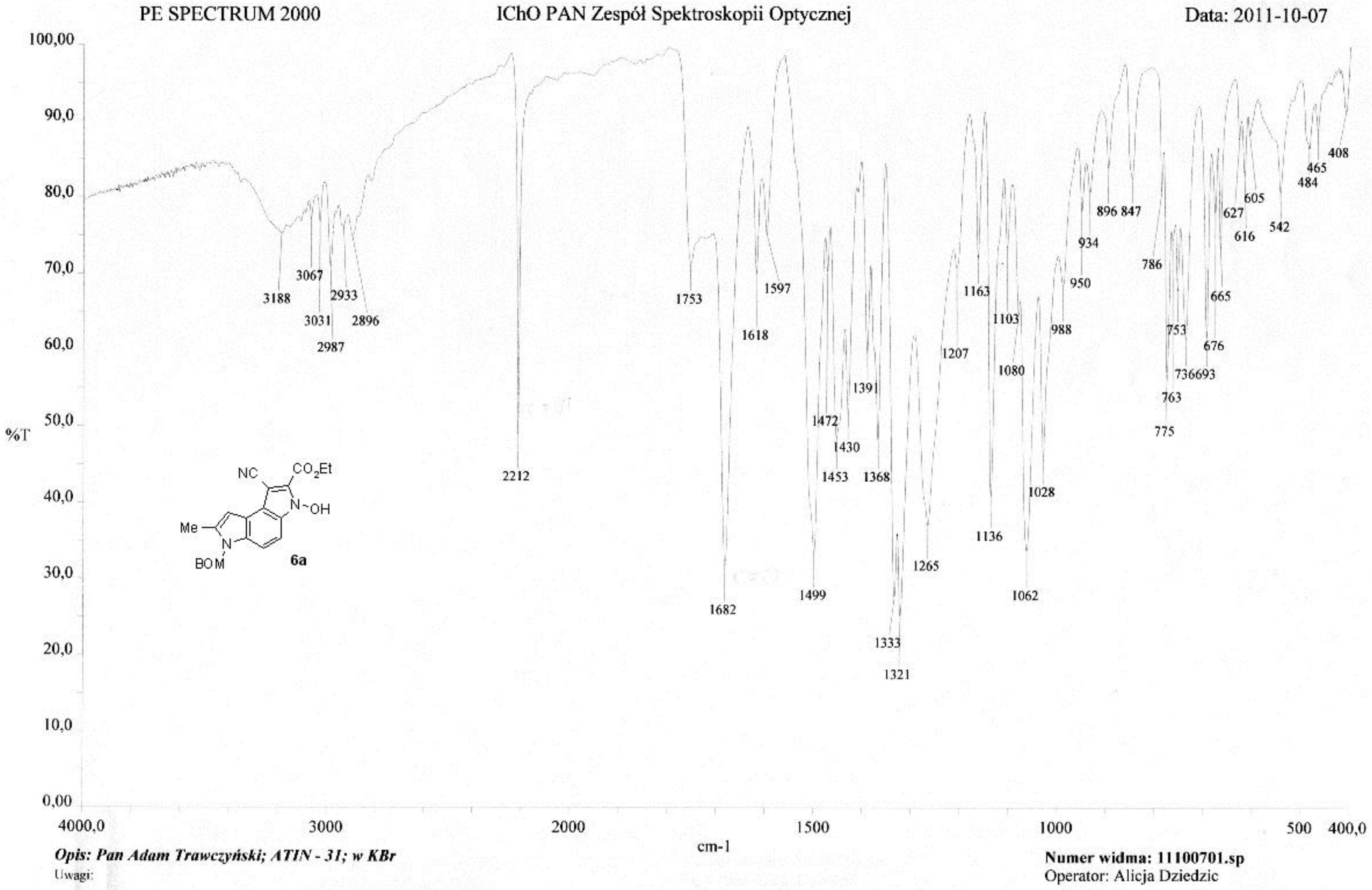
PE SPECTRUM 2000
IChO PAN Zespół Spektroskopii Optycznej
Data: 2011-10-07
%T
cm-1
3188
3067
3031
2987
2933
2896
2212
1753
1682
1618
1597
1499
1472
1453
1430
1391
1368
1333
1321
1265
1207
1163
1136
1103
1080
1062
1028
988
950
934
896 847
786
775
763
753
736693
676
665
627
616
605
542
484
465
408
NC
CO2Et
N-OH
Me
N
BOM
6a
Opis: Pan Adam Trawczyński; ATIN - 31; w KBr
Uwagi:
Numer widma: 11100701.sp
Operator: Alicja Dziedzic

File Name	:	E:\IChO\Z07_EG\at1155.ms2
Creation Date/Time	:	11-04-18 at 10:25:23
File Type	:	Lo-Res Data - Ctd (Magnet)
File Source	:	Acquired on MASPEC II system [II32/99D9]
File Title	:	ATIN-8-3 (EI 70 eV 33-800)
Operator	:	Marian Olejnik
Instrument	:	AMD 604
Notes	:	A. Trawczyński

SCAN GRAPH. Flagging=Nominal M/z. Highlighting=Base Peak.
Scan 37#4:27. Entries=843. Base M/z=91.1. 100% Int.=87.8592. Temp =280.

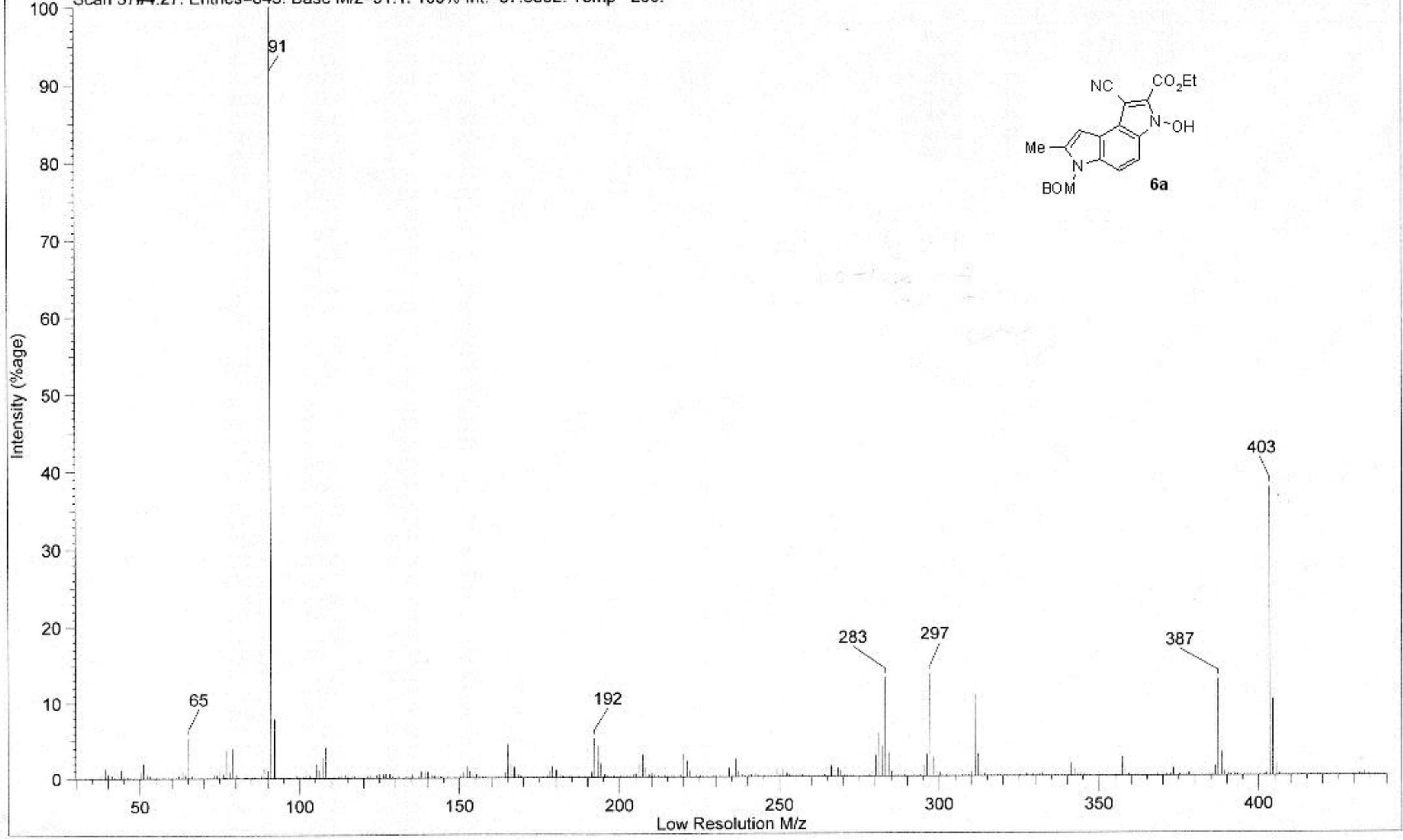

6a

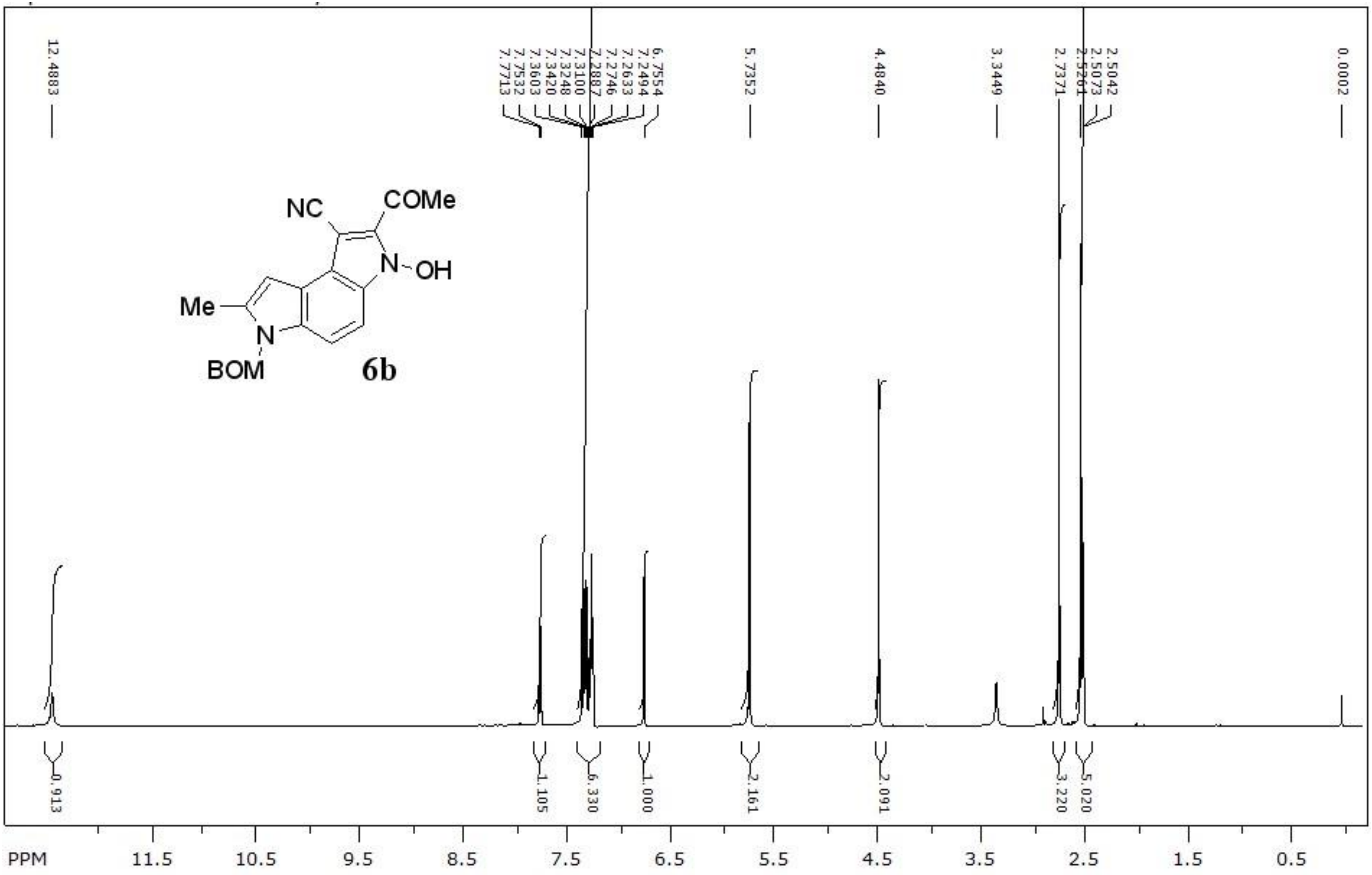

file: ...OINDOLE\ATIN-52\ATIN-52-H1.fid\fid block# 1 expt: "s2pul"
transmitter freq.: 499.836466 MHz
time domain size: 127118 points
width: 10593.22 Hz = 21.1934 ppm = 0.083334 Hz/pt
number of scans: 64

freq. of 0 ppm: 499.833657 MHz
processed size: 131072 complex points
LB: 1.061 GF: 0.0000
Hz/cm: 262.867 ppm/cm: 0.52591

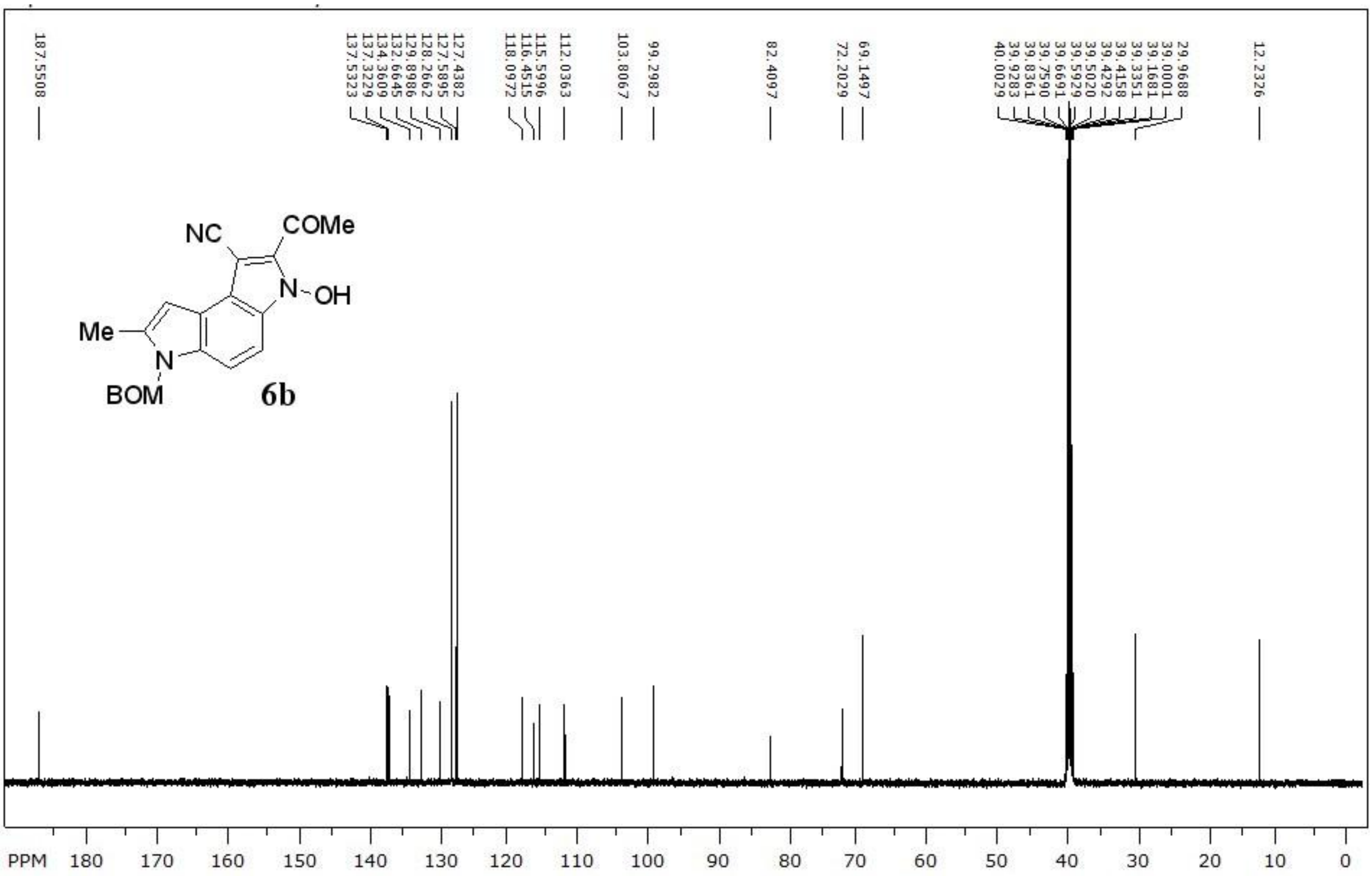

file: ...INDOLE\ATIN-52\ATIN-52-C13.fid\fid block# 1 expt: "s2pul"
transmitter freq.: 125.697101 MHz
time domain size: 80128 points
width: 32051.28 Hz = 254.9882 ppm = 0.400001 Hz/pt
number of scans: 528

freq. of 0 ppm: 125.683336 MHz
processed size: 131072 complex points
LB: 0.500 GF: 0.0000
Hz/cm: 980.787 ppm/cm: 7.80278

PE SPECTRUM 2000 IChO PAN Zespół Spektroskopii Optycznej Data: 2012-03-28

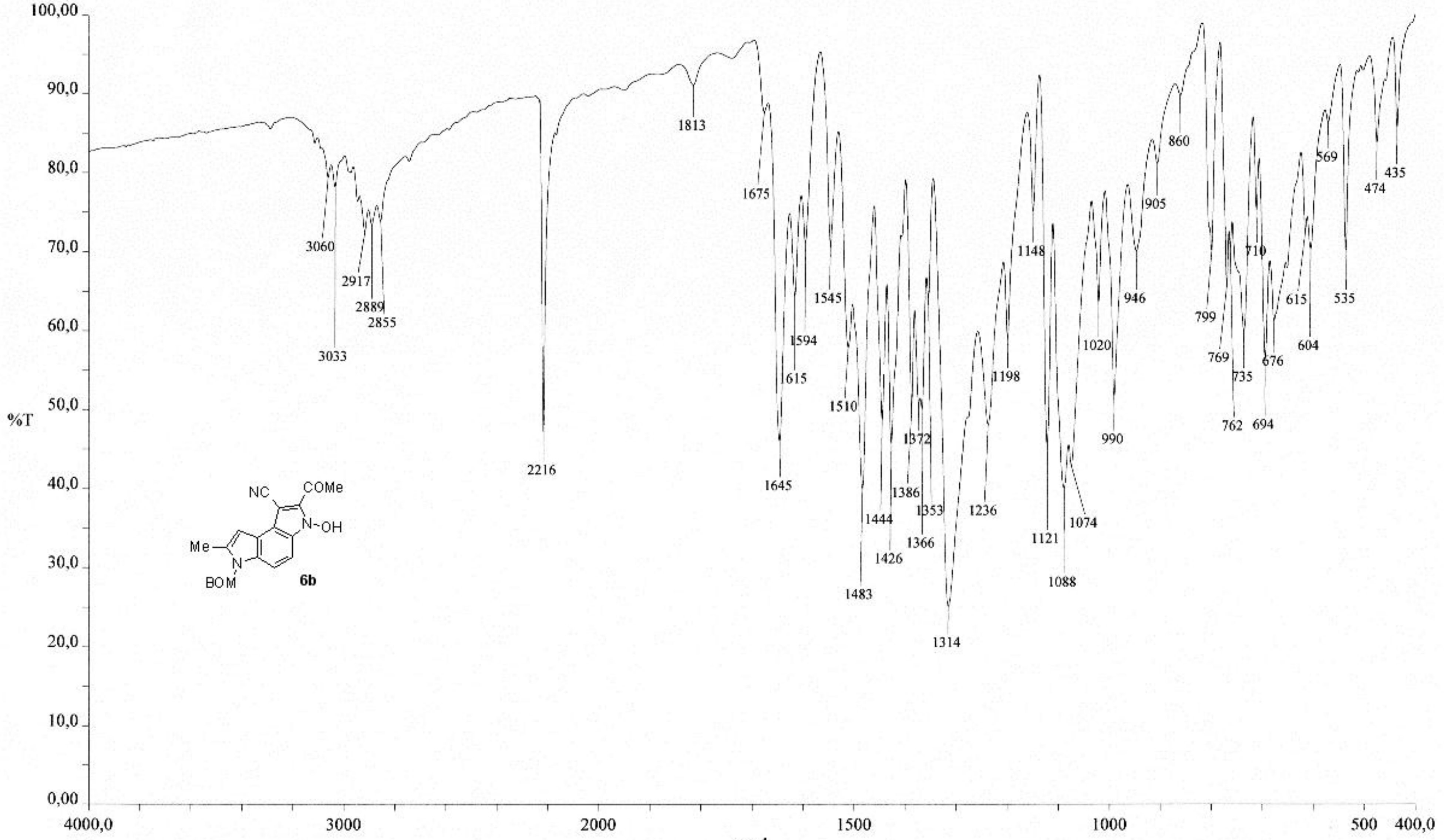

Opis: Pan Adam Trawczyński; ATIN - 52; w KBr

Uwagi:

Numer widma: 12032809.sp

Operator: Alicja Dziedzic

File Name : E:\IChO\Z07_EG\at3475.ms2
Creation Date/Time : 11-11-28 at 12:54:06
File Type : Lo-Res Data - Ctd (Magnet)
File Source : Acquired on MASPEC II system [II32/99D9]
File Title : ATIN-52 (EI 70 eV 33-800)
Operator : Marian Olejnik
Instrument : AMD 604
Notes : A. Trawczyński

SCAN GRAPH. Flagging=Nominal M/z. Highlighting=Base Peak.
Scan 21#2:32. Entries=413. Base M/z=91.2. 100% Int.=27.7504. Temp =316.

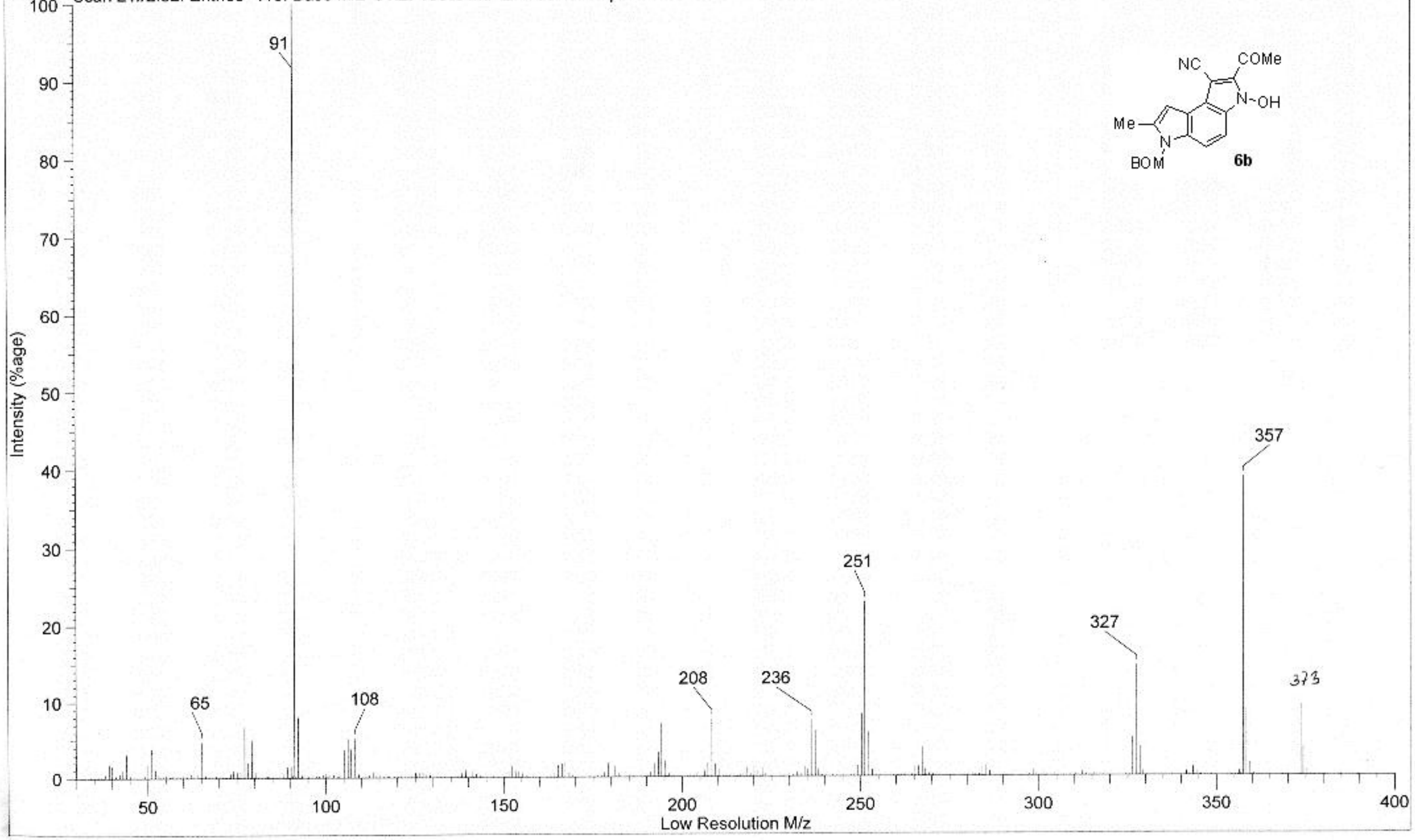

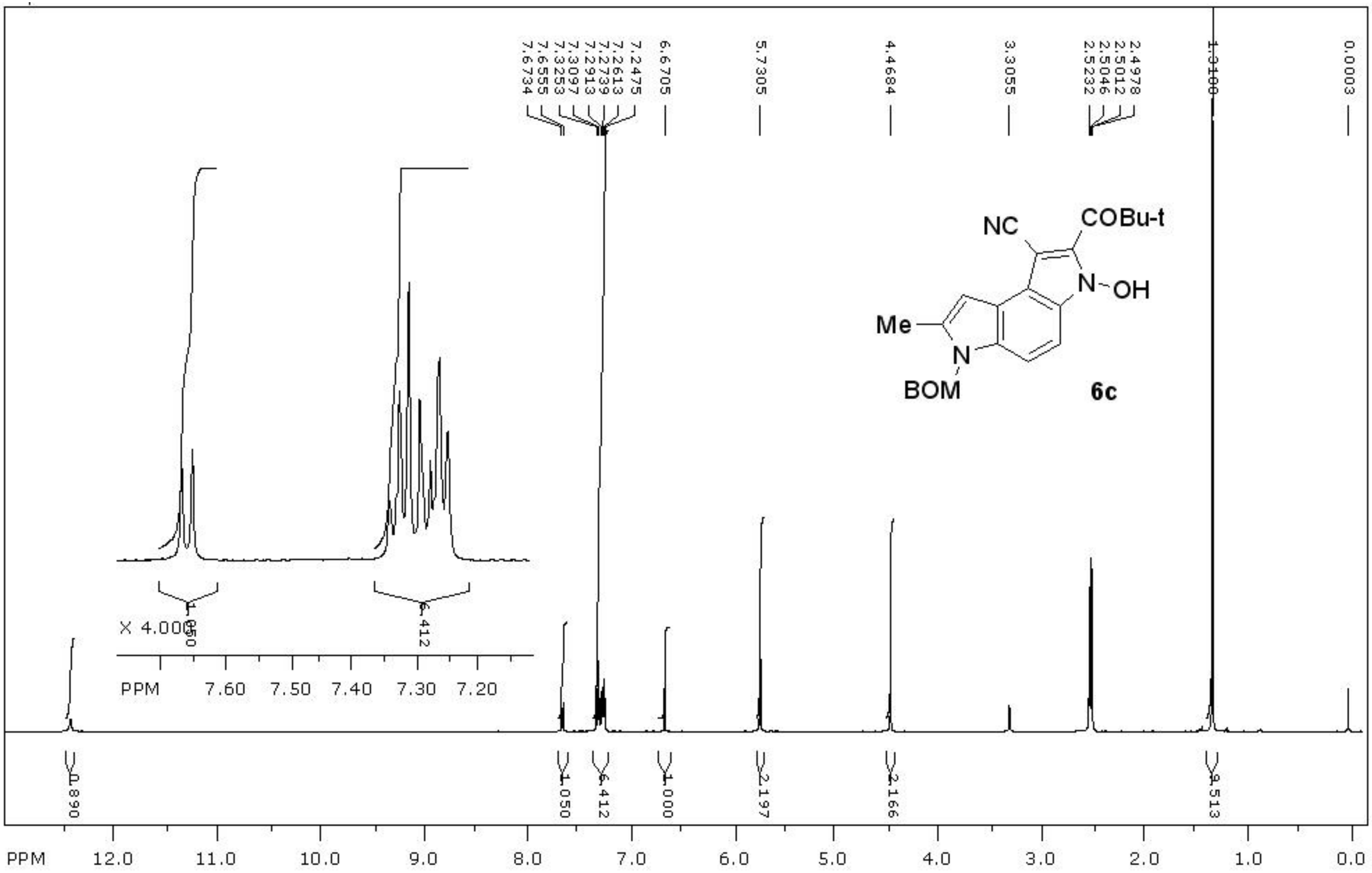

file: ...A\NMR\PIROLOINDOLE\ATIN-47-W\1\fid expt: <zg>
transmitter freq.: 500.133089 MHz
time domain size: 65536 points
width: 10330.58 Hz = 20.6557 ppm = 0.157632 Hz/pt
number of scans: 32

freq. of 0 ppm: 500.130004 MHz
processed size: 32768 complex points
LB: 0.000 GF: 0.0000
Hz/cm: 265.268 ppm/cm: 0.53039

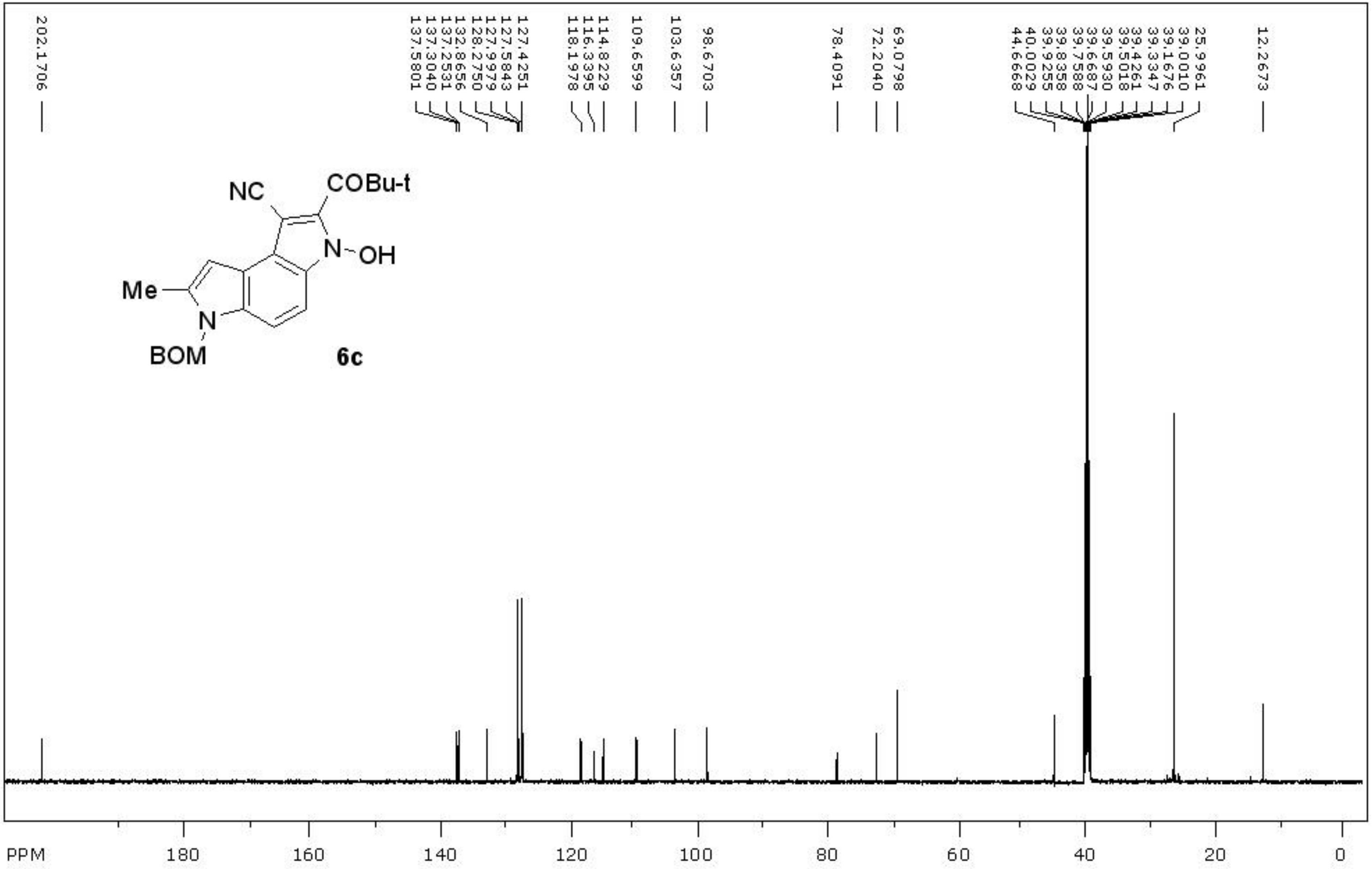

file: ...INDOLE\ATIN-47\ATIN-47-C13.fid\fid block # 1 expt: "s2pul"
transmitter freq.: 125.697101 MHz
time domain size: 80128 points
width: 32051.28 Hz = 254.9882 ppm = 0.400001 Hz/pt
number of scans: 1248

freq. of 0 ppm: 125.683334 MHz
processed size: 131072 complex points
LB: 1.000 GF: 0.0000
Hz/cm: 1063.528 ppm/cm: 8.46104

PE SPECTRUM 2000 IChO PAN Zespół Spektroskopii Optycznej Data: 2012-03-28

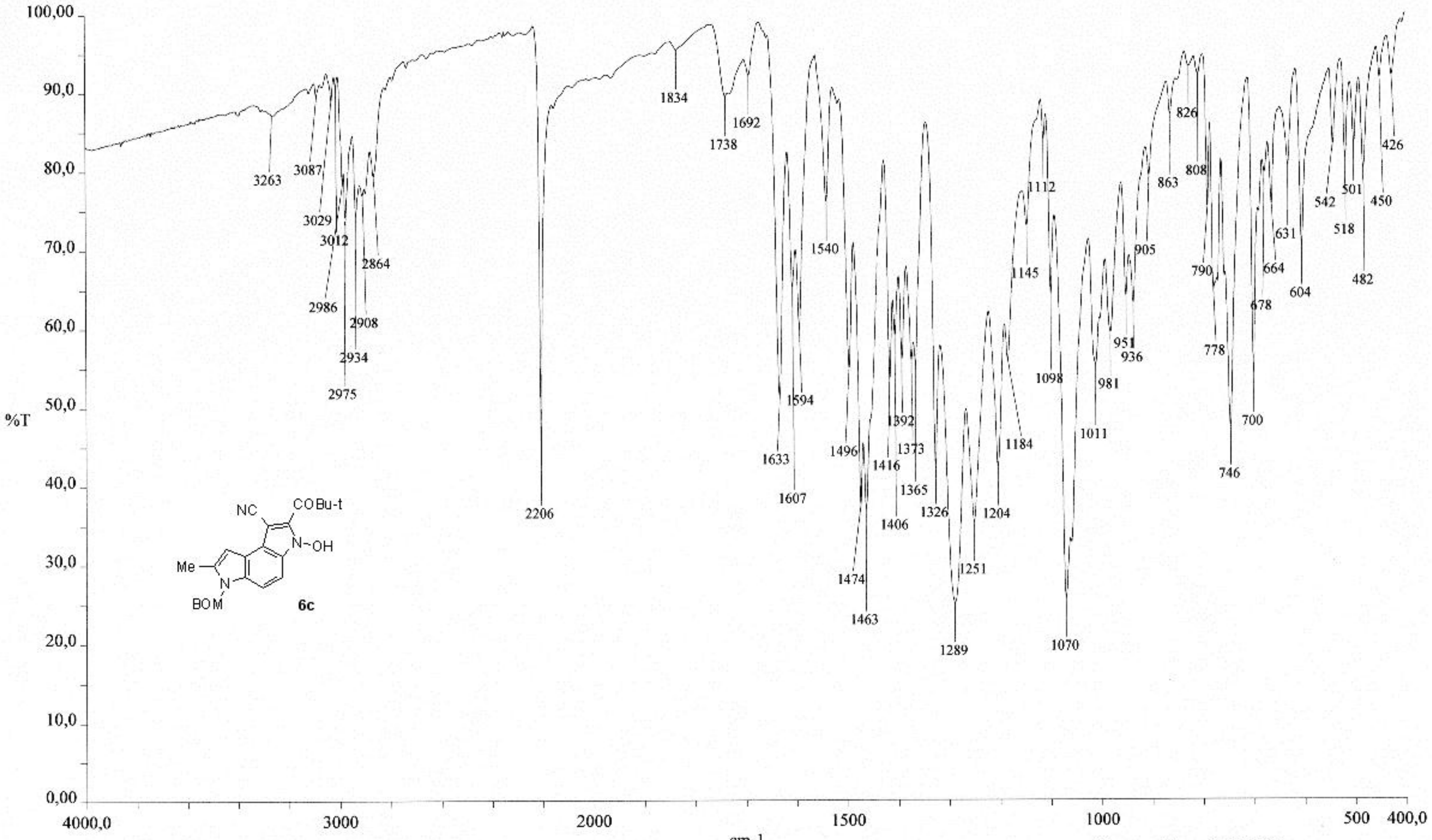

Opis: Pan Adam Trawczyński: ATIN - 47; w KBr

Uwagi:

Numer widma: 12032808.sp

Operator: Alicja Dziedzic

File Name	:	E:\IChO\Z07_EG\at3209.ms2
Creation Date/Time	:	11-10-27 at 15:09:09
File Type	:	Lo-Res Data - Ctd (Magnet)
File Source	:	Acquired on MASPEC II system [II32/99D9]
File Title	:	ATIN-47 (EI 70 eV 33-800)
Operator	:	Marian Olejnik
Instrument	:	AMD 604
Notes	:	A. Trawczyński

SCAN GRAPH. Flagging=Nominal M/z. Highlighting=Base Peak.
Scan 35#4:13. Entries=609. Base M/z=91.1. 100% Int.=43.1616. Temp =315.

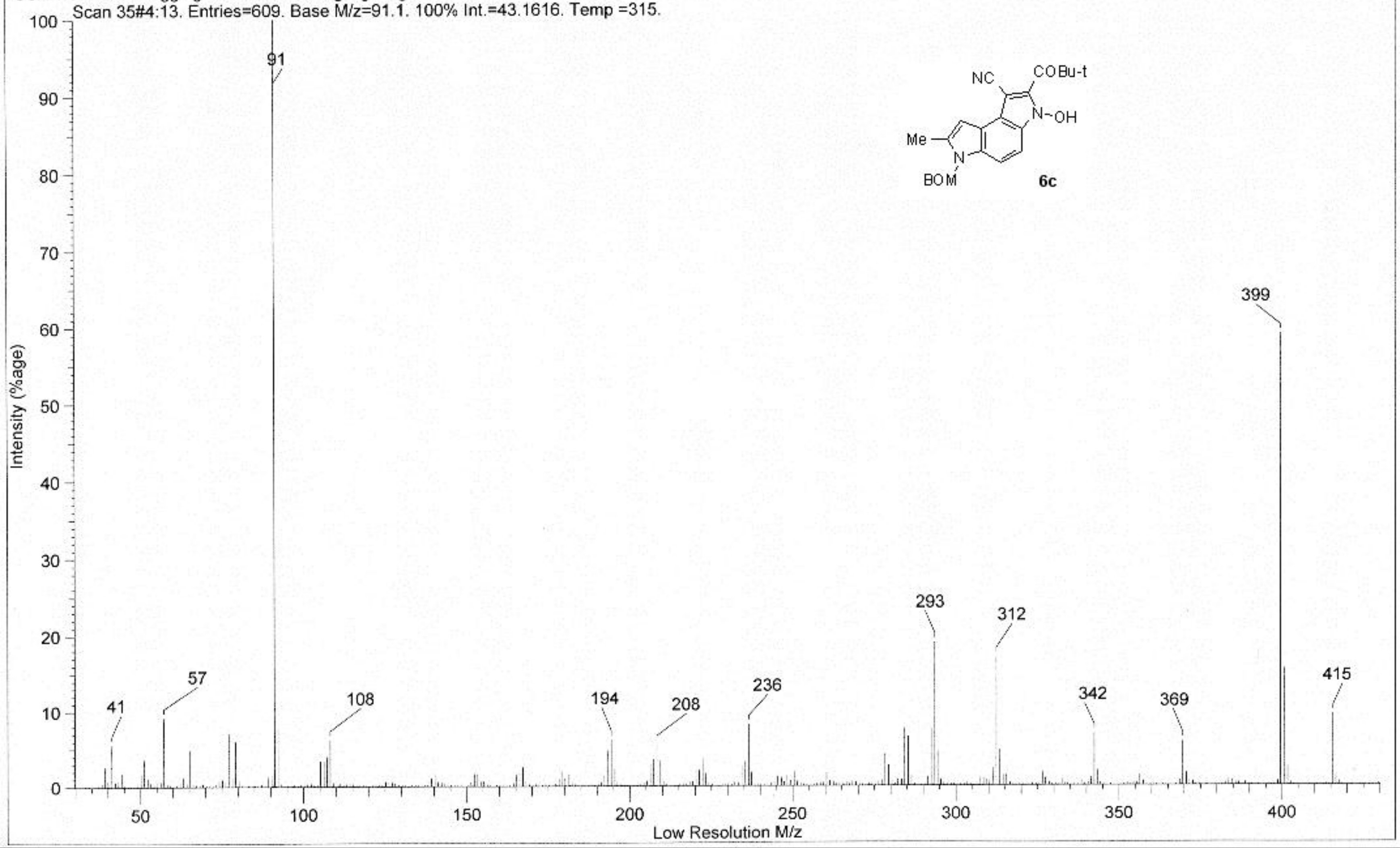

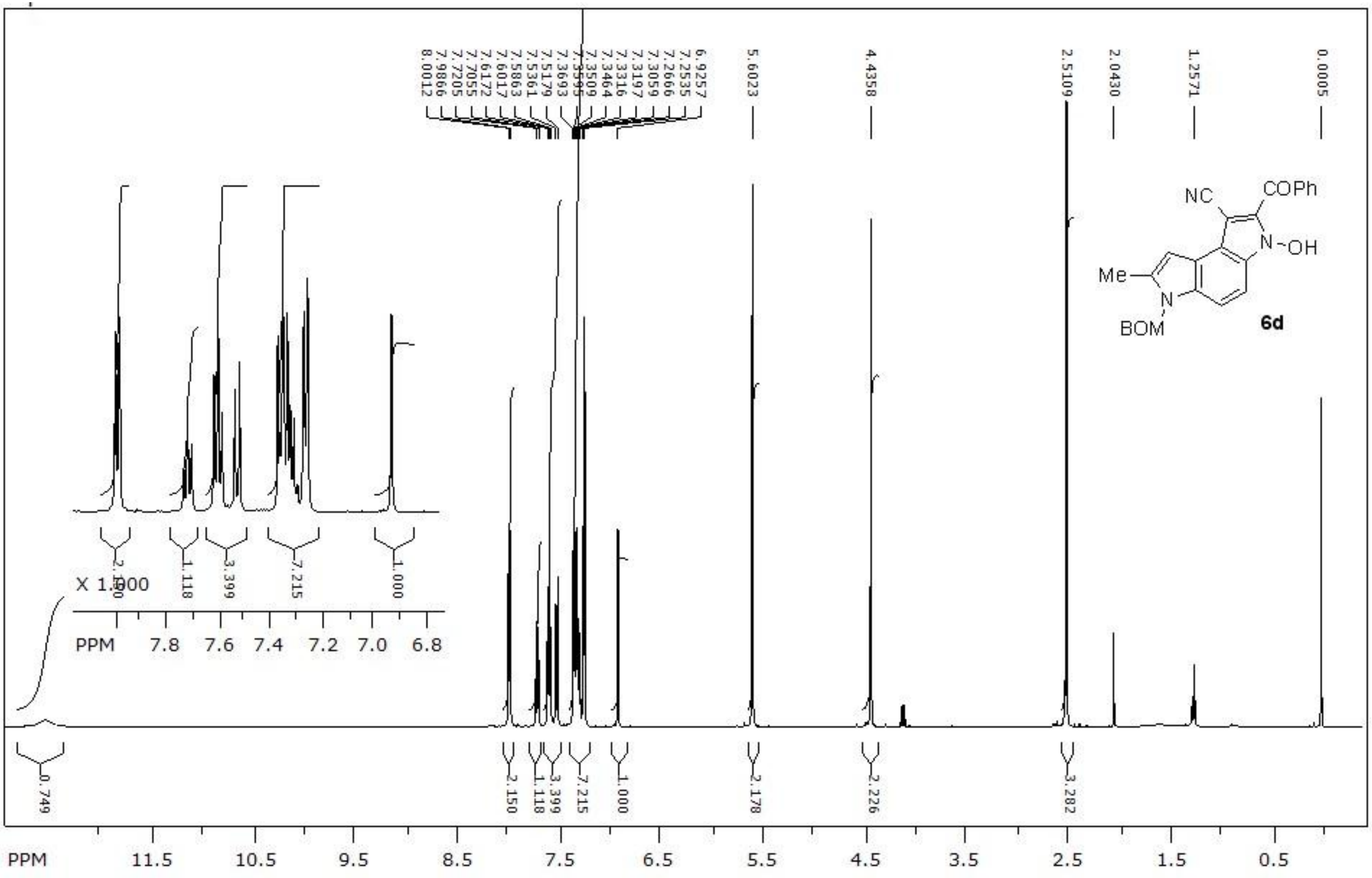

file: D:\NMR\PIROLOINDOLE\ATIN-69\1\fid expt: <zg>
transmitter freq.: 500.133089 MHz
time domain size: 65536 points
width: 10330.58 Hz = 20.6557 ppm = 0.157632 Hz/pt
number of scans: 32

freq. of 0 ppm: 500.130017 MHz
processed size: 32768 complex points
LB: 0.000 GF: 0.0000
Hz/cm: 267.326 ppm/cm: 0.53451

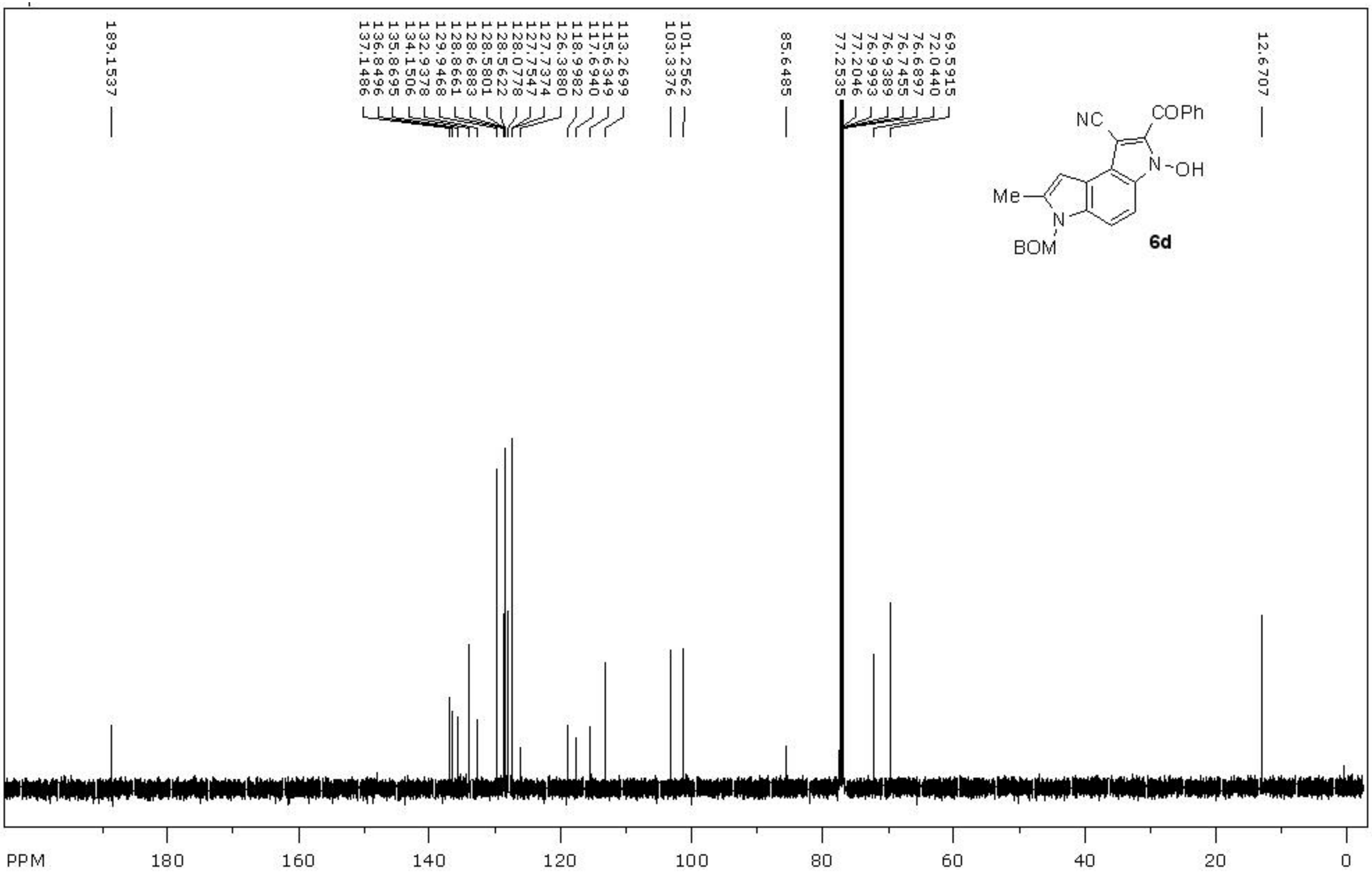

file: ...MIA\NMR\PIROLOINDOLE\ATIN-69\2\fid expt: <zgpg>
transmitter freq.: 125.770364 MHz
time domain size: 65536 points
width: 32679.74 Hz = 259.8366 ppm = 0.498653 Hz/pt
number of scans: 840

freq. of 0 ppm: 125.757795 MHz
processed size: 262144 complex points
LB: 0.500 GF: 0.0000
Hz/cm: 1048.935 ppm/cm: 8.34008

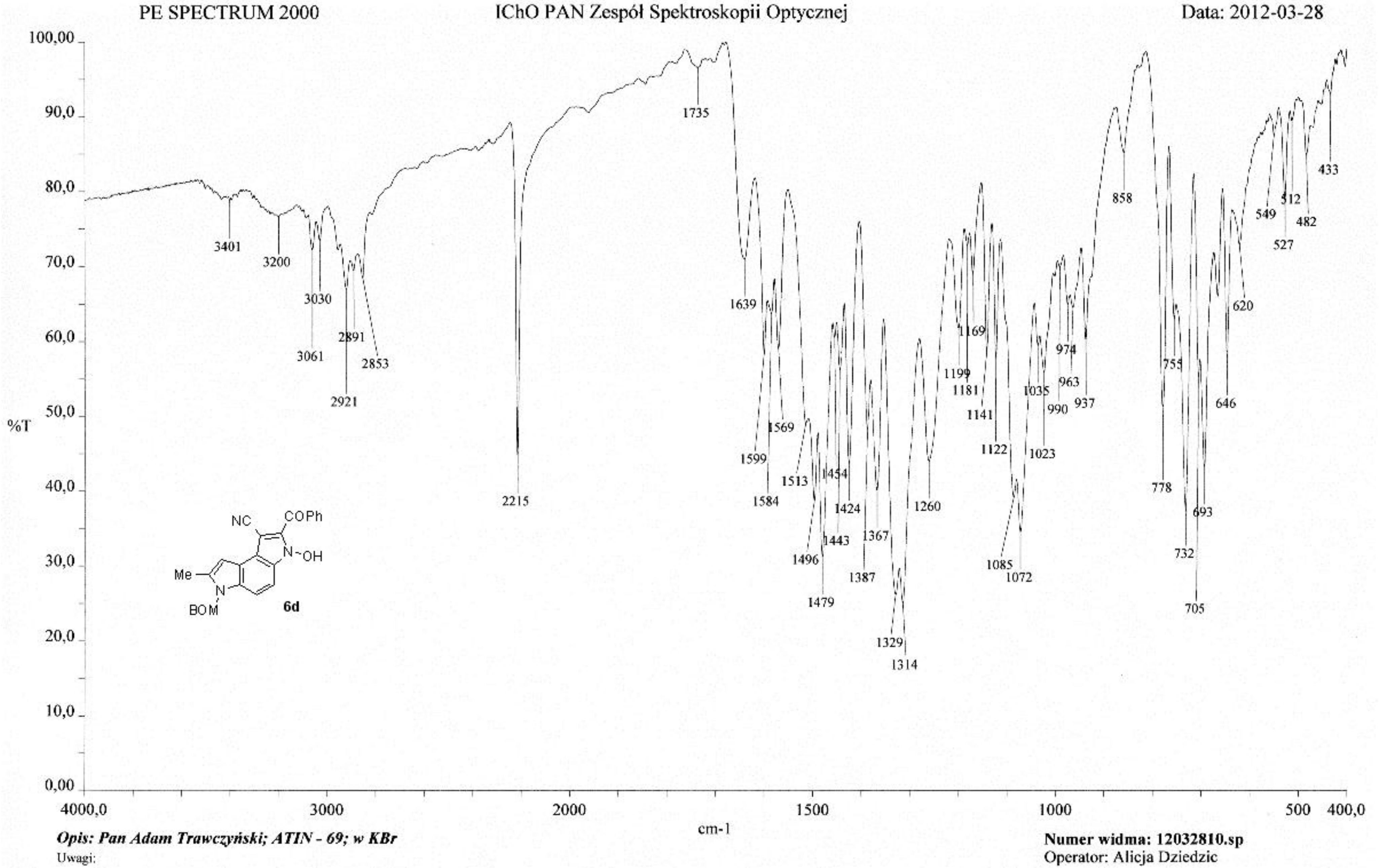
PE SPECTRUM 2000
IChO PAN Zespół Spektroskopii Optycznej
Data: 2012-03-28
%T
cm-1
NC
COPh
N
OH
Me
N
BOM
6d
3401
3200
3030
3061
2921
2891
2853
2215
1735
1639
1599
1584
1569
1513
1496
1479
1454
1443
1424
1387
1367
1329
1314
1260
1199
1181
1169
1141
1122
1085
1072
1035
1023
990
974
963
937
858
778
755
732
705
693
646
620
549
527
512
482
433
Opis: Pan Adam Trawczyński; ATIN - 69; w KBr
Uwagi:
Numer widma: 12032810.sp
Operator: Alicja Dziedzic

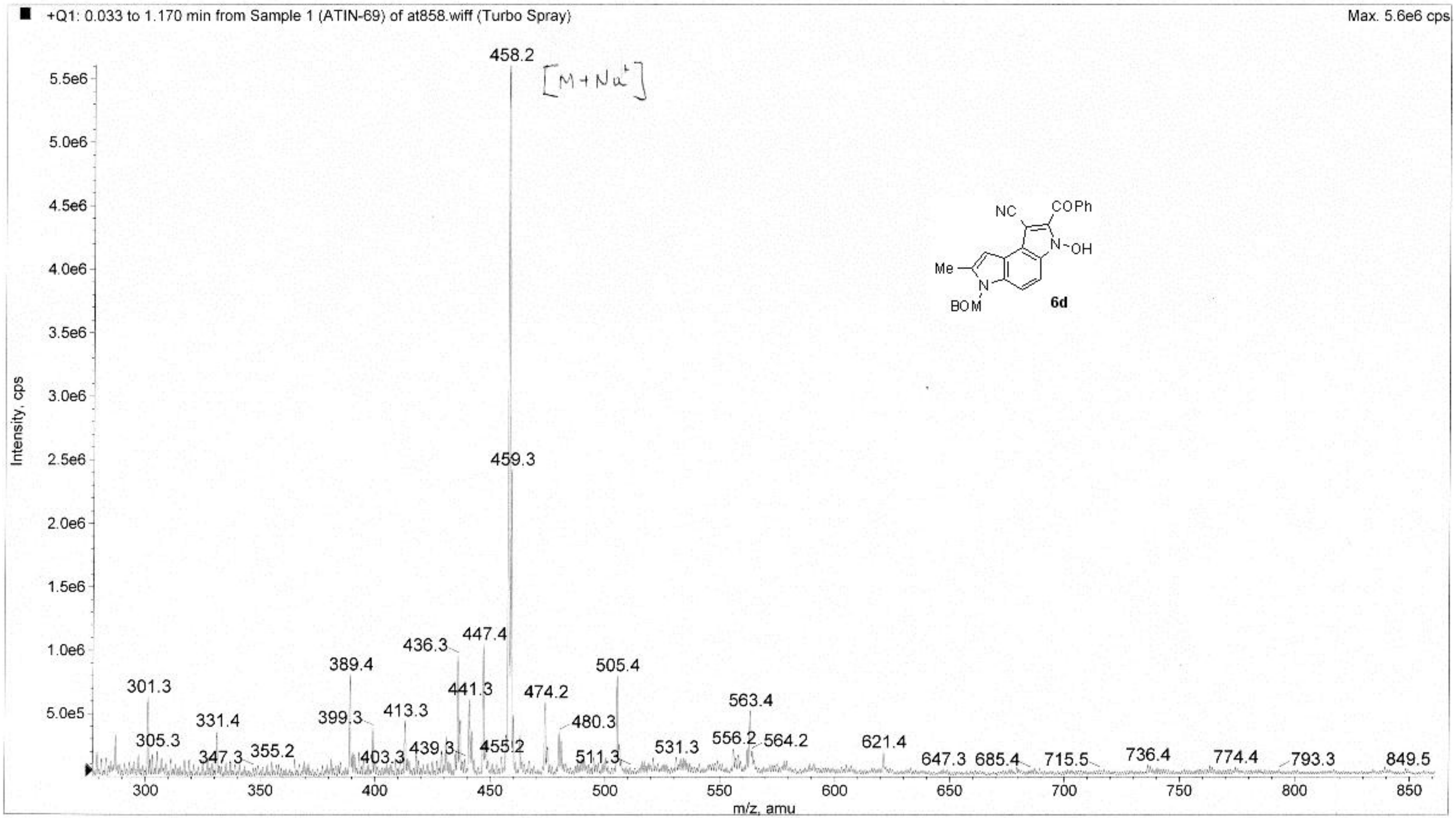
+Q1: 0.033 to 1.170 min from Sample 1 (ATIN-69) of at858.wiff (Turbo Spray)
Max. 5.6e6 cps
458.2
[M+Na+]
459.3
447.4
436.3
389.4
505.4
441.3
474.2
301.3
563.4
413.3
399.3
331.4
480.3
305.3
556.2
564.2
621.4
347.3
355.2
403.3
439.3
455.2
511.3
531.3
647.3
685.4
715.5
736.4
774.4
793.3
849.5
Intensity, cps
m/z, amu
NC
COPh
N-OH
Me
N
BOM
6d

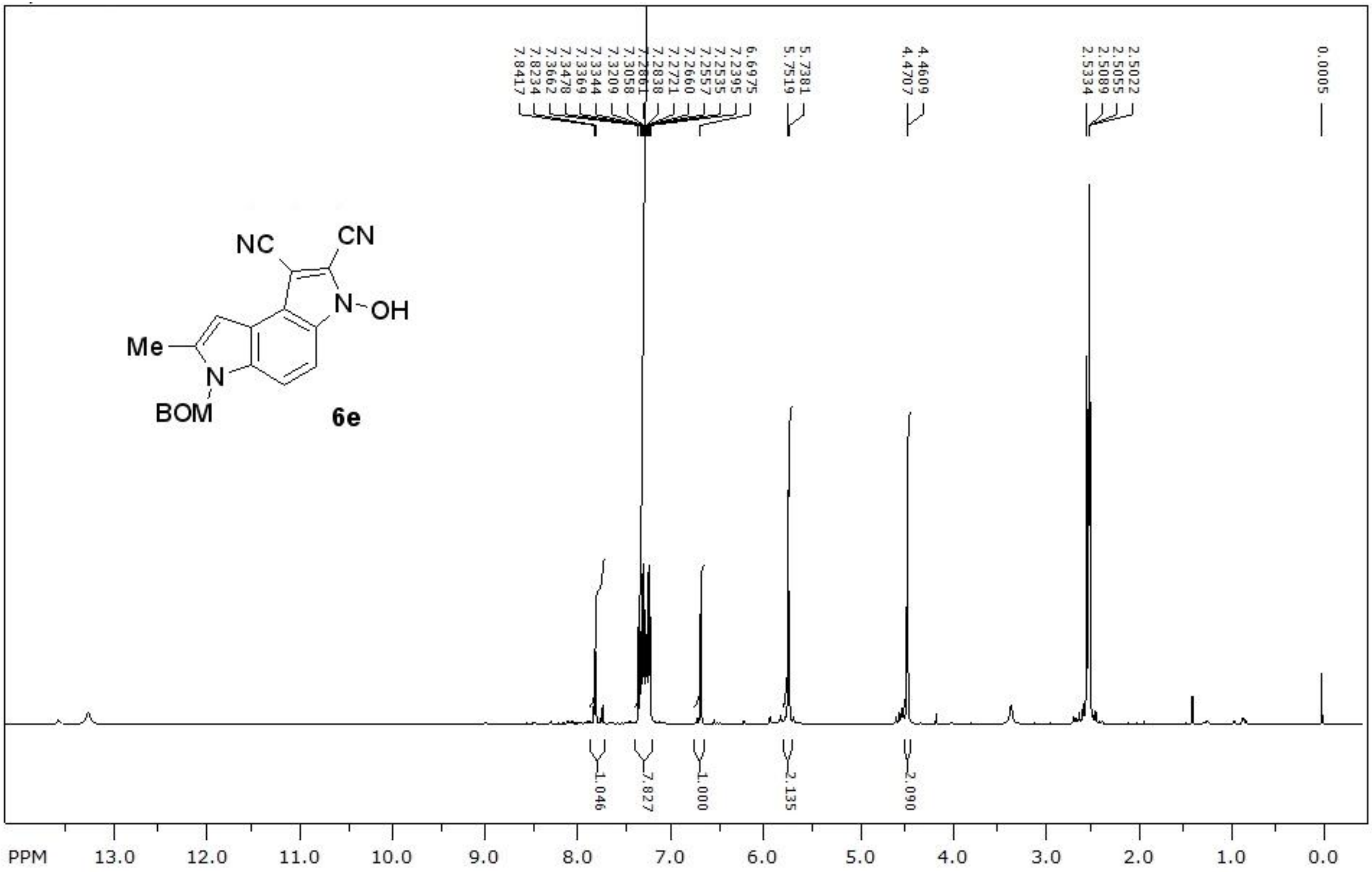

file: D:\NMR\PIROLOINDOLE\ATIN-74\1\fid expt: <zg>
transmitter freq.: 500.133089 MHz
time domain size: 65536 points
width: 10330.58 Hz = 20.6557 ppm = 0.157632 Hz/pt
number of scans: 32

freq. of 0 ppm: 500.130003 MHz
processed size: 32768 complex points
LB: 0.000 GF: 0.0000
Hz/cm: 292.674 ppm/cm: 0.58519

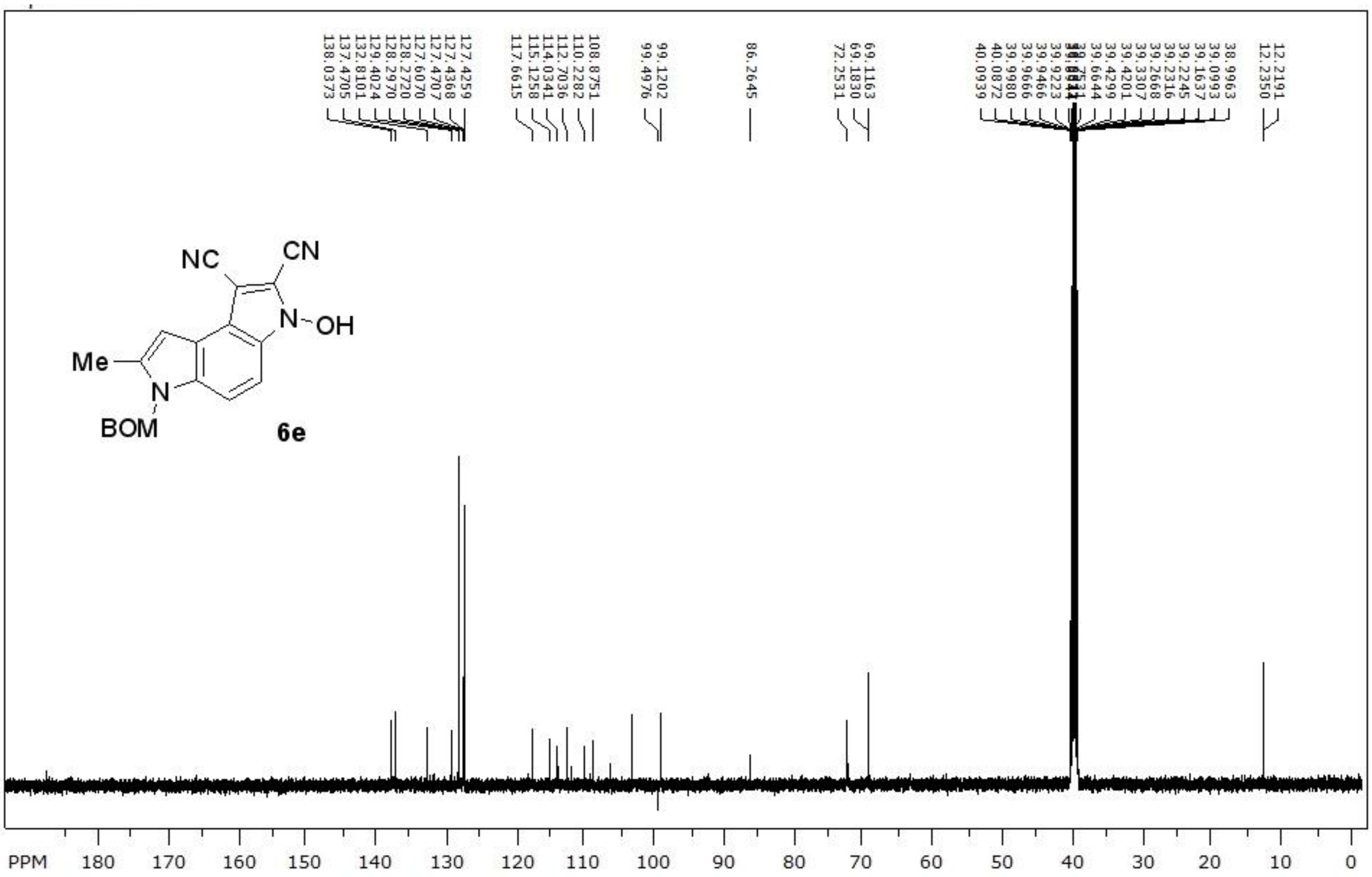

file: D:\NMR\PIROLOINDOLE\ATIN-74\2\fid expt: <zgpg>
transmitter freq.: 125.770364 MHz
time domain size: 65536 points
width: 32679.74 Hz = 259.8366 ppm = 0.498653 Hz/pt
number of scans: 1880

freq. of 0 ppm: 125.757852 MHz
processed size: 262144 complex points
LB: 0.500 GF: 0.0000
Hz/cm: 984.463 ppm/cm: 7.82747

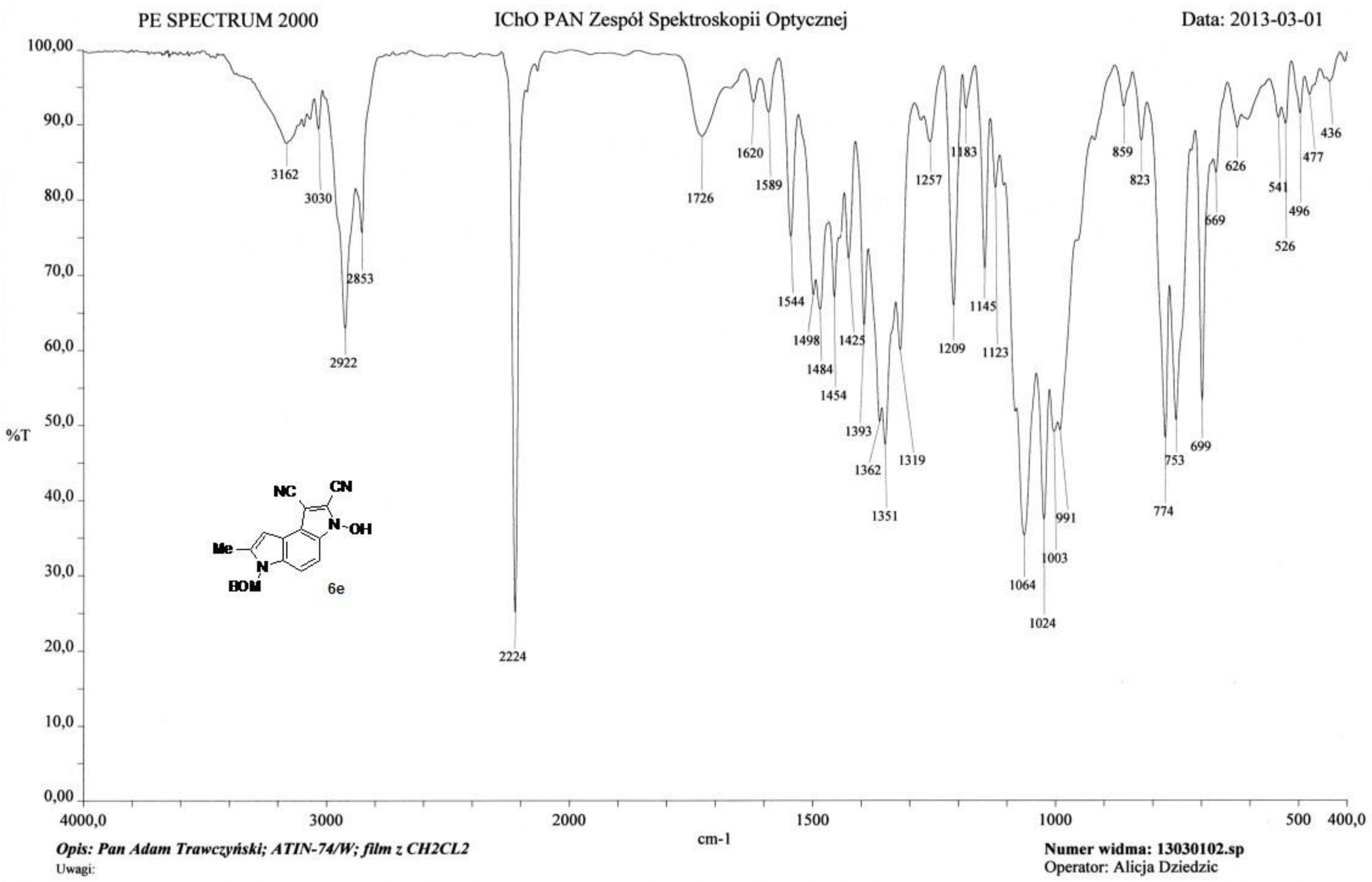
PE SPECTRUM 2000
IChO PAN Zespół Spektroskopii Optycznej
Data: 2013-03-01
%T
cm-1
6e
Opis: Pan Adam Trawczyński; ATIN-74/W; film z CH2CL2
Uwagi:
Numer widma: 13030102.sp
Operator: Alicja Dziedzic

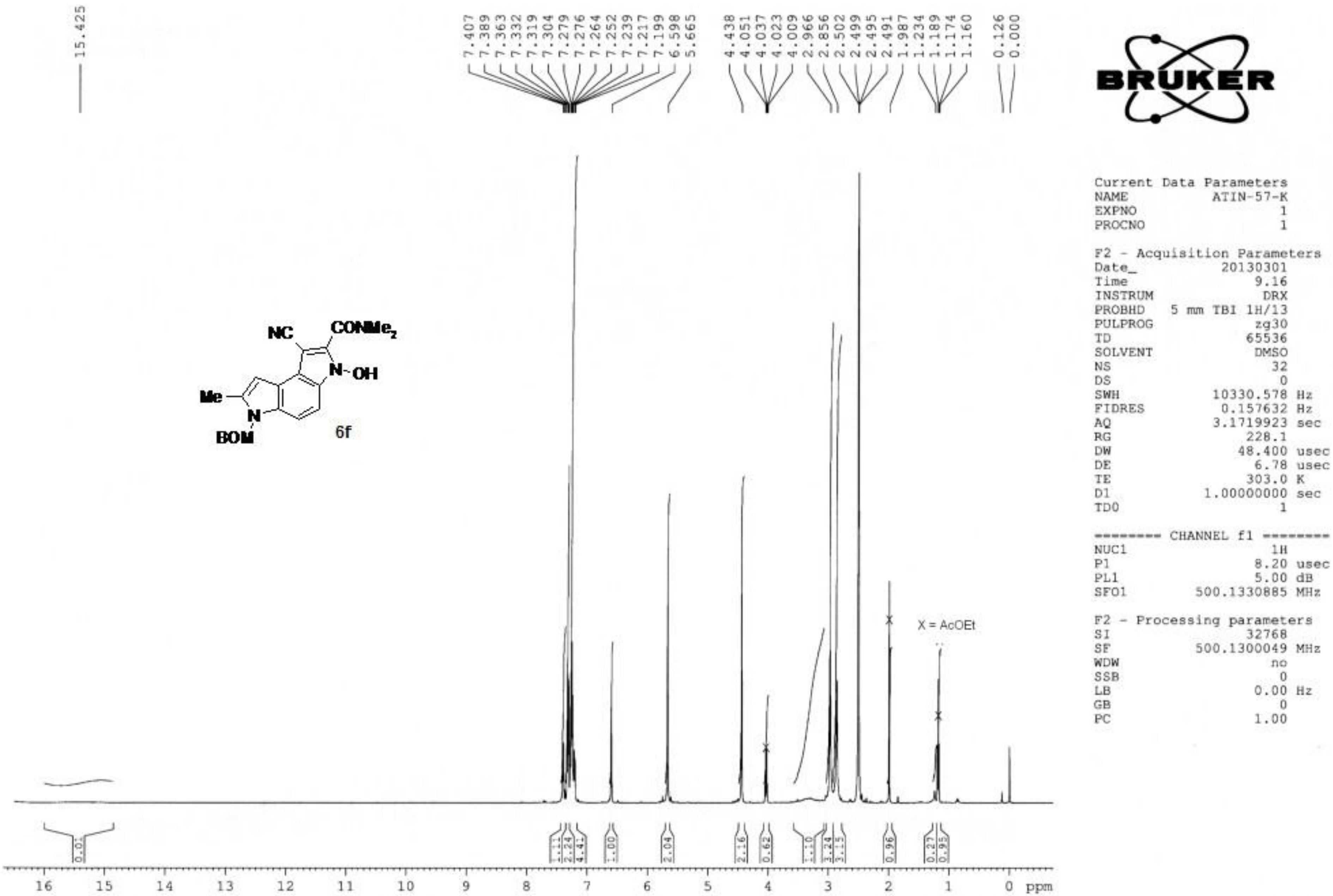
15.425
7.407 7.389 7.363 7.332 7.319 7.304 7.279 7.276 7.264 7.252 7.239 7.217 7.199 6.598 5.665
4.438 4.051 4.037 4.023 4.009 2.966 2.856 2.502 2.499 2.495 2.491 1.987 1.234 1.189 1.174 1.160
0.126 0.000
NC
CONMe2
N-OH
Me
N
BOM
6f
X = AcOEt
BRUKER
Current Data Parameters
NAME ATIN-57-K
EXPNO 1
PROCNO 1
F2 - Acquisition Parameters
Date_ 20130301
Time 9.16
INSTRUM DRX
PROBHD 5 mm TBI 1H/13
PULPROG zg30
TD 65536
SOLVENT DMSO
NS 32
DS 0
SWH 10330.578 Hz
FIDRES 0.157632 Hz
AQ 3.1719923 sec
RG 228.1
DW 48.400 usec
DE 6.78 usec
TE 303.0 K
D1 1.00000000 sec
TD0 1
======== CHANNEL f1 ========
NUC1 1H
P1 8.20 usec
PL1 5.00 dB
SFO1 500.1330885 MHz
F2 - Processing parameters
SI 32768
SF 500.1300049 MHz
WDW no
SSB 0
LB 0.00 Hz
GB 0
PC 1.00
0.01
1.11 2.24 4.41
1.00
2.04
2.16
0.62
1.10
3.24 3.15
0.96
0.27 0.95
16 15 14 13 12 11 10 9 8 7 6 5 4 3 2 1 0 ppm

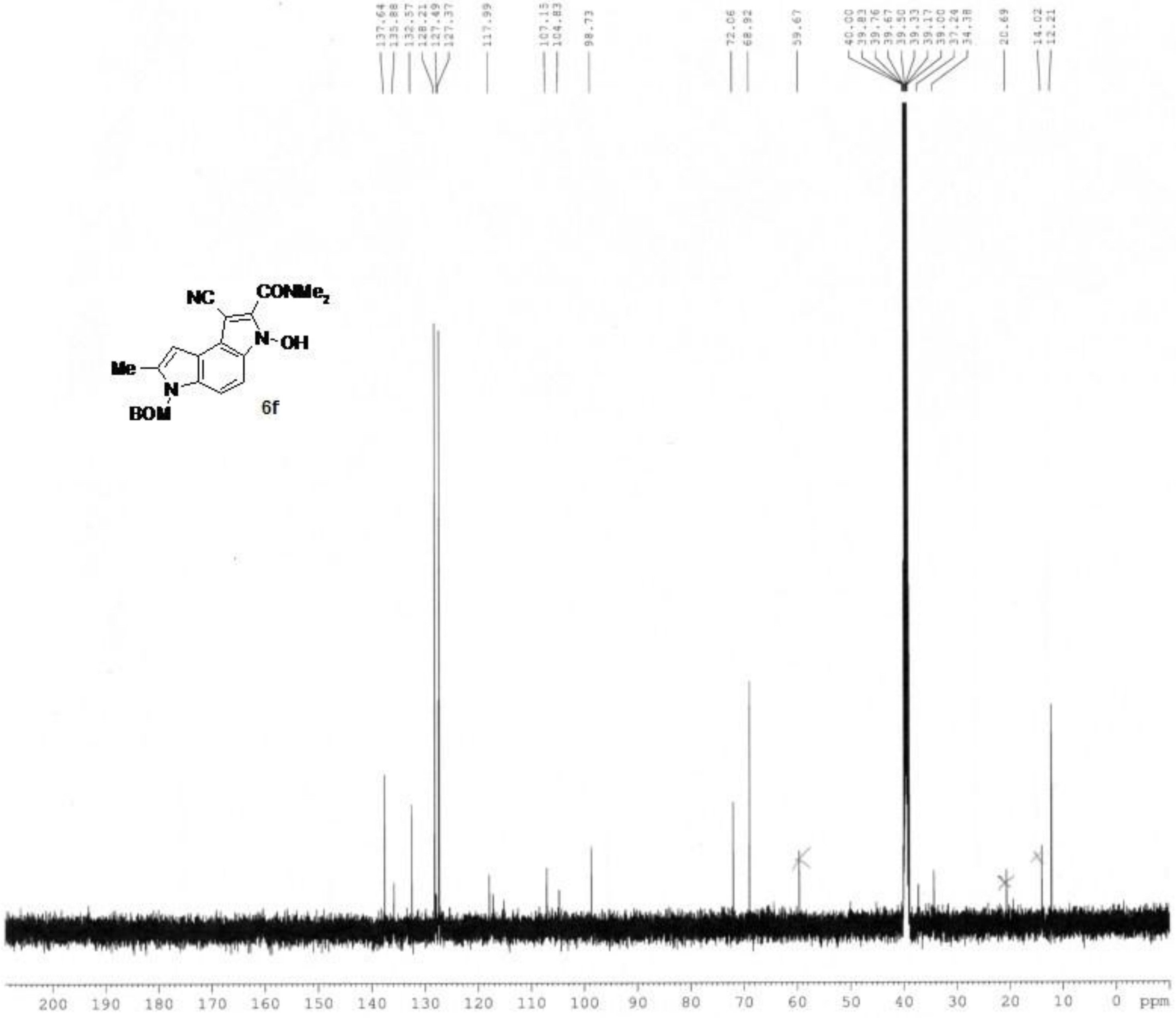

6f

BRUKER

Current Data Parameters
NAME ATIN-57-K
EXPNO 2
PROCNO 1

F2 - Acquisition Parameters
Date_ 20130301
Time 9.29
INSTRUM DRX
PROBHD 5 mm TBI 1H/13
PULPROG zgpg
TD 65536
SOLVENT DMSO
NS 2100 !
DS 4
SWH 32679.738 Hz
FIDRES 0.498653 Hz
AQ 1.0027508 sec
RG 32768
DW 15.300 usec
DE 7.10 usec
TE 303.0 K
D1 1.00000000 sec
d11 0.03000000 sec
DELTA 0.89999998 sec
TD0 1

======== CHANNEL f1 ========
NUC1 13C
P1 5.00 usec
PL1 -3.00 dB
SFO1 125.7703643 MHz

======== CHANNEL f2 ========
CPDPRG2 waltz16
NUC2 1H
PCPD2 98.00 usec
PL2 3.00 dB
PL12 23.00 dB
PL13 32.00 dB
SFO2 500.1320005 MHz

F1 - Acquisition parameters
ND0 1
TD 128
SFO1 500.132 MHz
FIDRES 7.812500 Hz
SW 1.999 ppm
FnMODE QF

F2 - Processing parameters
SI 262144
SF 125.7578564 MHz
WDW EM
SSB 0
LB 0.50 Hz
GB 0
PC 1.40

F1 - Processing parameters
SI 1024
MC2 QF
SF 500.1300000 MHz
WDW SINE
SSB 0
LB 0.30 Hz
GB 0.1

PE SPECTRUM 2000 ICHO PAN Zespół Spektroskopii Optycznej Data: 2013-03-01

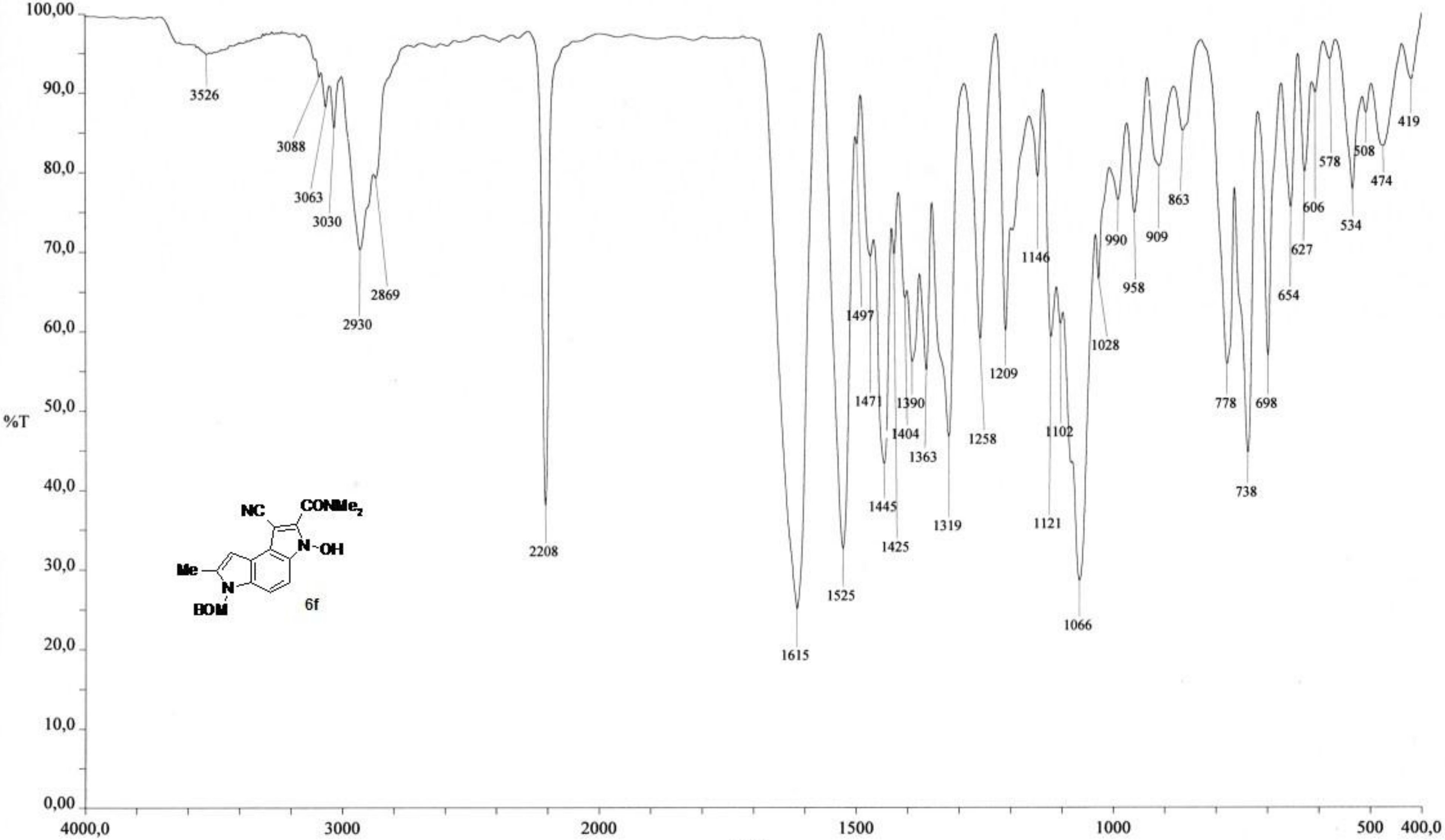

Opis: Pan Adam Trawczyński; ATIN-57/K; film z CH2Cl2

Uwagi:

Numer widma: 13030101.sp

Operator: Alicja Dziedzic

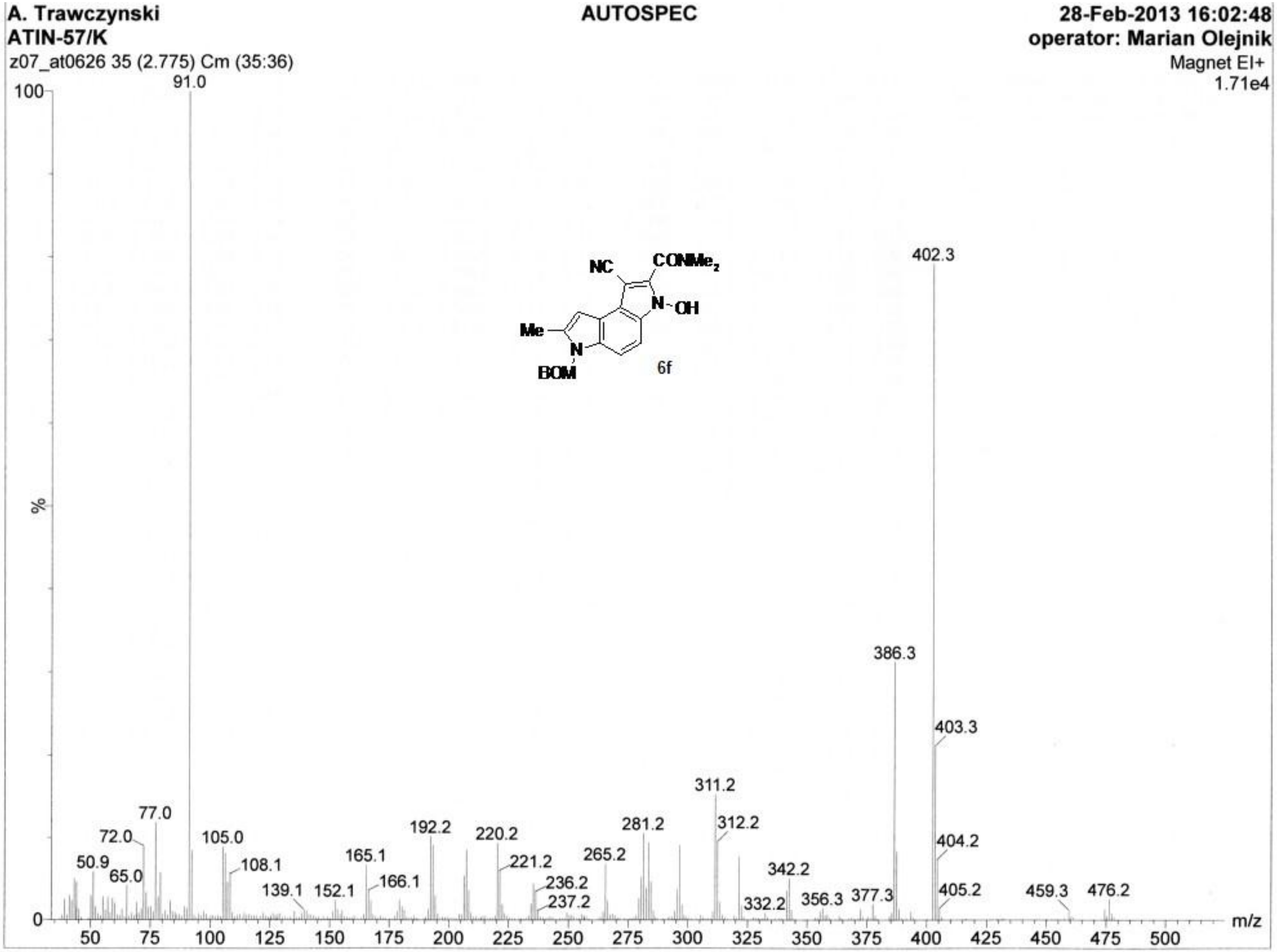
A. Trawczynski
ATIN-57/K
z07_at0626 35 (2.775) Cm (35:36)
AUTOSPEC
28-Feb-2013 16:02:48
operator: Marian Olejnik
Magnet EI+
1.71e4
100
%
0
NC
CONMe2
N-OH
Me
N
BOM
6f
91.0
402.3
386.3
403.3
311.2
312.2
404.2
77.0
72.0
50.9
65.0
105.0
108.1
139.1
152.1
165.1
166.1
192.2
220.2
221.2
236.2
237.2
265.2
281.2
332.2
342.2
356.3
377.3
405.2
459.3
476.2
50
75
100
125
150
175
200
225
250
275
300
325
350
375
400
425
450
475
500
m/z

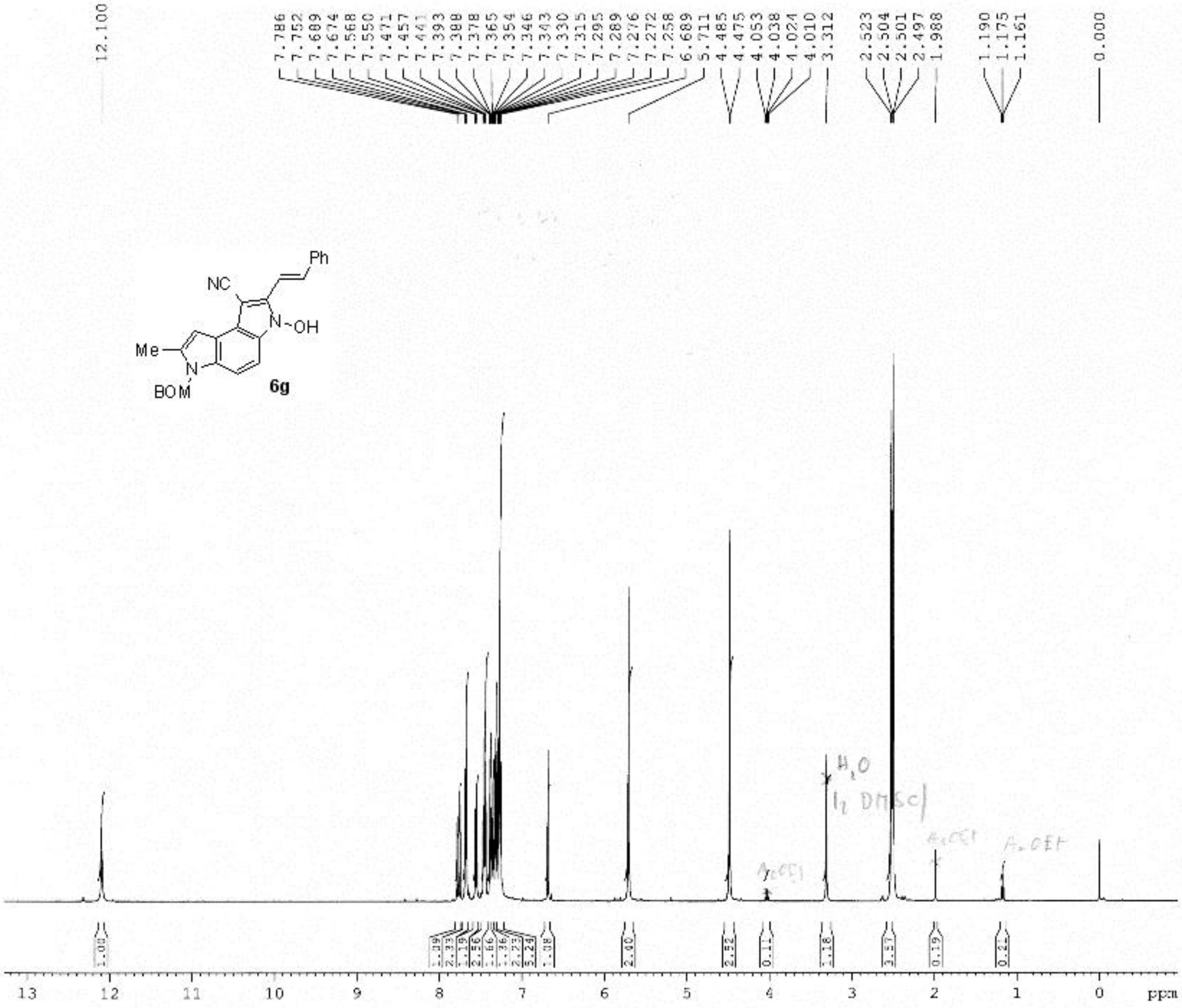

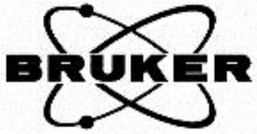

Current Data Parameters
NAME ATIN-59-K
EXPNO 1
PROCNO 1

F2 - Acquisition Parameters
Date_ 20130226
Time 9.17
INSTRUM DRX
PROBHD 5 mm TBI 1H/13
PULPROG zg
TD 65536
SOLVENT DMSO
NS 32
DS 0
SWH 10330.578 Hz
FIDRES 0.157632 Hz
AQ 3.1719923 sec
RG 128
DW 48.400 usec
DE 6.78 usec
TE 303.0 K
D1 1.00000000 sec
TD0 1

======== CHANNEL f1 ========
NUC1 1H
P1 2.50 usec
PL1 0.00 dB
SFO1 500.1330885 MHz

F2 - Processing parameters
SI 32768
SF 500.1300050 MHz
WDW no
SSB 0
LB 0.00 Hz
GB 0
PC 1.00

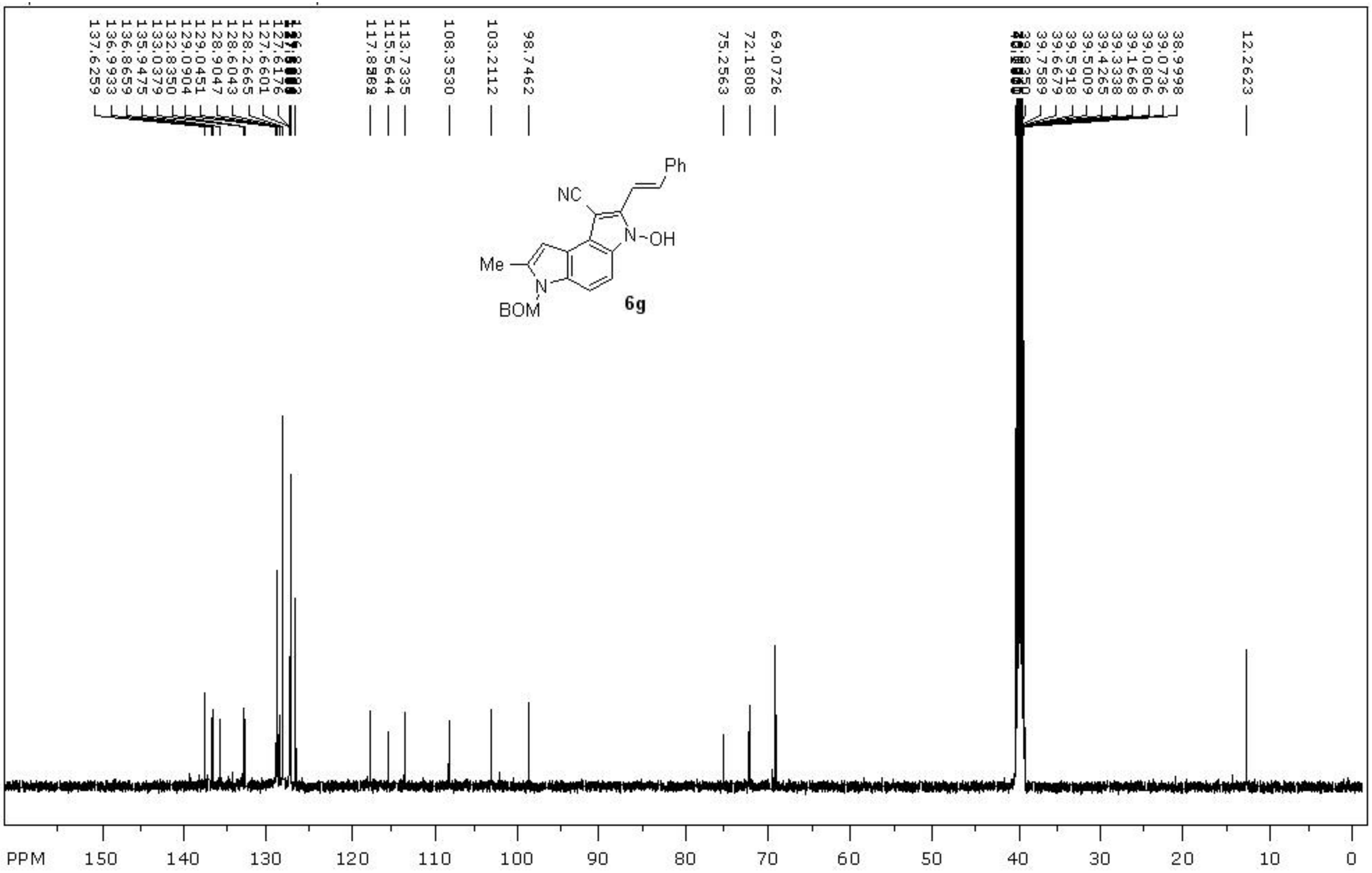

file: ...DOLE\ATIN-592\ATIN-592-C13.fid\fid block# 1 expt: "s2pul"
transmitter freq.: 125.697101 MHz
time domain size: 80128 points
width: 32051.28 Hz = 254.9882 ppm = 0.400001 Hz/pt
number of scans: 3072

freq. of 0 ppm: 125.683336 MHz
processed size: 131072 complex points
LB: 1.000 GF: 0.0000
Hz/cm: 823.010 ppm/cm: 6.54757

PE SPECTRUM 2000 ICHO PAN Zespół Spektroskopii Optycznej Data: 2013-02-26

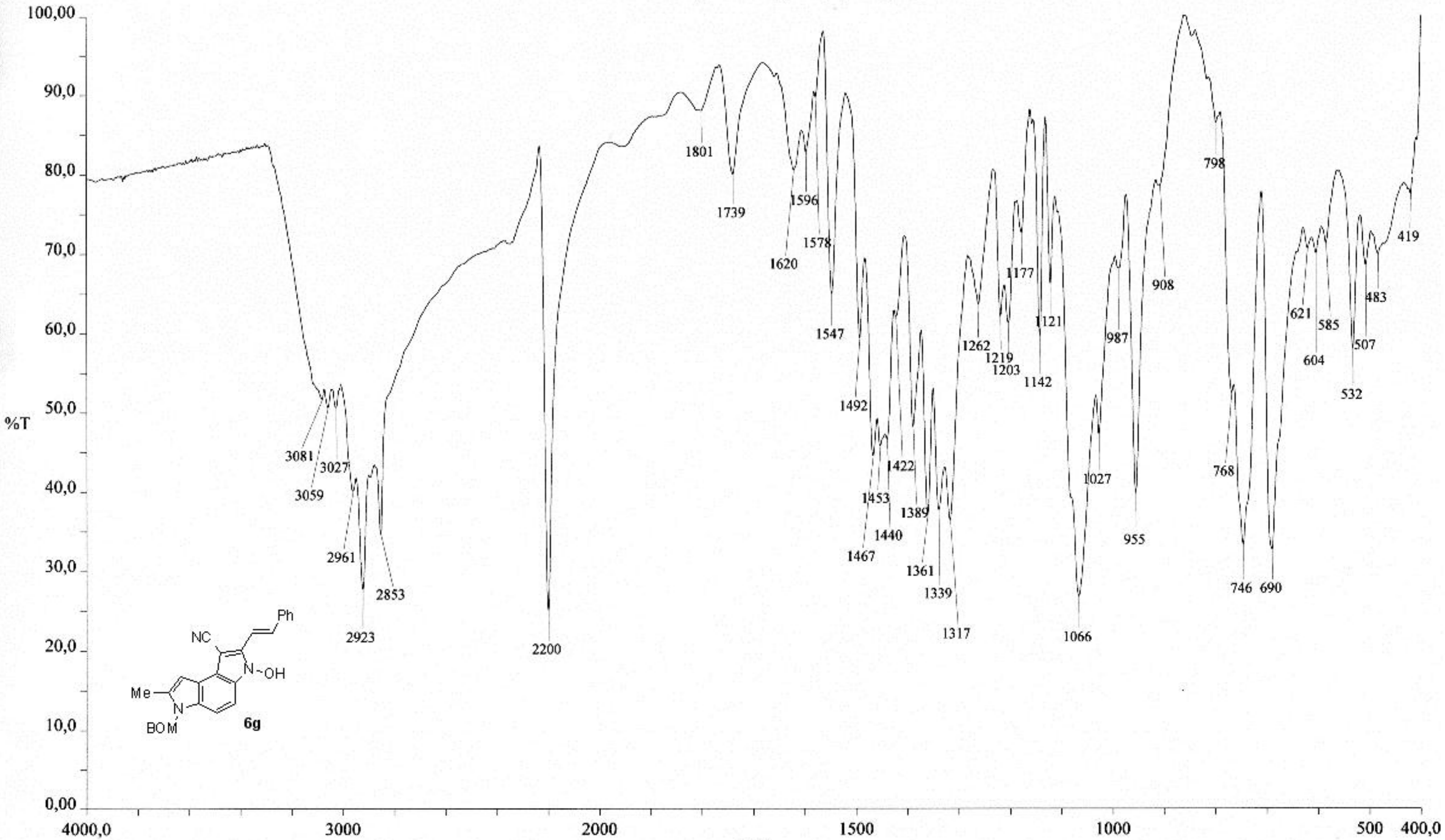

Opis: Pan Adam Trawczyński; ATIN-59/K; w KBr

Uwagi:

Numer widma: 13022601.sp

Operator: Alicja Dziedzic

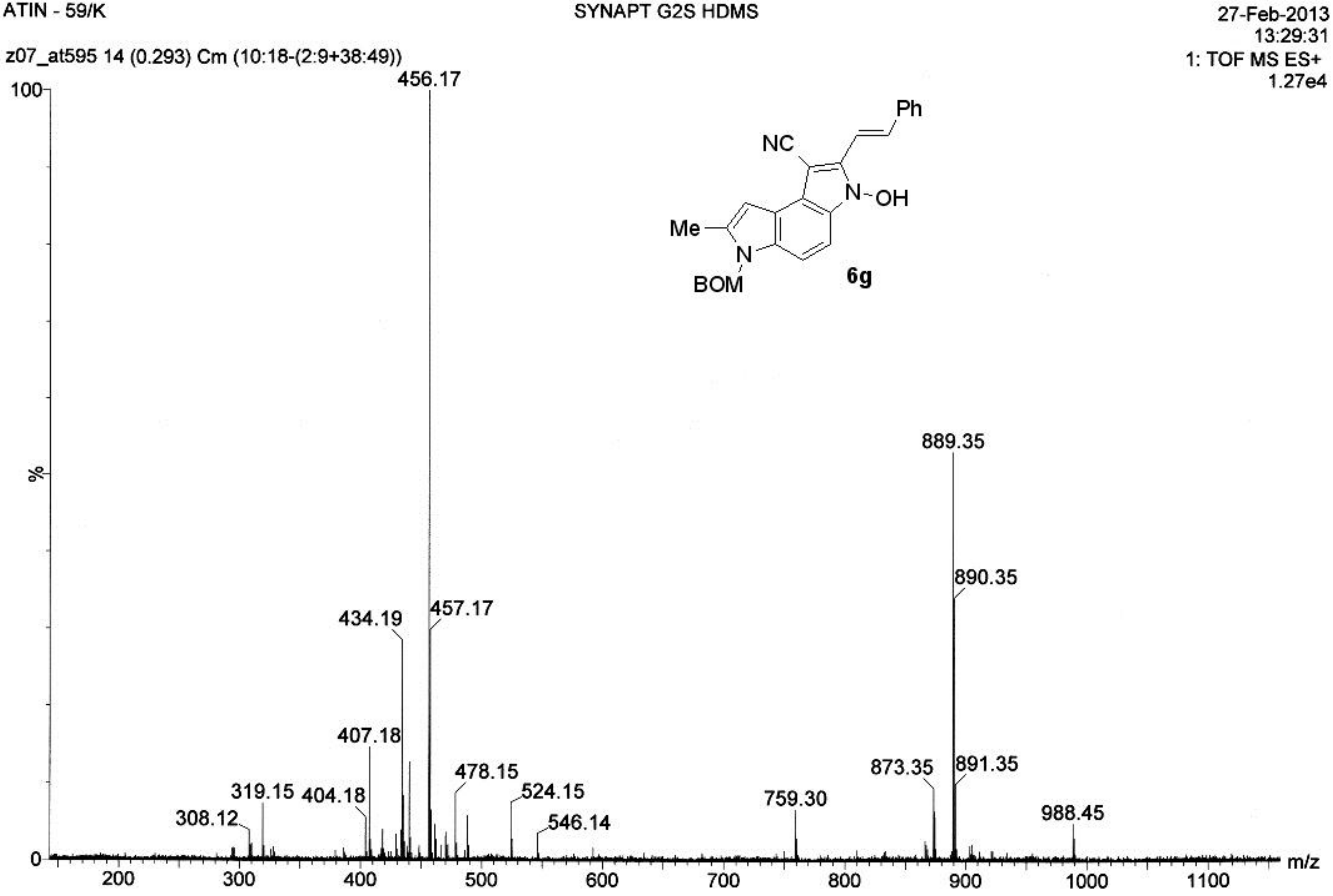
ATIN - 59/K
SYNAPT G2S HDMS
27-Feb-2013
13:29:31
z07_at595 14 (0.293) Cm (10:18-(2:9+38:49))
1: TOF MS ES+
1.27e4
NC
Ph
N-OH
Me
N
BOM
6g
456.17
889.35
890.35
457.17
434.19
407.18
478.15
873.35
891.35
319.15
404.18
524.15
759.30
308.12
546.14
988.45
100
%
0
200
300
400
500
600
700
800
900
1000
1100
m/z

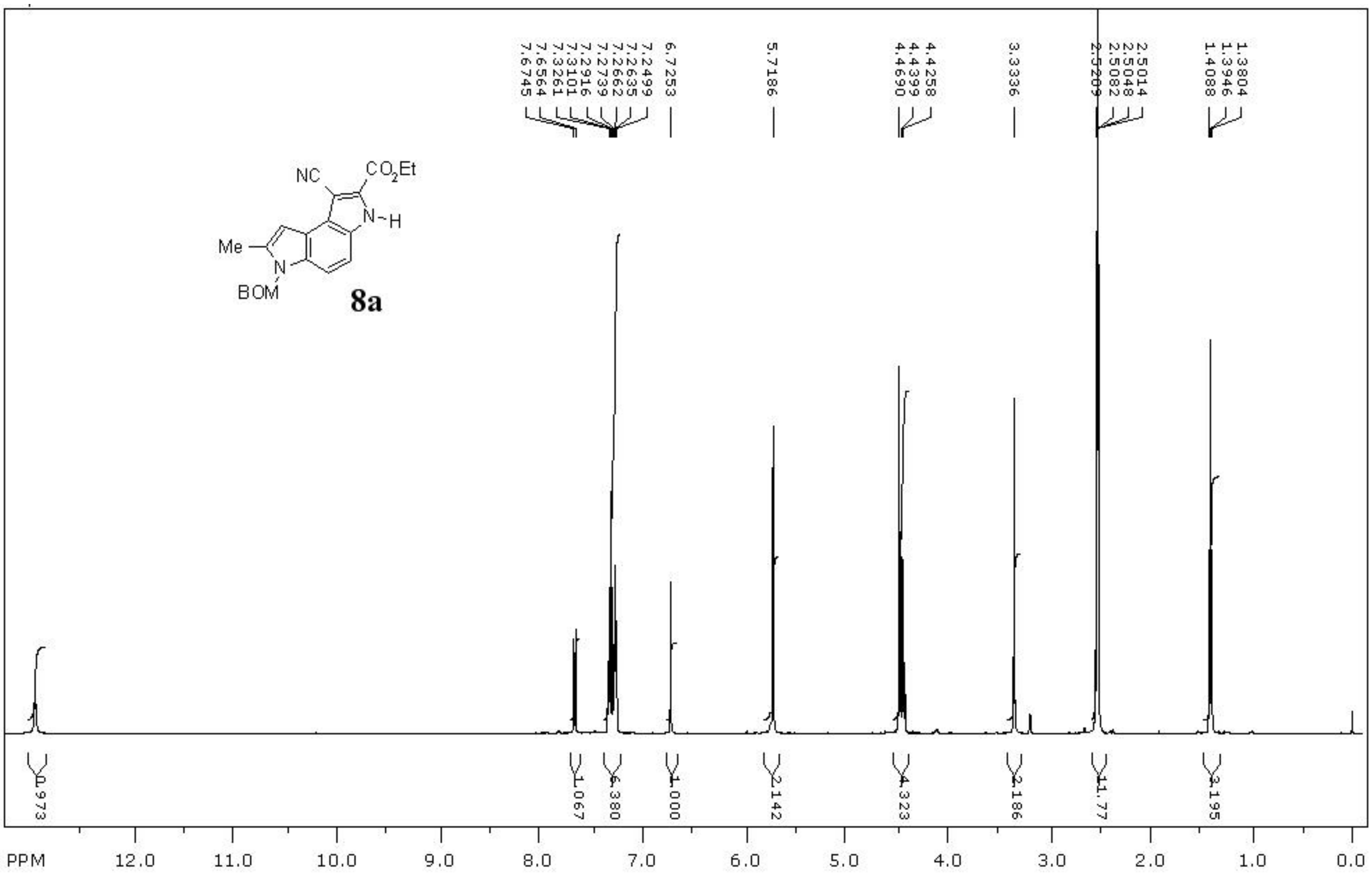

file: ...MIA\NMR\PIROLOINDOLE\ATIN-79\1\fid expt: <zg>
transmitter freq.: 500.133089 MHz
time domain size: 65536 points
width: 10330.58 Hz = 20.6557 ppm = 0.157632 Hz/pt
number of scans: 32

freq. of 0 ppm: 500.130003 MHz
processed size: 32768 complex points
LB: 0.000 GF: 0.0000
Hz/cm: 268.289 ppm/cm: 0.53643

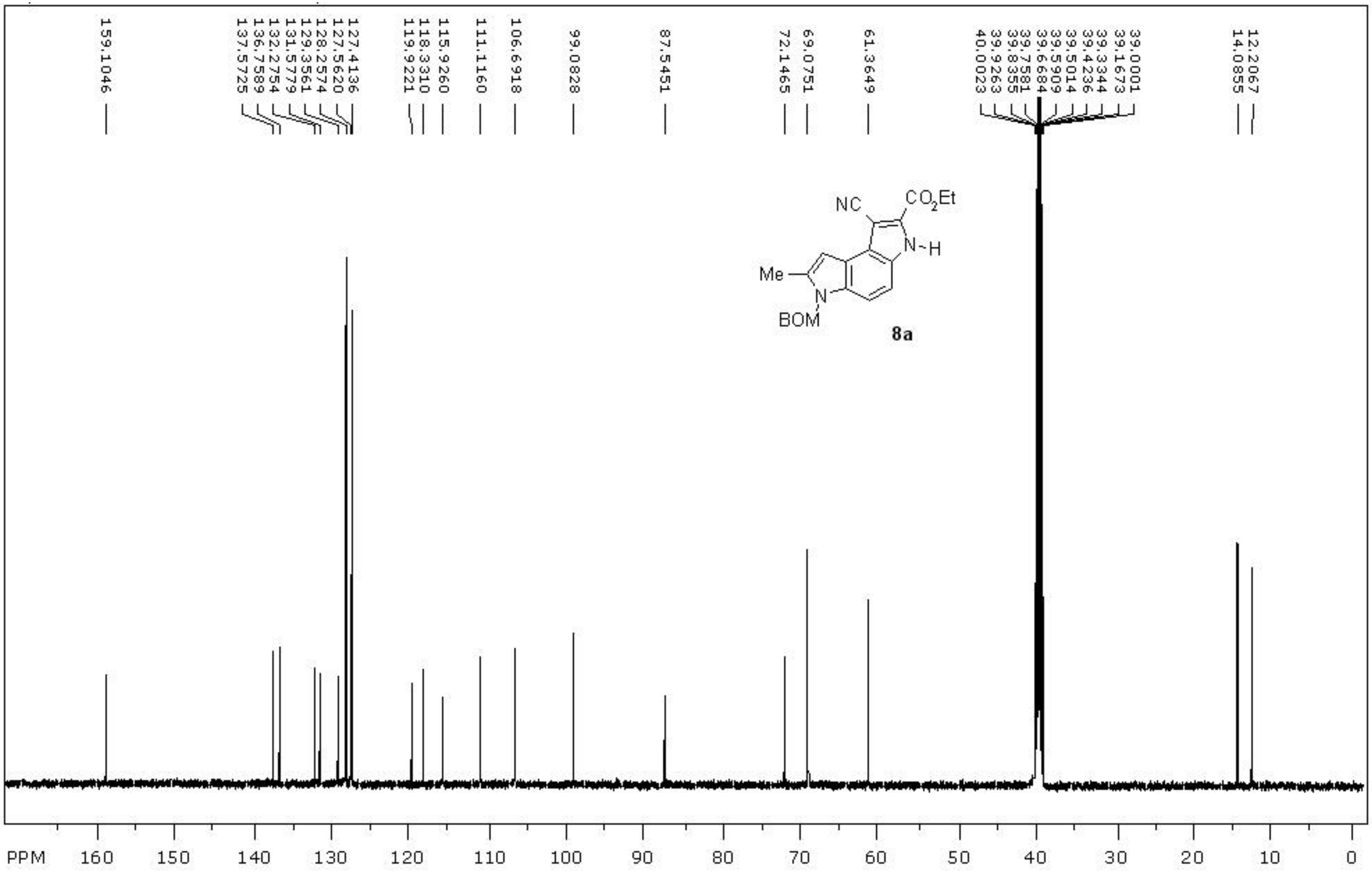

file: ...INDOLE\ATIN-38\ATIN-38-C13.fid\fid block # 1 expt: "s2pul"
transmitter freq.: 125.697101 MHz
time domain size: 80128 points
width: 32051.28 Hz = 254.9882 ppm = 0.400001 Hz/pt
number of scans: 1152

freq. of 0 ppm: 125.683335 MHz
processed size: 131072 complex points
LB: 1.000 GF: 0.0000
Hz/cm: 875.513 ppm/cm: 6.96526

PE SPECTRUM 2000 ICHO PAN Zespół Spektroskopii Optycznej Data: 2013-02-27

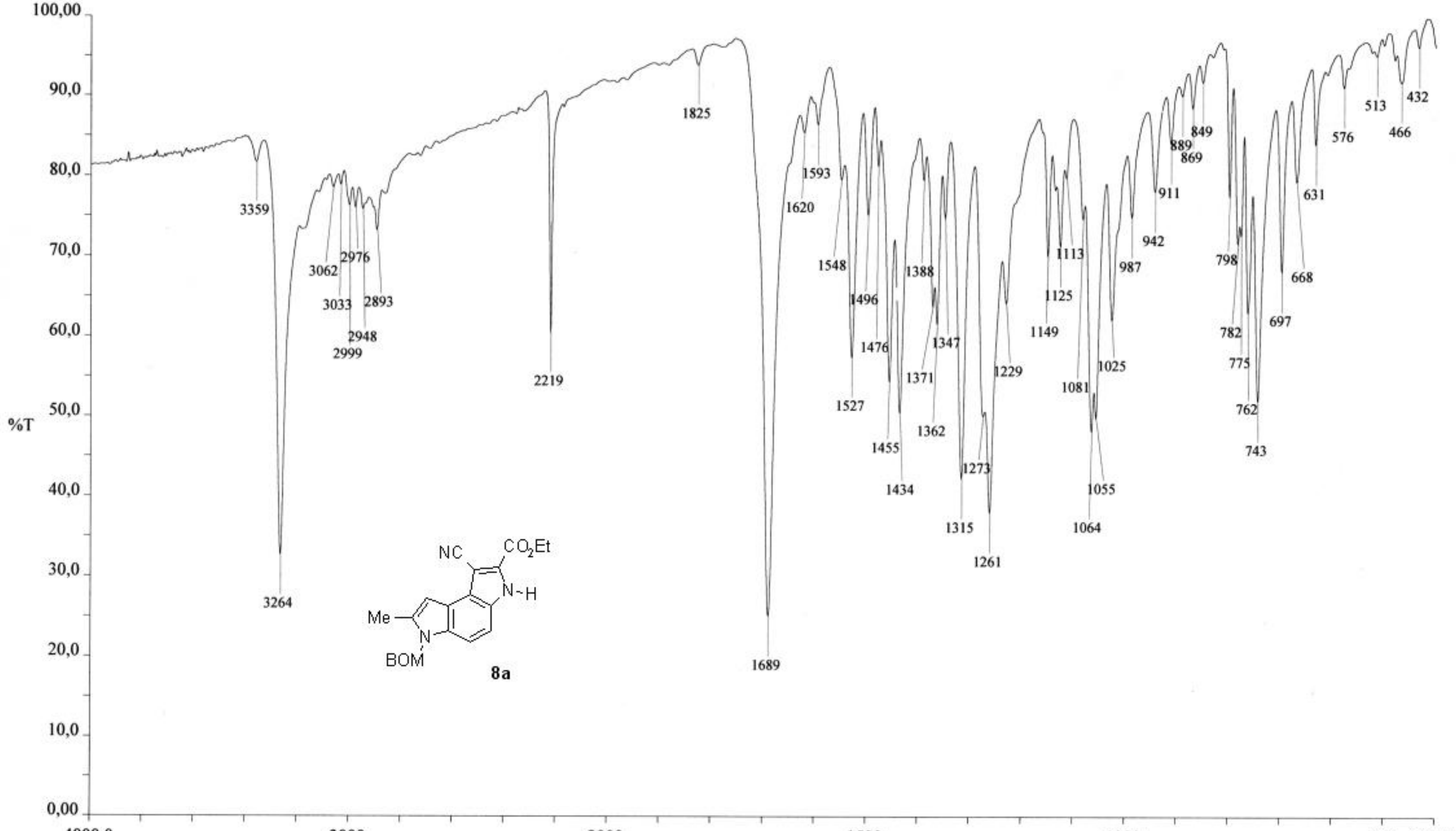

Opis: Pan Adam Trawczyński; ATIN-38; w KBr
Uwagi:

Numer widma: 13022706.sp
Operator: Alicja Dziedzic

File Name	:	E:\IChO\Z07_EG\at2928.ms2
Creation Date/Time	:	11-10-05 at 10:55:28
File Type	:	Lo-Res Data - Ctd (Magnet)
File Source	:	Acquired on MASPEC II system [II32/99D9]
File Title	:	ATIN-38 (EI 70 eV 33-800)
Operator	:	Małgorzata Grela
Instrument	:	AMD 604
Notes	:	A. Trawczyński

SCAN GRAPH. Flagging=Nominal M/z. Highlighting=Base Peak.
Scan 32#3:52. Entries=508. Base M/z=91.3. 100% Int.=99.7888. Temp =255.

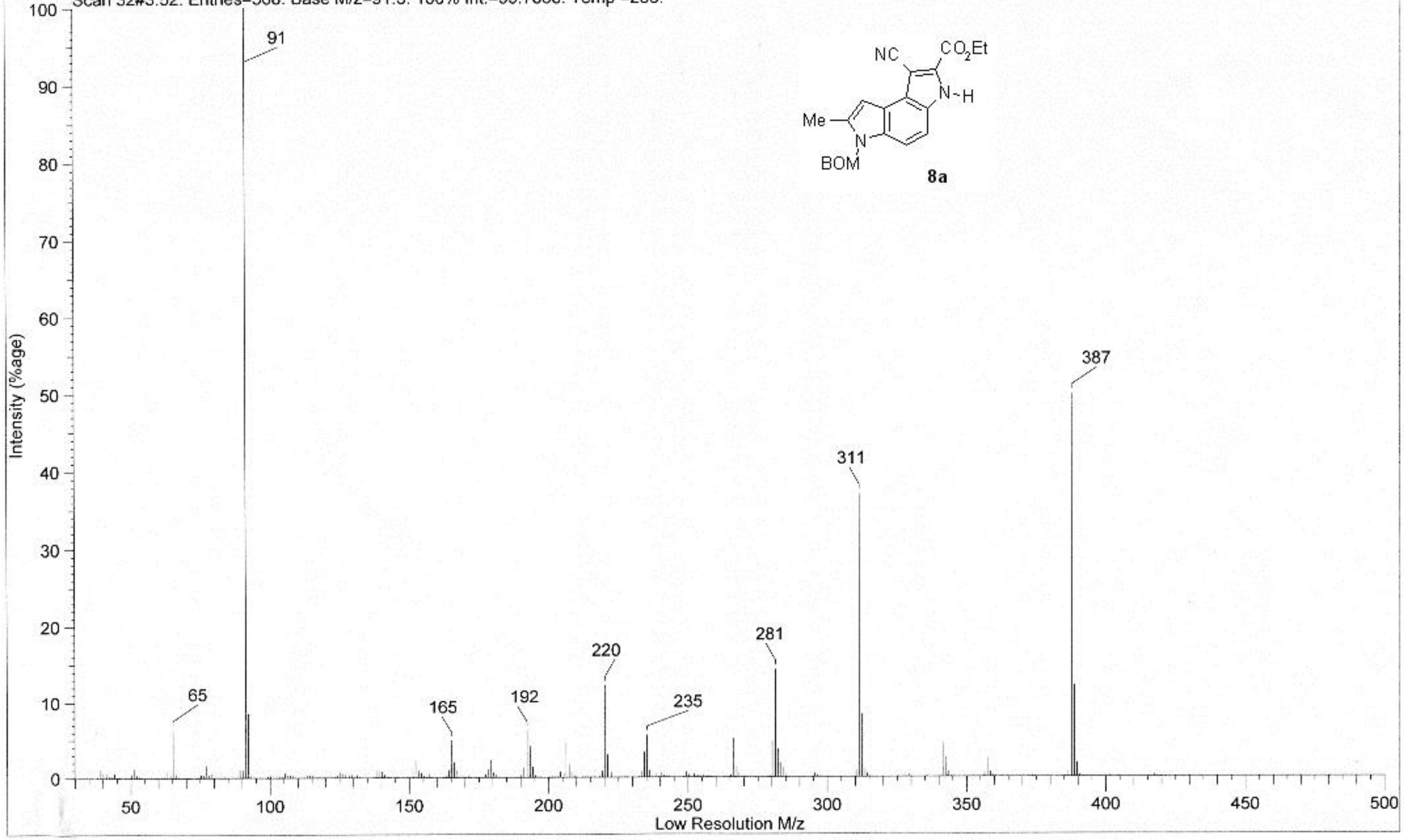

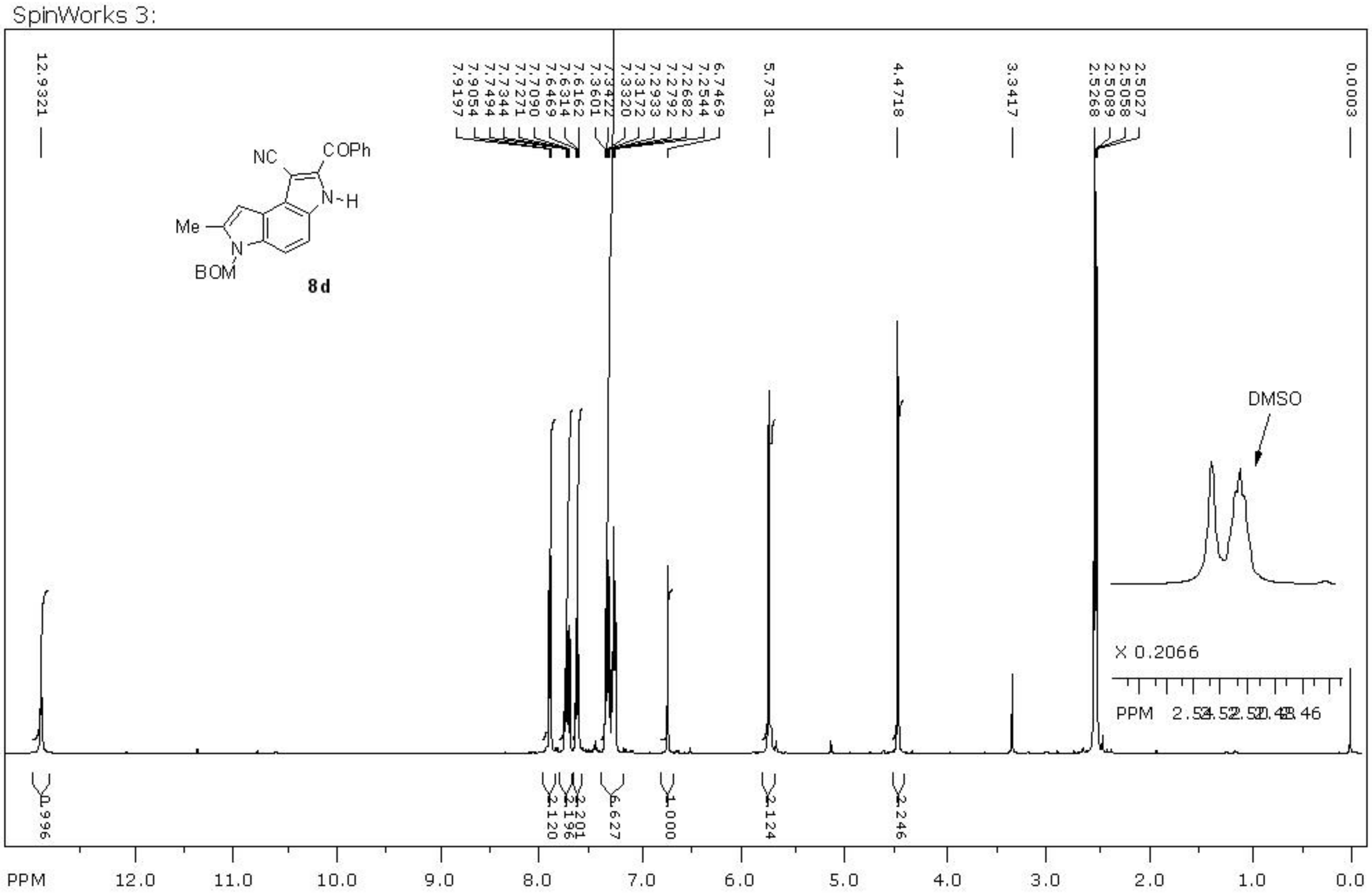

file: ...MIA\NMR\PIROLOINDOLE\ATIN-73\1\fid expt: <zg>
transmitter freq.: 500.133089 MHz
time domain size: 65536 points
width: 10330.58 Hz = 20.6557 ppm = 0.157632 Hz/pt
number of scans: 32

freq. of 0 ppm: 500.130003 MHz
processed size: 32768 complex points
LB: 0.000 GF: 0.0000
Hz/cm: 268.469 ppm/cm: 0.53680

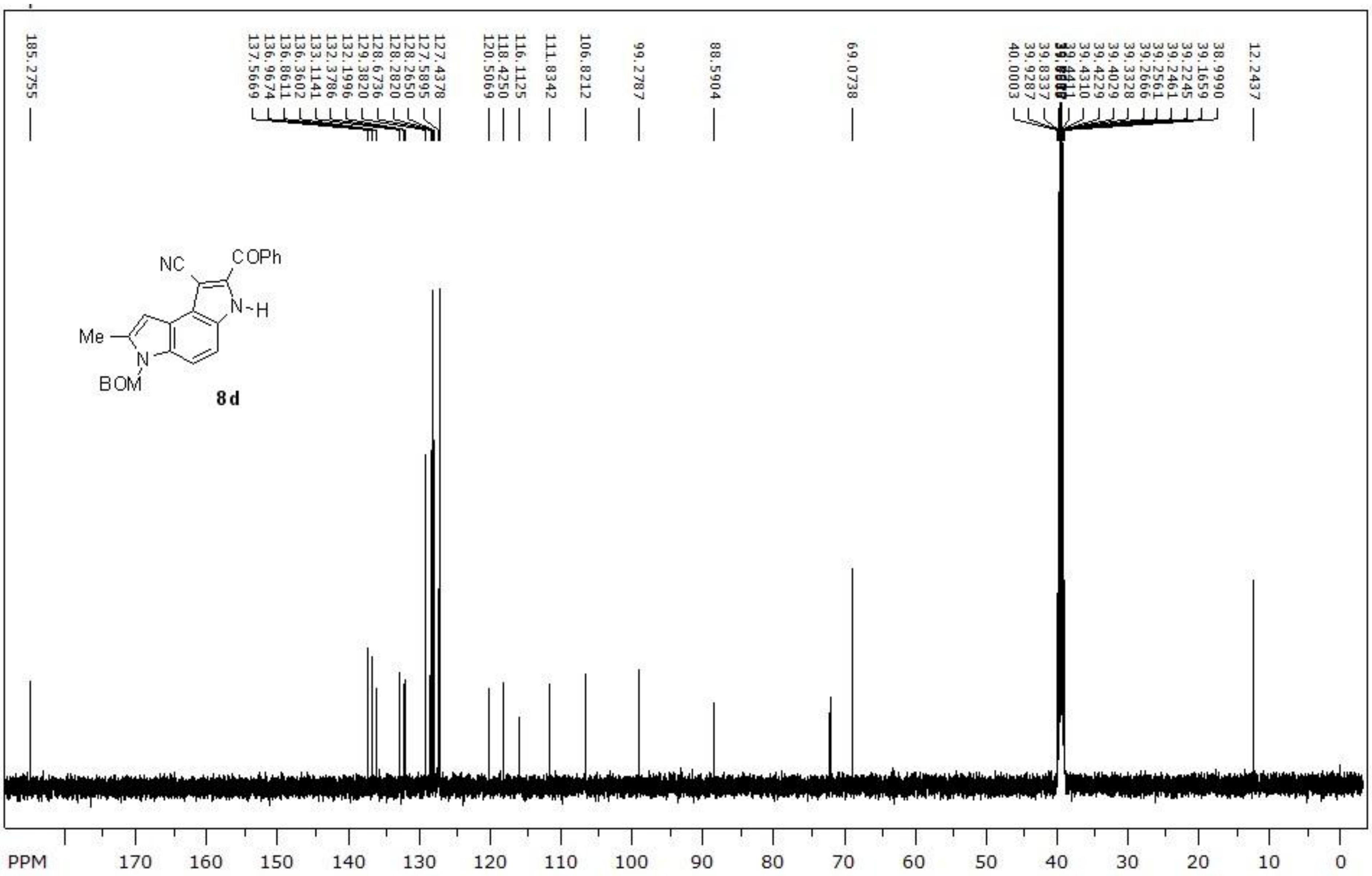

file: D:\NMR\PIROLOINDOLE\ATIN-73\2\fid expt: <zgpg>
transmitter freq.: 125.770364 MHz
time domain size: 65536 points
width: 32679.74 Hz = 259.8366 ppm = 0.498653 Hz/pt
number of scans: 1151

freq. of 0 ppm: 125.757852 MHz
processed size: 262144 complex points
LB: 0.500 GF: 0.0000
Hz/cm: 965.743 ppm/cm: 7.67862

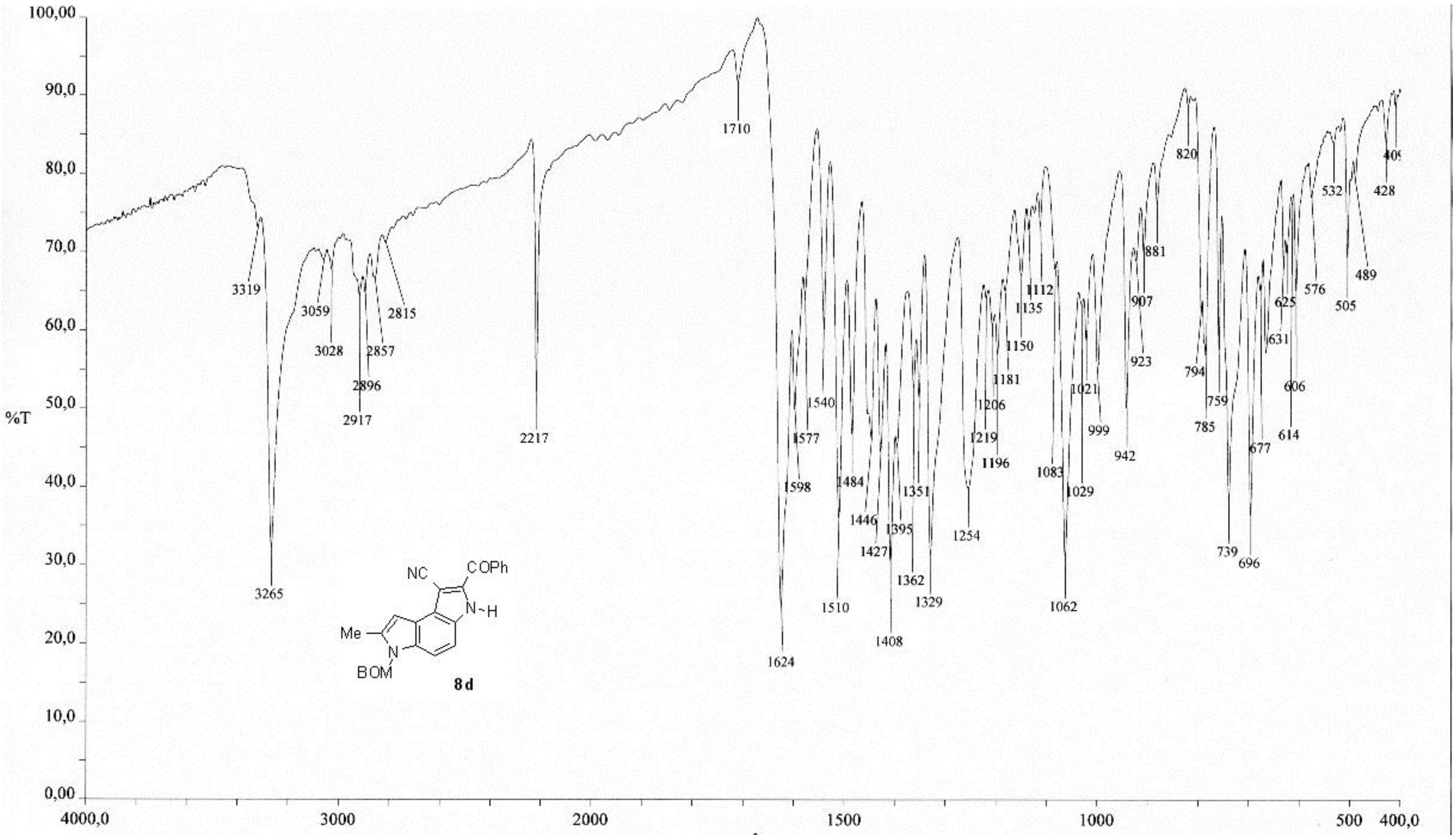

Opis: Pan Adam Trawczyński; ATIN-73; w KBr

Numer widma: 12032904.sp

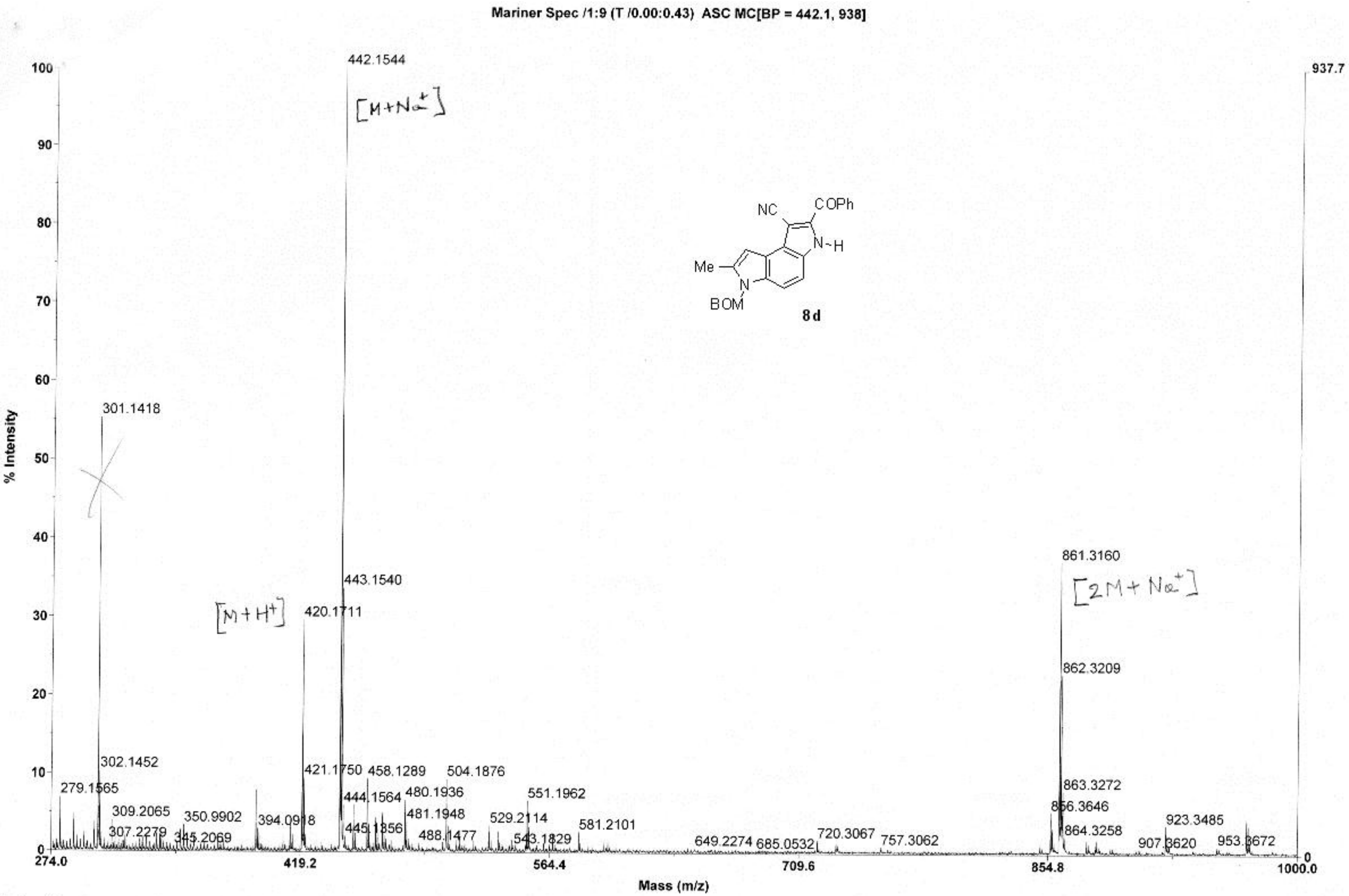

Mariner - Magda Kania - A. Trawczynski - ATIN-73 (MeOH, jony dodatnie)
Z:\...\AT869001.dat
Acquired: Mar 19 10:24:00 2012

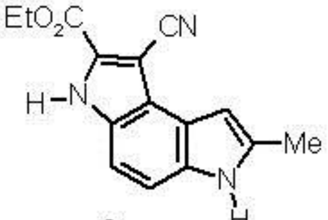

9a

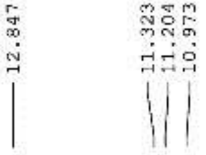
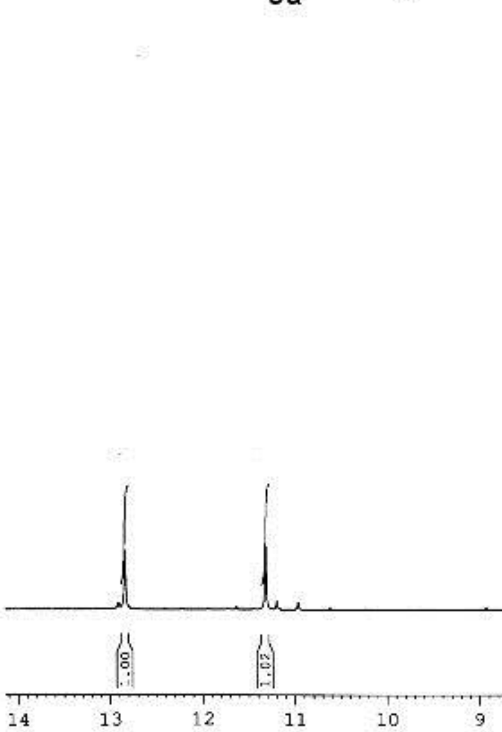
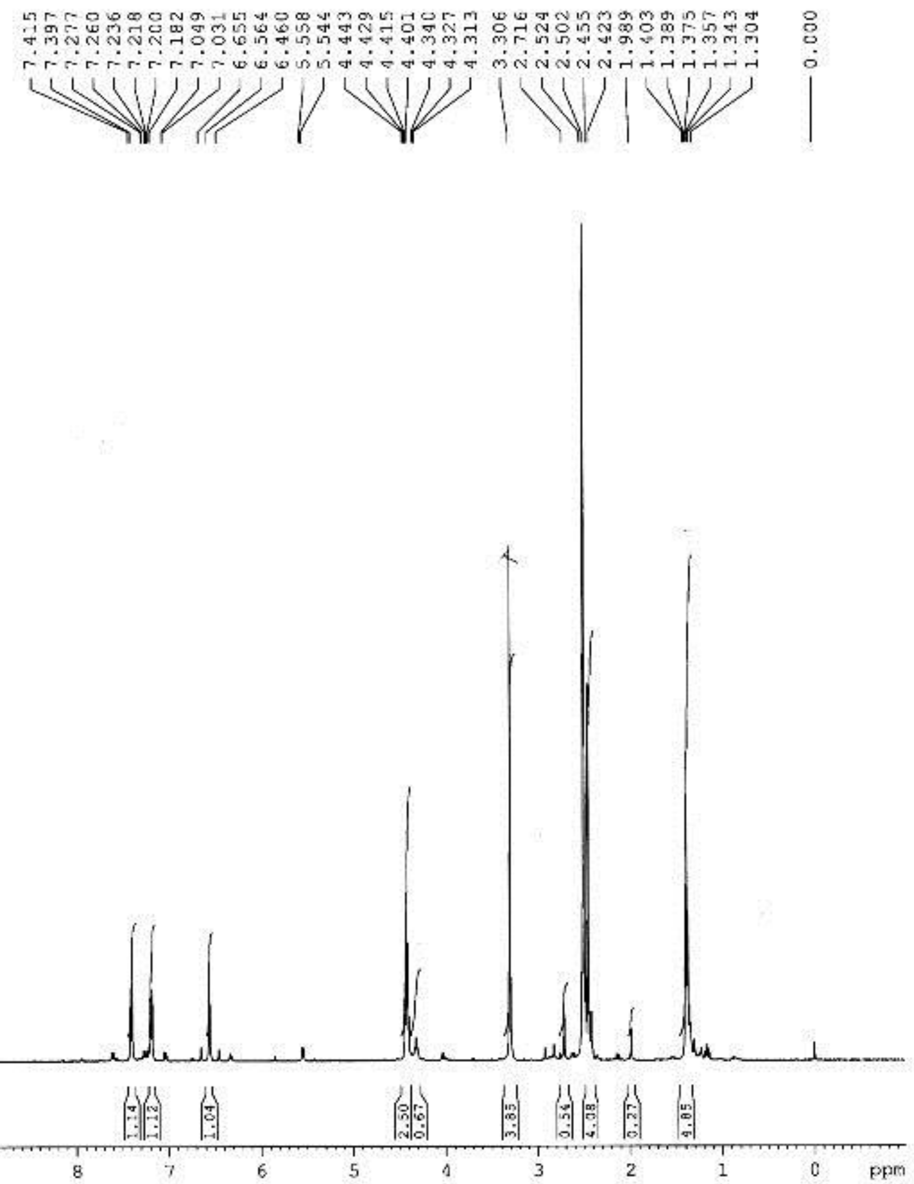

Current Data Parameters

NAME	IND-363-B
EXPNO	1
PROCNO	1

F2 - Acquisition Parameters

Date_	20130412
Time	11.51
INSTRUM	DRX
PROBHD	5 mm TBI 1H/13
PULPROG	zg30
TD	65536
SOLVENT	DMSO
NS	32
DS	0
SWH	10330.578 Hz
FIDRES	0.157632 Hz
AQ	3.1719923 sec
RG	287.4
DW	48.400 usec
DE	6.78 usec
TE	303.0 K
D1	1.00000000 sec
TD0	1

======== CHANNEL f1 ========

NUC1	1H
P1	8.20 usec
PL1	5.00 dB
SFO1	500.1330885 MHz

F2 - Processing parameters

SI	32768
SF	500.1300042 MHz
WDW	no
SSB	0
LB	0.00 Hz
GB	0
PC	1.00

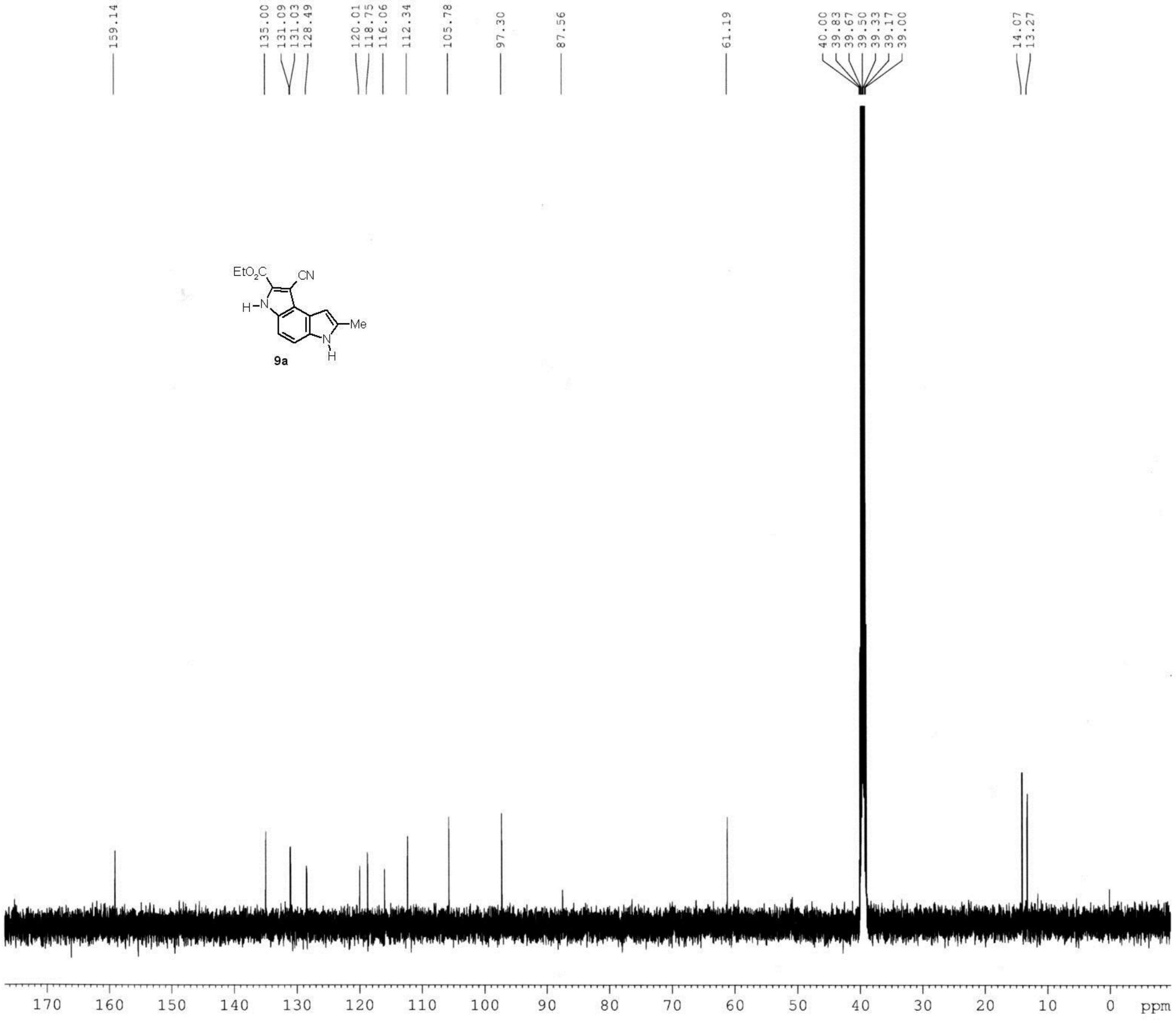

9a

BRUKER

```
Current Data Parameters
NAME            IND-363-B
EXPNO                   2
PROCNO                  1

F2 - Acquisition Parameters
Date_            20130412
Time                11.59
INSTRUM               DRX
PROBHD   5 mm TBI 1H/13
PULPROG              zgpg
TD                  65536
SOLVENT              DMSO
NS                   1555
DS                      4
SWH             32679.738 Hz
FIDRES           0.498653 Hz
AQ              1.0027508 sec
RG                  32768
DW                 15.300 usec
DE                   7.10 usec
TE                  303.0 K
D1             1.00000000 sec
d11            0.03000000 sec
DELTA          0.89999998 sec
TD0                     1

======== CHANNEL f1 ========
NUC1                  13C
P1                   5.00 usec
PL1                 -3.00 dB
SFO1          125.7703643 MHz

======== CHANNEL f2 ========
CPDPRG2           waltz16
NUC2                   1H
PCPD2               98.00 usec
PL2                  3.00 dB
PL12                23.00 dB
PL13                32.00 dB
SFO2          500.1320005 MHz

F1 - Acquisition parameters
ND0                     1
TD                    128
SFO1              500.132 MHz
FIDRES           7.812500 Hz
SW                  1.999 ppm
FnMODE                 QF

F2 - Processing parameters
SI                 262144
SF            125.7578564 MHz
WDW                    EM
SSB                     0
LB                   0.50 Hz
GB                      0
PC                   1.40

F1 - Processing parameters
SI                   1024
MC2                    QF
SF            500.1300000 MHz
WDW                  SINE
SSB                     0
LB                   0.30 Hz
GB                    0.1
```

JASCO FT/IR-6200 **IChO PAN Pracownia Spektroskopii Optycznej**

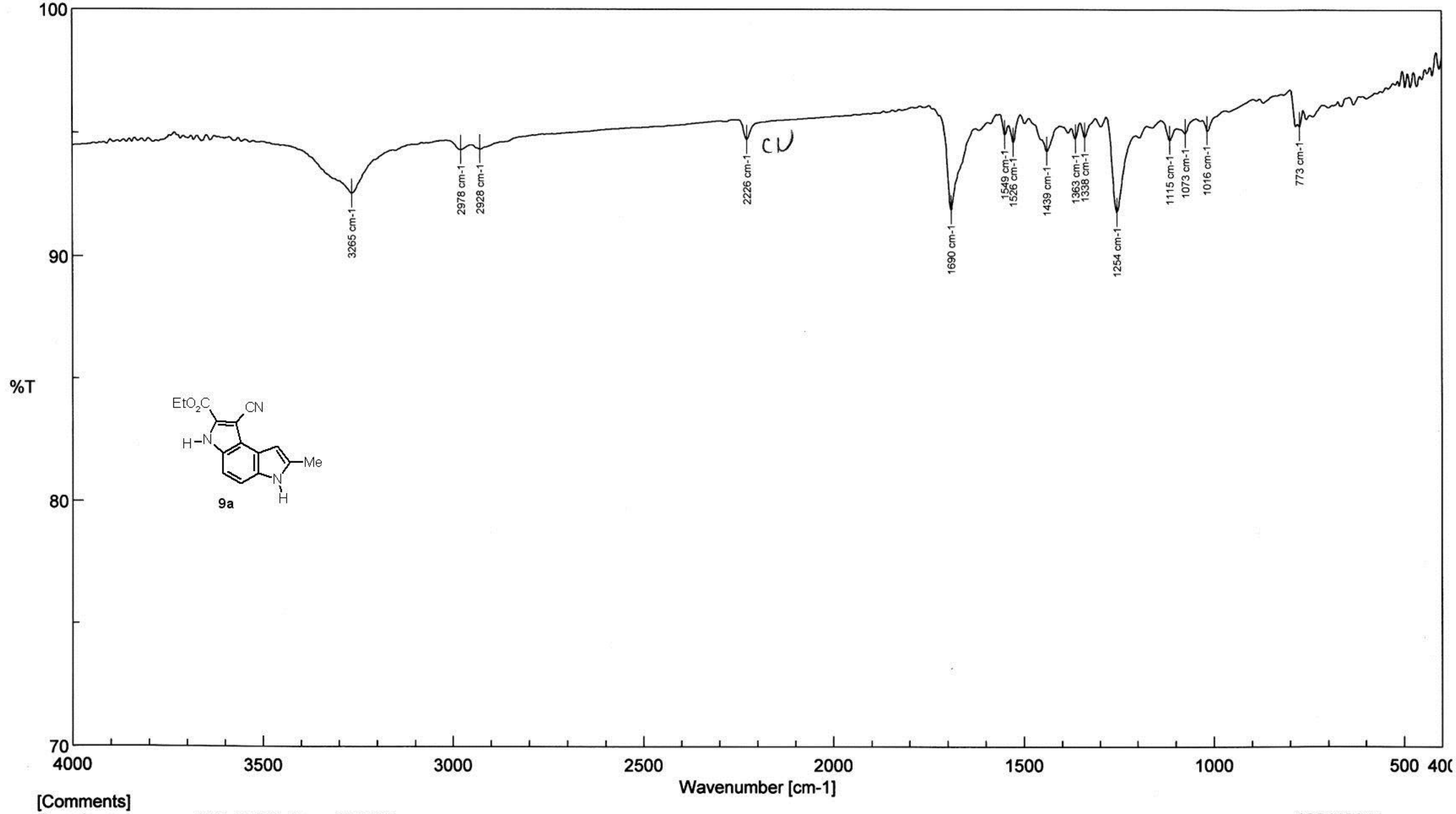

[Comments]

Sample name	IND-363/B; film z CH2Cl2
Comment	Pan Robert Bujok
User	Alicja
Division	Pracownia Spektroskopii Optycznej
Company	IChO PAN

0

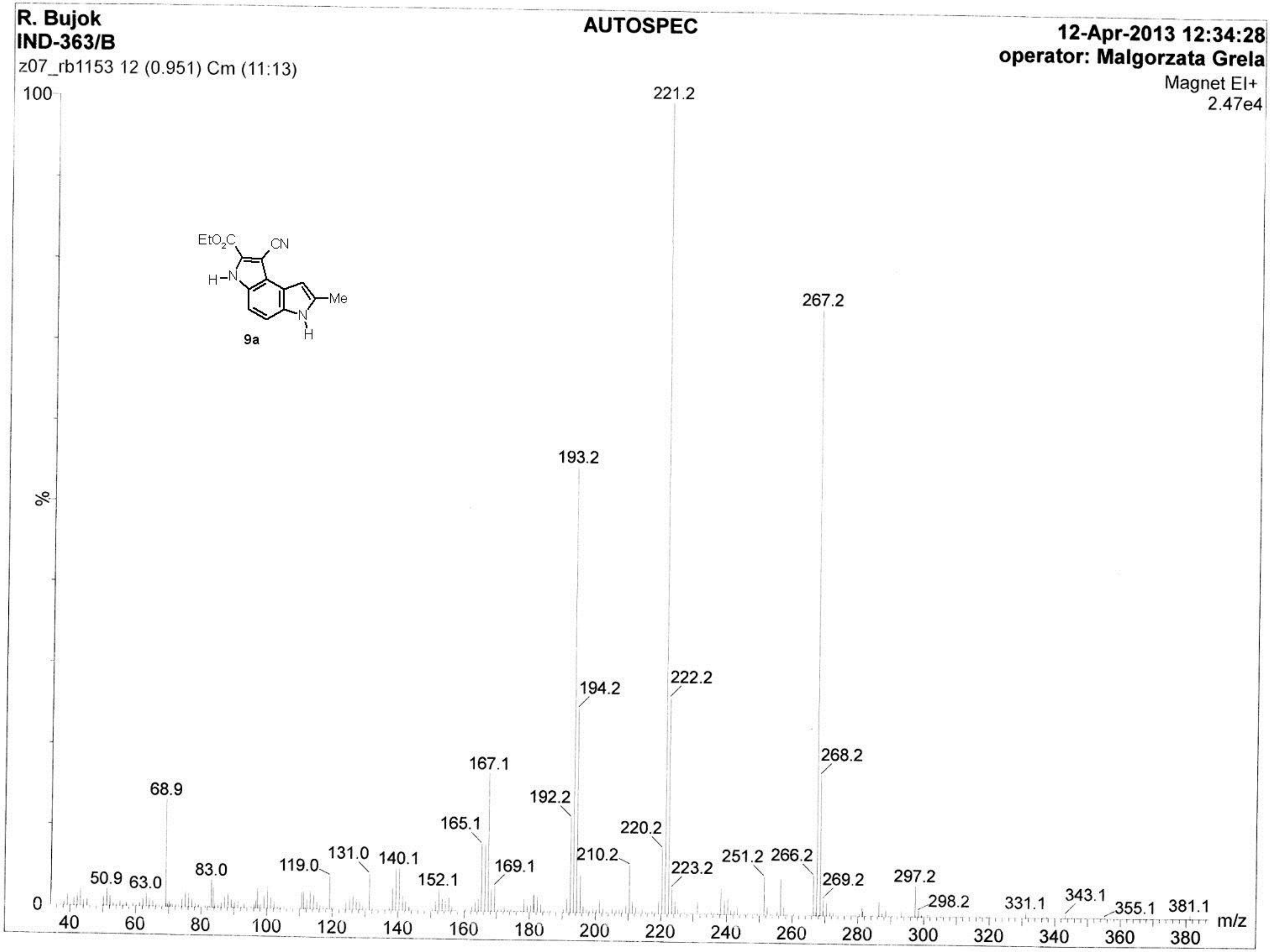
R. Bujok
IND-363/B
z07_rb1153 12 (0.951) Cm (11:13)
AUTOSPEC
12-Apr-2013 12:34:28
operator: Malgorzata Grela
Magnet EI+
2.47e4
100
%
0
EtO2C
CN
H
N
Me
N
H
9a
221.2
267.2
193.2
194.2
222.2
268.2
167.1
68.9
192.2
165.1
220.2
210.2
223.2
251.2
266.2
169.1
131.0
140.1
119.0
83.0
50.9
63.0
152.1
269.2
297.2
298.2
331.1
343.1
355.1
381.1
40
60
80
100
120
140
160
180
200
220
240
260
280
300
320
340
360
380
m/z

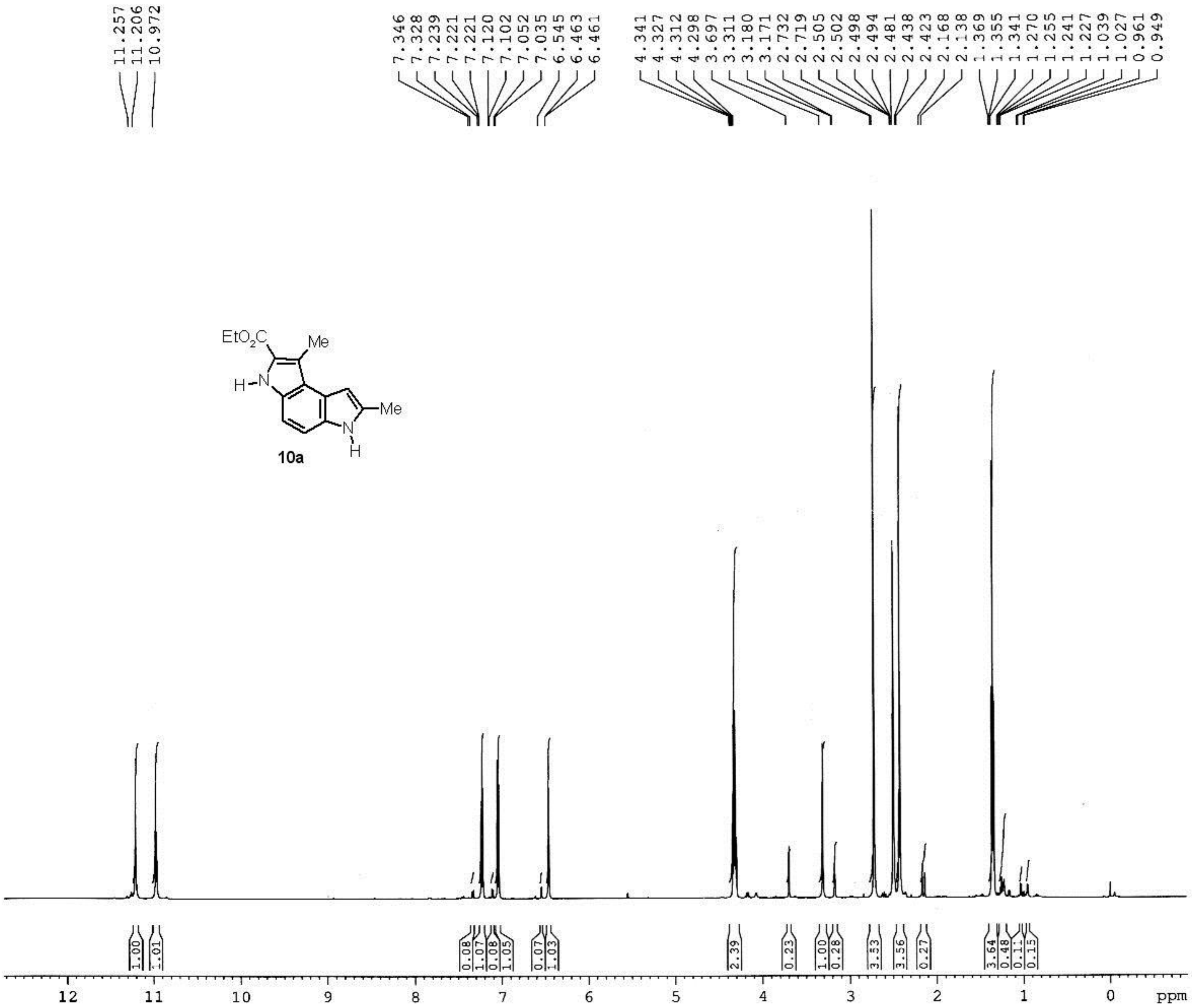

10a

Current Data Parameters
NAME IND-361-K
EXPNO 1
PROCNO 1

F2 - Acquisition Parameters
Date_ 20130411
Time 8.59
INSTRUM DRX
PROBHD 5 mm TBI 1H/13
PULPROG zg30
TD 65536
SOLVENT DMSO
NS 32
DS 0
SWH 10330.578 Hz
FIDRES 0.157632 Hz
AQ 3.1719923 sec
RG 161.3
DW 48.400 usec
DE 6.78 usec
TE 303.0 K
D1 1.00000000 sec
TD0 1

======== CHANNEL f1 ========
NUC1 1H
P1 8.20 usec
PL1 5.00 dB
SFO1 500.1330885 MHz

F2 - Processing parameters
SI 32768
SF 500.1300065 MHz
WDW no
SSB 0
LB 0.00 Hz
GB 0
PC 1.00

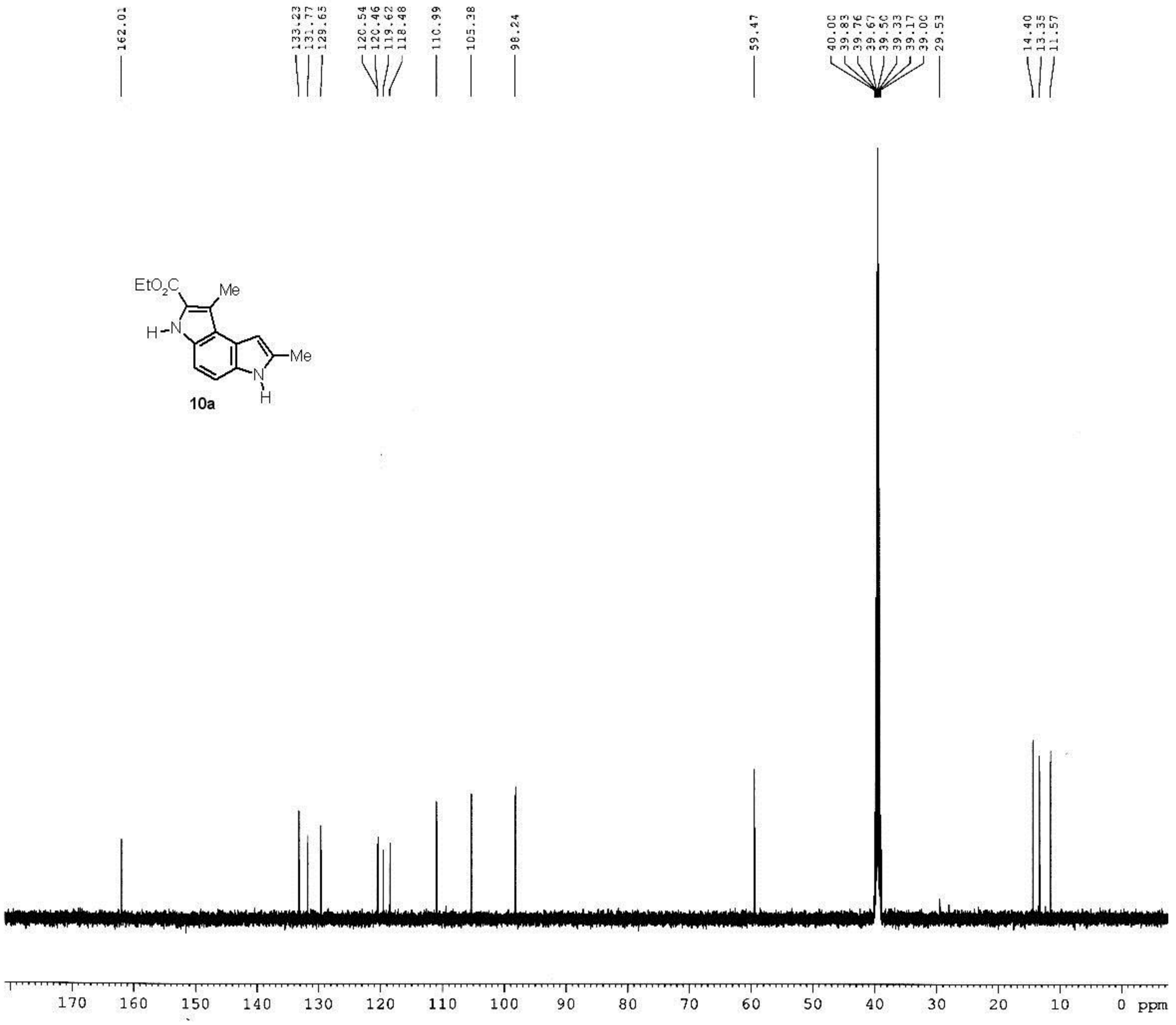


```
Current Data Parameters
NAME          IND-361-K
EXPNO                 2
PROCNO                1

F2 - Acquisition Parameters
Date_          20130411
Time               9.22
INSTRUM             DRX
PROBHD   5 mm TBI 1H/13
PULPROG            zgpg
TD                65536
SOLVENT            DMSO
NS                 1125
DS                    4
SWH           32679.738 Hz
FIDRES         0.498653 Hz
AQ            1.0027508 sec
RG                32768
DW               15.300 usec
DE                 7.10 usec
TE                303.0 K
D1           1.00000000 sec
d11          0.03000000 sec
DELTA        0.89999998 sec
TD0                   1

======== CHANNEL f1 ========
NUC1                13C
P1                 5.00 usec
PL1               -3.00 dB
SFO1        125.7703643 MHz

======== CHANNEL f2 ========
CPDPRG2         waltz16
NUC2                 1H
PCPD2             98.00 usec
PL2                3.00 dB
PL12              23.00 dB
PL13              32.00 dB
SFO2        500.1320005 MHz

F1 - Acquisition parameters
ND0                   1
TD                  128
SFO1            500.132 MHz
FIDRES         7.812500 Hz
SW                1.999 ppm
FnMODE               QF

F2 - Processing parameters
SI               262144
SF          125.7578561 MHz
WDW                  EM
SSB                   0
LB                 0.50 Hz
GB                    0
PC                 1.40

F1 - Processing parameters
SI                 1024
MC2                  QF
SF          500.1300000 MHz
WDW                SINE
SSB                   0
LB                 0.30 Hz
GB                  0.1
```

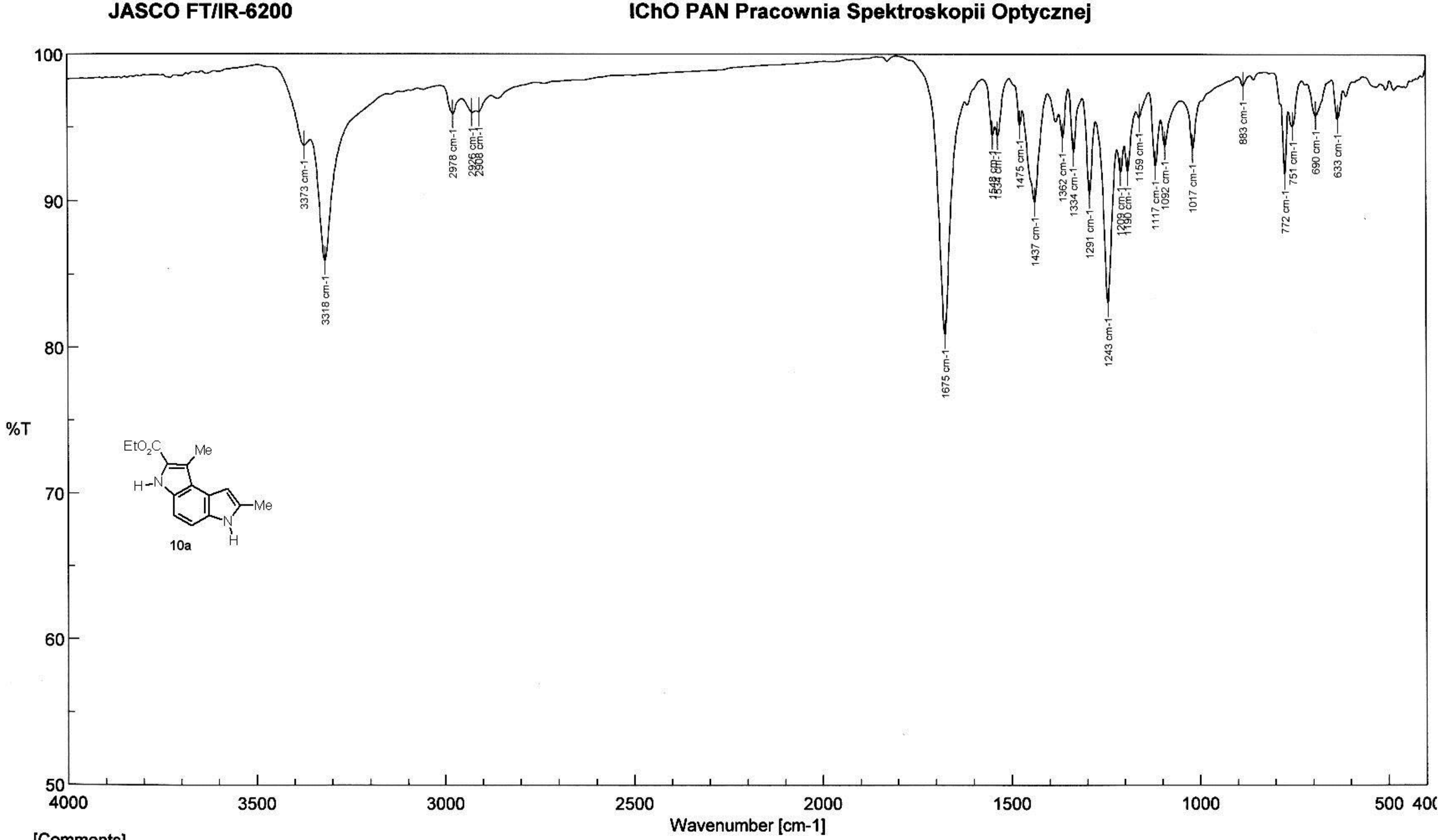

[Comments]

Sample name	IND-361/K; w KBr
Comment	Pan Robert Bujok
User	Alicja
Division	Pracownia Spektroskopii Optycznej
Company	IChO PAN
